# GPR126 is a specifier of blood-brain barrier formation in the mouse central nervous system

**DOI:** 10.1172/JCI165368

**Published:** 2024-06-06

**Authors:** Nikolaos Kakogiannos, Anna Agata Scalise, Emanuele Martini, Claudio Maderna, Andrea Francesco Benvenuto, Michele D’Antonio, Laura Carmignani, Serena Magni, Giorgia Serena Gullotta, Maria Grazia Lampugnani, Fabio Iannelli, Galina V. Beznoussenko, Alexander A. Mironov, Camilla Cerutti, Katie Bentley, Andrew Philippides, Federica Zanardi, Marco Bacigaluppi, Sara Sigismund, Claudia Bassani, Cinthia Farina, Gianvito Martino, Marco De Giovanni, Elisabetta Dejana, Matteo Iannacone, Donato Inverso, Monica Giannotta

**Affiliations:** 1 IFOM ETS, the AIRC Institute of Molecular Oncology, Milan, Italy.; 2 Department of Oncology and Hematology-Oncology, Università degli Studi di Milano, Milan, Italy.; 3 Department of Experimental Oncology, European Institute of Oncology (IEO) IRCCS, Milan, Italy.; 4 Division of Immunology, Transplantation, and Infectious Diseases, IRCCS San Raffaele Scientific Institute, Milan, Italy.; 5 Neuroimmunology Unit, Institute of Experimental Neurology, IRCCS, San Raffaele Hospital, Milan, Italy.; 6 The Francis Crick Institute, London, United Kingdom.; 7 Department of Informatics, King’s College London, London, United Kingdom.; 8 Department of Informatics, University of Sussex, Brighton, United Kingdom.; 9 Vita-Salute San Raffaele University, Milan, Italy.; 10 Immunobiology of Neurological Disorders Unit, Institute of Experimental Neurology, IRCCS San Raffaele Scientific Institute, Milan, Italy.

**Keywords:** Angiogenesis, Vascular biology, Cardiovascular disease, Endothelial cells, G protein&ndash;coupled receptors

## Abstract

The blood-brain barrier (BBB) acquires unique properties to regulate neuronal function during development. The formation of the BBB, which occurs in tandem with angiogenesis, is directed by the Wnt/β-catenin signaling pathway. Yet the exact molecular interplay remains elusive. Our study reveals the G protein–coupled receptor GPR126 as a critical target of canonical Wnt signaling, essential for the development of the BBB’s distinctive vascular characteristics and its functional integrity. Endothelial cell–specific deletion of the *Gpr126* gene in mice induced aberrant vascular morphogenesis, resulting in disrupted BBB organization. Simultaneously, heightened transcytosis in vitro compromised barrier integrity, resulting in enhanced vascular permeability. Mechanistically, GPR126 enhanced endothelial cell migration, pivotal for angiogenesis, acting through an interaction between LRP1 and β_1_ integrin, thereby balancing the levels of β_1_ integrin activation and recycling. Overall, we identified GPR126 as a specifier of an organotypic vascular structure, which sustained angiogenesis and guaranteed the acquisition of the BBB properties during development.

## Abstract 2

The G protein–coupled receptor GPR126 enhances endothelial cell migration via LRP1 and β_1_ integrin and contributes to blood-brain barrier development in mice.

## Graphical abstract

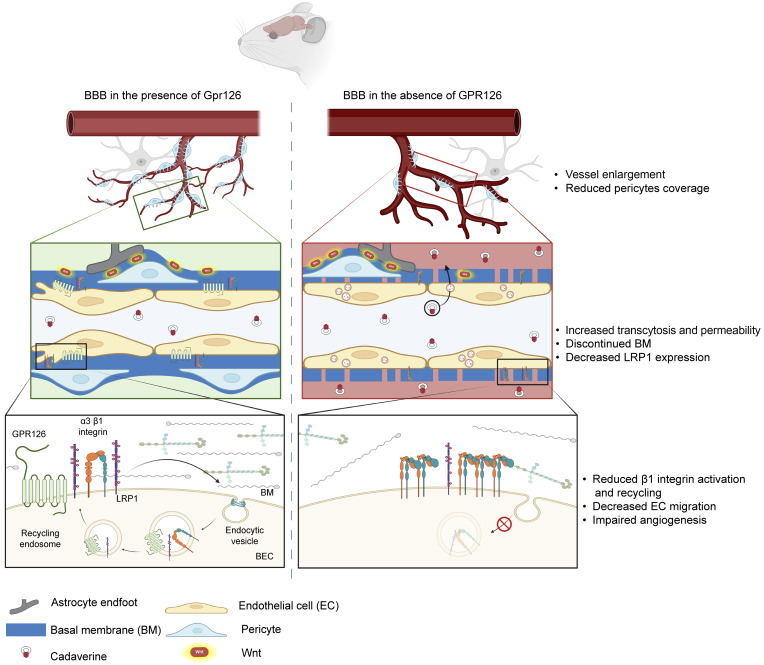

## Introduction

The blood-brain barrier (BBB) separates the neural tissue from the blood circulation. The BBB is formed by a single layer of the endothelial cells (ECs) that line the blood vessel wall, which are surrounded by pericytes, astrocytes, and vascular smooth muscle cells that are embedded in the basement membrane (BM). Together, these form a structure that is commonly known as the neurovascular unit (NVU) (1). The BBB ECs and all the cells that form the NVU maintain the homeostatic milieu to allow correct neuronal function.

Impairment of the BBB and increased permeability are observed in various conditions, including stroke, multiple sclerosis, HIV encephalitis, age-related dementia, and Alzheimer’s disease (2). BBB ECs form an active permeability barrier with unique biological features and transport systems. Firstly, BBB ECs express specialized tight junction (TJ) proteins like claudins, occludin, and junctional adhesion molecules, which tightly regulate molecule passage from the bloodstream into brain tissue and are connected to the actin cytoskeleton via proteins like ZO-1 (3, 4). Additionally, adherens junction proteins such as VE-cadherin strengthen bonds between neighboring ECs (5). Secondly, BBB ECs express exclusive transporters to regulate influx and efflux of specific substrates (6). For example, LRP1 mediates the crossing of amyloid-β, a peptide in Alzheimer’s disease, from the brain parenchyma into circulation (7). Thirdly, BBB ECs have low rates of transcellular vesicle trafficking, termed transcytosis, limiting transcellular transport through the vessel wall. Finally, to inhibit immune cell entry into the brain, BBB ECs express low levels of leukocyte adhesion molecules (8).

However, these specific barrier properties of ECs are not intrinsic, as, in contexts like bone marrow and liver, the vasculature acquires opposite features with open fenestration and limited BM. Initially, when brain ECs enter the central nervous system (CNS), they lack inherent barrier properties. Thus, BBB formation is a gradual process starting during embryonic development, with brain angiogenesis beginning at embryonic day 9.5 (E9.5) and completing during early postnatal stages, up to 25 days in mice (9). These properties are acquired through close interactions and crosstalk between ECs and surrounding cells that form the NVU (1, 2).

The NVU BM provides structural support and serves as a hub for intercellular communication and signaling, composed of structural proteins like collagen IV, fibronectin, laminins, and other glycoproteins. Collagen IV and fibronectin, secreted by ECs, pericytes, and astrocytes, are essential for embryonic survival (10). Laminins, with various isoforms, maintain vessel and BBB integrity (11).

The BBB is dynamic and regulated by interactions between its cellular and BM components, along with their receptors like integrins (12). Brain capillary ECs, pericytes, and astrocytes express different β_1_ integrin isoforms (13, 14) that bind to the BM and trigger signaling cascades to regulate cell survival, proliferation, and migration.

Canonical Wnt signaling is pivotal in regulating brain angiogenesis and BBB formation. Wnt ligands from neural cells activate this pathway in ECs by binding to frizzled receptors (15) and coreceptors LRP5 and LRP6, initiating β catenin–dependent pathways (16–18). This activation enhances TJs and BBB formation by upregulating barrier-related genes like solute carrier family 2 member 1 (*Slc2a1*) and claudin-5 (*Cldn5*) (16, 17). Deficiency in endothelial Wnt/β-catenin signaling specifically impacts cerebrovascular and BBB development without affecting other organ and tissue functions (16, 19, 20).

Therefore, we hypothesized that molecular targets of Wnt/β-catenin signaling in brain ECs could regulate BBB development. Downstream factors of this pathway, such as SRY-box transcription factor 17 (SOX17) (21) and fibroblast growth factor–binding protein 1 (FGFBP1) (22), promote BBB development, while forkhead box F2 (FOXF2) (23) maintains BBB characteristics. However, understanding of how canonical Wnt activation controls a functional BBB and links brain angiogenesis to BBB genesis is limited. To investigate, we activated Wnt/β-catenin signaling in murine brain microvasculature ECs using the Wnt ligand Wnt3a.

Microarray analyses unveiled the adhesion G protein–coupled receptor (GPCR) 126 (*Gpr126*) as a gene regulated by Wnt/β-catenin signaling. GPR126, belonging to the adhesion subfamily of GPCRs, shares a seven-transmembrane domain with other GPCRs but is distinguished by its long, heavily glycosylated N-terminal regions containing various adhesion domains separated from the seven-transmembrane domain helix by a GPCR autoproteolysis-inducing domain (GAIN). GPR126 can be cleaved at a GPCR proteolytic site in the GAIN domain, yielding N-terminal and C-terminal fragments (24). While the C-terminal fragment engages α subunits of heterotrimeric G proteins to transduce extracellular stimuli intracellularly, the N-terminal fragment forms docking sites for extracellular matrix proteins (25–27). Notably, major BM components in the BBB, such as laminin-211, collagen IV, and the prion protein, serve as extracellular ligands for the N-terminal fragment of GPR126, triggering cAMP signaling and inducing biological effects in Schwann cells through Gα_s_ coupling (28–30). Moreover, the C-terminal fragment of GPR126 contains a binding site for progesterone and 17-hydroxyprogesterone, initiating downstream Gα_i_ signaling (31).

Furthermore, GPR126 plays crucial roles in various organ and tissue development, including Schwann cells and peripheral nervous system myelination, bone formation, inner ear development, and placental development (32–34). Human GPR126 mutations are associated with non-neurological diseases like adolescent idiopathic scoliosis (35), arthrogryposis multiplex congenita (36), and carcinogenesis (31). While GPR126 regulates physiological and pathological angiogenesis by modulating VEGFR2 expression and activity, its function at the BBB is not fully understood (37).

In this study, we demonstrated that canonical Wnt signaling regulates GPR126 expression in the brain, showing a dynamic pattern correlated with BBB development. EC-specific *Gpr126* inactivation in mice (*Gpr126^iECKO^*) resulted in aberrant brain vascular morphogenesis, characterized by impaired BM protein deposition and deficient pericyte recruitment during vessel growth. Additionally, GPR126 deficiency led to compromised BBB integrity, allowing cadaverine accumulation due to increased transcellular transport. RNA sequencing analysis of brain ECs confirmed the essential role of GPR126 in regulating transcriptional programs necessary for proper BBB development. It also indicated that loss of BBB characteristics correlated with defective angiogenesis. Indeed, *Gpr126^iECKO^* mice exhibited defective sprouting, migration, and proliferation during brain and retina angiogenesis. While unraveling the molecular mechanisms of angiogenesis, we identified LRP1 as a target of GPR126 both postnatally and embryonically at E14.5, a stage characterized by the angiogenic phase of the BBB development. Finally, GPR126 interacts with LRP1 and β_1_ integrin, regulating EC migration during angiogenesis by balancing the levels of β_1_ integrin activation and recycling.

Overall, we identified GPR126 as a specifier of an organotypic vascular structure, supporting angiogenesis and ensuring BBB property acquisition during development.

## Results

### GPR126 is a target of Wnt/β-catenin signaling in the brain microvasculature.

**Figure 1:**
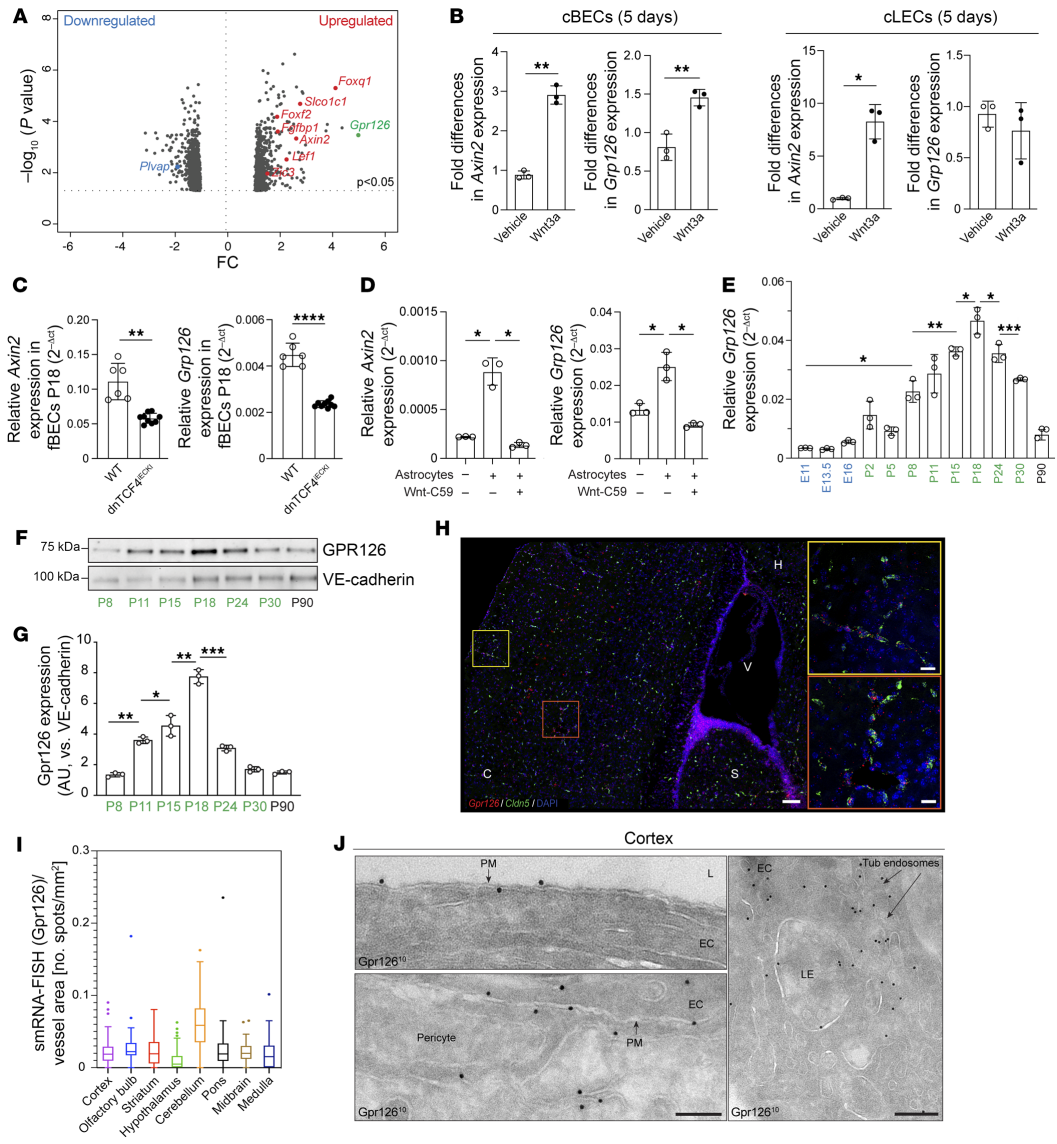
GPR126 is a target of Wnt/β-catenin signaling and is expressed in brain vasculature during BBB development. (**A**) Volcano plot showing transcriptional changes in cBECs exposed to Wnt3a-conditioned medium versus control for 5 days. Genes with significant alterations (*P* < 0.05) are depicted (Fisher’s least significant difference test). Red dots, upregulated gene targets of Wnt/β-catenin; blue dots, downregulated genes. *Gpr126* is highlighted in green. (**B**) Real-time qPCR of *Axin2* and *Gpr126* expression in cBECs and cLECs from adult WT mice treated with recombinant Wnt3a or control. (**C**) Real-time qPCR of *Axin2* and *Gpr126* in fBECs from mice at P18 (*n* = 6 WT, *n* = 9 *dnTCF4^iECKI^* mice). (**D**) Real-time qPCR of *Axin2* and *Gpr126* in iBECs with or without primary neonatal astrocytes and treated with vehicle or Wnt-C59 (*n* = 3). (**E**) Real-time qPCR of *Gpr126* expression in fBECs from WT mice during embryonic (E11–E16) and postnatal (P2–P30) stages and in the adult (P90) (*n* = 8 embryos, *n* = 5 postnatal, *n* = 3 adults). (**F** and **G**) Immunoblotting for GPR126 in fBECs from different postnatal stages and adulthood (P8–P30 and P90), quantified by GPR126/VE-cadherin ratios (*n* = 3 WT mice). (**H** and **I**) FISH confocal imaging for *Gpr126* (red) and *Cldn5* (green) mRNA in mouse cortex at P18, quantified by *Gpr126* single-molecule RNA (smRNA) per vessel area (number of spots/μm^2^). Each symbol represents a field (3–4 fields per region, *n* = 4 WT mice). C, cortex; S, striatum; V, ventricle; H, hippocampus. (**J**) Electron microscopy of GPR126 immunogold-labeled (10 nm) cryosection of brain capillaries from WT mouse cortex at P18. Top: Luminal plasma membrane (PM). Bottom: Abluminal EC and pericyte plasma membrane (PM). Right: Late endosome (LE). L, lumen. Scale bars: 200 nm. Data are shown as means ± SD. (**B** and **D**–**G**) Each symbol represents an experiment; Brown-Forsythe and Welch’s ANOVA, Dunnett’s T3 multiple-comparison tests. (**B** and **C**) Unpaired *t* tests with Welch’s correction. *P < 0.05; **P < 0.01; ***P < 0.001; ****P < 0.0001.

Wnt/β-catenin signaling has emerged as a fundamental determinant for acquisition of the specialized phenotype of brain ECs that results in the establishment of the BBB (16, 17, 19). This suggests that the molecular targets of Wnt/β-catenin signaling have a pivotal role in BBB development. To test this hypothesis, primary ECs isolated from murine cultured brain microvasculature (cBECs) were exposed for 5 days to the morphogen Wnt3a, a trigger of the canonical Wnt pathway. Differential gene expression analysis identified *Gpr126* as one of the most upregulated genes in comparison with control cells (4.8-fold, *P* < 0.05) among several known Wnt/β-catenin targets, such as axis inhibition protein 2 (*Axin2*), adenomatosis polyposis downregulated 1 (*Apcdd1*), solute carrier organic anion transporter family member 1C1 (*Slco1c1*), forkhead box F2 (*Foxf2*), forkhead box Q1 (*Foxq1*), zic family member 3 (*Zic3*), and plasmalemma vesicle–associated protein (*Plvap*) (16–19, 23, 38) (Figure 1A). Real-time quantitative PCR (qPCR) confirms that Wnt3a stimulation significantly increases known Wnt target genes, such as *Axin2*, in both cBECs and primary cultured lung ECs (cLECs). However, *Gpr126* induction is restricted to cBECs, with no effect on LECs, despite their high GPR126 expression (39). These data suggest an organ-specific effect of Wnt stimulation on *Gpr126* expression (Figure 1B). Consistent with this finding, the inhibition of Wnt/β-catenin using a tankyrase inhibitor (IWR-1) (40) or a selective inhibitor of Wnt/β-catenin signaling (MSAB, which promotes β-catenin degradation; ref. 41) abolished the induction of both *Axin2* and *Gpr126* in cBECs when exposed to Wnt3a (Supplemental Figure 1A; supplemental material available online with this article; https://doi.org/10.1172/JCI165368DS1). Notably, in contrast to the canonical Wnt pathway induced by Wnt3a, we found that the activation of non-canonical Wnt signaling with purified Wnt5a did not increase *Gpr126* mRNA in cBECs, although the expression of the selected Wnt5a target gene *Stat2* was decreased as expected (42) (Supplemental Figure 1B). Finally, a reduction in both *Axin2* and *Gpr126* was observed in vivo by testing of freshly isolated brain endothelial cells (fBECs) from mice with inducible EC-specific dominant-negative TCF4, after tamoxifen treatment (Figure 1C). However, it is worth noting that the brain’s most abundant Wnt ligands are Wnt7a and Wnt7b (43), rather than Wnt3a, which is commonly used for in vitro assays. On the other hand, the poor solubility of Wnt molecules (44) limits assays to a few ligands like Wnt3a and Wnt5a. Consistently, we found that supplementing the culture medium with Wnt7a or Wnt7b was largely ineffective in activating Wnt/β-catenin signaling in cBECs, with any induction of *Axin2* (45) (Supplemental Figure 1C).

To address these limitations, we used a coculture approach, combining immortalized brain ECs (iBECs) with neonatal astrocytes, known to be a major source of Wnt7a and Wnt7b in the brain (46). After isolating pure cocultured iBECs (*Pecam1*^hi^, *Cdh5*^hi^, *Aqp4*^lo^) (Supplemental Figure 1D), we observed significantly increased *Axin2* and *Gpr126* expression in comparison with iBECs cultured alone (Figure 1D). Notably, when astrocytes were pretreated with Wnt-C59, a potent inhibitor of porcupine responsible for Wnt palmitoylation and secretion (47), the induction of both *Axin2* and *Gpr126* in cocultured iBECs was abolished, suggesting the involvement of Wnt/β-catenin signaling (Figure 1D).

Finally, we cultured iBECs on plates precoated with gelatin-embedded purified Wnt3a, Wnt7a, and Wnt7b to specifically test their effects. *Gpr126* mRNA significantly increased in Wnt3a-, Wnt7a-, and Wnt7b-stimulated iBECs, with *Axin2* as a positive control (Supplemental Figure 1E). Treatment with IWR-1 prevented the increase in transcript levels of both *Gpr126* and *Axin2* induced by Wnt ligands (Supplemental Figure 1E).

Overall, these data identified GPR126 as a crucial target of canonical Wnt signaling in the context of the brain vasculature.

Given that Wnt signaling regulates BBB development, we speculated that GPR126 plays a critical role in brain endothelium specification (17). To investigate GPR126’s involvement in BBB establishment, we compared *Gpr126* expression in fBECs from mice at different embryonic (E11–E16), postnatal (P2–P30), and adult (P90) stages.

The purities of the fBEC preparations were tested for specific markers by real-time qPCR. fBECs showed strong enrichment of the EC-specific marker *Pecam1*, with low to barely detectable contamination of other cell types of the NVU, such as pericytes (*Cspg4*), smooth muscle cells (*Acta2*), astrocytes (*Aqp4*), lymphatic ECs (*Lyve1*, *Prox1*, *Flt4*), oligodendrocytes (*Sox10*), neurons (*Tubb3*), and immune cells (*CD68*, *Cx3cr1*, *S100a8*) (Supplemental Figure 2, A and B). *Gpr126* transcript levels in the fBECs were low in the embryo (from E11 to E16), and progressively increased in early postnatal life (from P2 to P18), during functional BBB formation. Conversely, *Gpr126* transcript levels decreased in juvenile stage (P24 to P30) and were highly downregulated in the adult brain (P90) when the BBB was fully established (Figure 1E) as confirmed by *Cldn5* upregulation (specific marker of BBB TJs; refs. 48, 49) and *Plvap* downregulation (component of endothelial fenestrae and caveolae; ref. 50) (Supplemental Figure 2, C and D). Interestingly, *Gpr126* mRNA levels from E11 to P90 significantly correlated with *Axin2* and *Ctnnb1* expression, suggesting that *Gpr126* expression is triggered by Wnt/β-catenin signaling in brain ECs (Supplemental Figure 2, E and F). Furthermore, this dynamic expression pattern was confirmed by measurement of GPR126 protein levels in fBECs at various postnatal stages (Figure 1, F and G).

Finally, we analyzed *Gpr126* receptor localization in brain microvasculature using RNAscope in situ hybridization. This confirmed widespread *Gpr126* expression in blood vessels across various regions of the brain (including the cortex, olfactory bulb, striatum, hypothalamus, cerebellum, pons, midbrain, and medulla) at P18 (Figure 1, H and I, and Supplemental Figure 2G). This observation aligned with peak physiological GPR126 expression, which is temporally synchronized with the establishment of the BBB as shown in Figure 1, E–G. At the same time point, electron microscopy revealed GPR126 on EC and pericyte plasma membranes (Figure 1J), in early endosomes near the membrane, and in late endosomes and tubular endosomal networks within the perinuclear zone of ECs (Figure 1J).

Thus, we demonstrated that GPR126 is specifically regulated by the canonical Wnt pathway in the brain, exhibiting a dynamic expression pattern that correlates with BBB development.

### GPR126 is required for correct brain vasculature development and BBB function.

**Figure 2:**
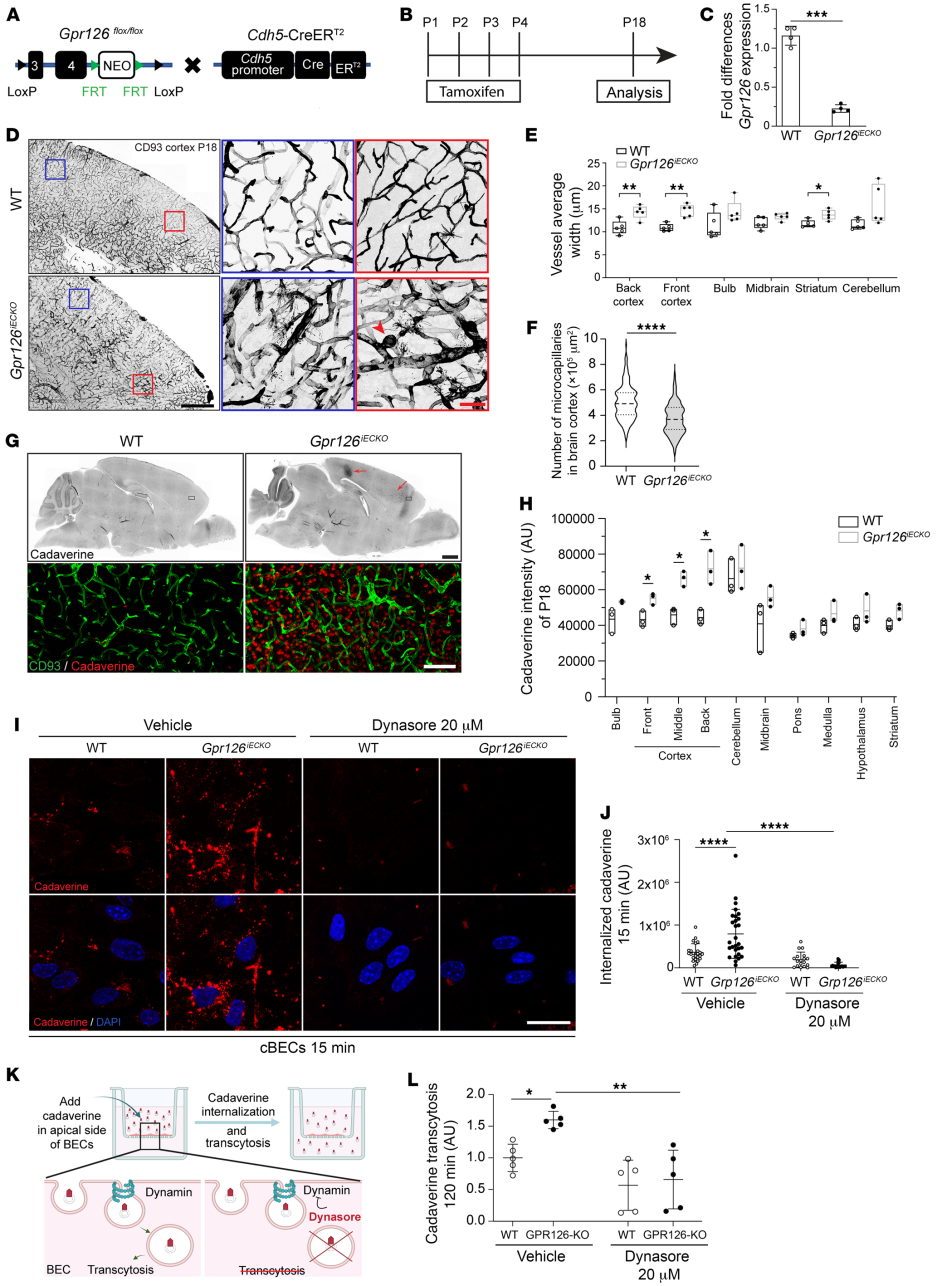
GPR126 is required for proper brain vasculature formation and function. (**A**) Mice with *Gpr126* exons 3 and 4 floxed with *loxP* sequences, containing a neomycin (NEO) cassette flanked by FRT sites (*Gpr126^fl/fl^*), were crossed with mice expressing CreER^T2^ under the *Cdh5* promoter (*Cdh5-*CreER^T2^). (**B**) Tamoxifen-inducible Cre recombination (P1–P4) analyzed at P18. (**C**) Real-time qPCR of *Gpr126* expression in BECs from tamoxifen-treated mice at P18 (*n* = 4 WT, *n* = 4 *Gpr126^iECKO^*). (**D**) Confocal images of CD93 in brain sections from WT and *Gpr126^iECKO^* mice at P18. Scale bar: 500 μm. Red and blue boxes: Magnified cortex regions. Red arrowhead, *Gpr126^iECKO^* mouse cortex vasculature tuft malformation. Scale bar: 50 μm. (**E**) Mean vessel width quantified in brain regions of mice shown in **D** (*n* = 5 WT, *n* = 5 *Gpr126^iECKO^* mice). (**F**) Microcapillary quantification per area in cortex of mice shown in **D** (*n* = 5 WT, *n* = 5 *Gpr126^iECKO^*). (**G**) Confocal images of cadaverine leakage in brain sections from WT and *Gpr126^iECKO^* mice at P18. Red arrows, leakage areas in the cortex. Scale bar: 1 mm. Bottom: Magnified cortex sections stained for CD93 (green) and cadaverine (red). Scale bar: 100 μm. (**H**) Cadaverine leakage fluorescence intensity (AU) in brain regions of mice shown in **G** (*n* = 3 WT, *n* = 3 *Gpr126^iECKO^*). (**I**) Confocal images of WT and *Gpr126^iECKO^* cBECs treated with Dynasore or vehicle. Red, cadaverine. Scale bar: 30 μm. (**J**) Internalized cadaverine fluorescence intensity (AU) in WT and *Gpr126^iECKO^* cBECs, as shown in **I** (*n* = 9 WT, *n* = 6 *Gpr126^iECKO^*). (**K**) Schematic illustration of Transwell assay evaluating cadaverine transcytosis across iBECs monolayer. (**L**) Cadaverine transcytosis quantification in WT and *Gpr126^iECKO^* iBECs, treated with Dynasore or vehicle (*n* = 5 WT, *n* = 5 *Gpr126^iECKO^*). Data are shown as means ± SD. (**C**, **E**, **F**, and **H**) Unpaired *t* tests with Welch’s correction; (**E**) bulb data, Mann-Whitney tests. (**J** and **L**) One-way ANOVA with Šidák’s multiple-comparison test, single pooled variance. *P < 0.05; **P < 0.01; ***P < 0.001; ****P < 0.0001.

To study whether the temporal correlation between GPR126 expression and BBB formation has a functional role, we generated an inducible EC-specific *Gpr126*-knockout mouse strain (*Gpr126^iECKO^*) by crossing *Gpr126^fl/fl^* mice with a *Cdh5(PAC)-*CreER^T2^ mouse strain (51) (Figure 2A). This approach bypasses the embryonic lethality of constitutive *Gpr126* deletion due to cardiac defects (52) and induces its deletion postnatally through tamoxifen treatment.

*Gpr126* deletion in mouse ECs was induced from P1 to P4, and then we analyzed both the recombination efficiency of *Gpr126* and the vascular phenotype at P18 (Figure 2B). *Gpr126* expression in fBECs from *Gpr126^iECKO^* mice was reduced by 80% compared with WT by real-time qPCR (Figure 2C). Inactivation of *Gpr126* led to multiple vasculature abnormalities in cortex and striatum, including a sparser network of enlarged vessels and frequent angiogenic sprouts (Figure 2, D–F). Moreover, the ultrastructural analysis of the brain vasculature in *Gpr126^iECKO^* mice confirmed an abnormal vessel structure characterized by increased capillary diameters (Supplemental Figure 3, A–E), while the thickness of the ECs decreased in both peripheral (Supplemental Figure 3D) and nuclear zones (Supplemental Figure 2E). Consistently, the retina of *Gpr126^iECKO^* mice at P18 displayed similar vascular changes, including enlarged arteries and veins, multiple neovascular tufts, and an overall reduction in retinal vascular coverage in comparison with control mice (Supplemental Figure 3, F–I).

To investigate whether absence of GPR126 affects BBB properties via aberrant vascular morphogenesis, we analyzed PLVAP expression, typically seen in immature BBB vasculature during development (53). PLVAP protein and mRNA were significantly increased in the cortex vasculature of *Gpr126^iECKO^* mice compared with WT littermates, suggesting that GPR126 is involved in the regulation of BBB maturation and development (Supplemental Figure 3, J and K). Consistently, we observed a significant accumulation of the low–molecular weight tracer cadaverine in the enlarged cortical vessels of *Gpr126^iECKO^* mice, indicating compromised function (Figure 2, G and H).

To address how GPR126 loss impacts the integrity of the BBB with the consequent vascular leakage, we focused on the 2 major routes that regulate vascular permeability: the paracellular route, directed by a complex of endothelial junctional molecules, and the transcellular route, regulated by different transcytosis pathways. To study whether changes in GPR126 expression alter the junction assembly and function, potentially affecting paracellular permeability, we analyzed the gene expression levels of the components of TJs (F11 receptor [*F11r*], *Cldn5*, tight junction protein 1 [*Tjp1*], occludin [*Ocln*]) and adherens junctions (junction plakoglobin [*Jup*], cadherin-5 [*Cdh5*], *Pecam1*) in fBECs. The mRNA levels of *Cldn5*, *F11r*, and *Cdh5* were significantly upregulated in fBECs from *Gpr126^iECKO^* mice compared with WT, whereas *Tjp1*, *Ocln*, and *Jup* mRNA levels were not affected (Supplemental Figure 3L). Immunofluorescence confirmed increased claudin-5 and JAM-A protein expression in the cortical vasculature of *Gpr126^iECKO^* mice compared with WT (Supplemental Figure 3, M–O). Therefore, the brain vasculature of *Gpr126^iECKO^* mice showed increased or similar junctional protein expression compared with controls, suggesting that paracellular permeability is not responsible for the tracer accumulation observed.

Then, we focused on the transcellular mechanisms of BBB permeability. We initially checked the major molecular players of the caveolae-mediated transcytosis, which is crucial in maintaining the permeability features of the BBB. Specifically, major facilitator super family domain containing 2a (MFSD2A) is constitutively expressed in the CNS ECs and suppresses caveolae-mediated transcytosis in brain ECs to ensure BBB integrity (54, 55).

However, the mRNA expression levels of both *Mfsd2a* and caveolin-1 (*Cav1*) in fBECs from *Gpr126^iECKO^* were comparable to those in control mice (Supplemental Figure 4A). On the other hand, transcellular permeability is regulated by multiple pathways beyond caveolin. Therefore, for an overall assessment of the impact of GPR126 on transcellular permeability, we conducted functional assays specifically designed to study both endocytosis and transcytosis.

We initially performed an endocytic assay with cadaverine. We observed increased internalization of cadaverine in the absence of GPR126 in cBECs (Figure 2, I and J), as well as in their corresponding immortalized cells (GPR126-KO) (Supplemental Figure 4, B and C). Notably, incubation on ice (Supplemental Figure 4D), or treatment with Dynasore, an inhibitor of dynamin, a GTPase involved in multiple endocytic mechanisms (56), inhibited cadaverine endocytosis in ECs isolated from both WT and *Gpr126^iECKO^* mice, confirming that GPR126 loss increased the endocytic uptake of cadaverine in BECs (Figure 2, I and J; see also Supplemental Figure 4, E and F, for positive control of Dynasore treatment).

Then, to understand whether this increased endocytosis leads to enhanced permeability of the endothelial layer via a transcellular route, we performed a Transwell transcytosis assay on a monolayer of WT and GPR126-KO ECs (Figure 2K). We observed an increase in cadaverine transcytosis through the EC monolayer, as indicated by the elevated fluorescence intensity of the tracer in the lower chamber of the Transwell in the absence of GPR126 in comparison with WT cells. Notably, Dynasore treatment rescued cadaverine transcytosis in GPR126-KO to levels comparable to those in WT ECs, indicating that the permeability induced by GPR126 inactivation is mediated through an enhanced activity of the transcellular route (Figure 2L and Supplemental Figure 4G).

Together, these findings indicate that the temporal synchronization of GPR126 expression and BBB formation plays a crucial role in establishing the distinctive permeability characteristics of the BBB, mostly by regulating the endocytic trafficking across the endothelial cytoplasm.

### GPR126 orchestrates BM protein deposition and ensures vascular pericyte coverage.

In addition to BECs, the BBB also involves other cellular players, including perivascular pericytes, as well as extracellular matrix (ECM) components. All these elements play a crucial role in the establishment, integrity, and functioning of the BBB.

**Figure 3:**
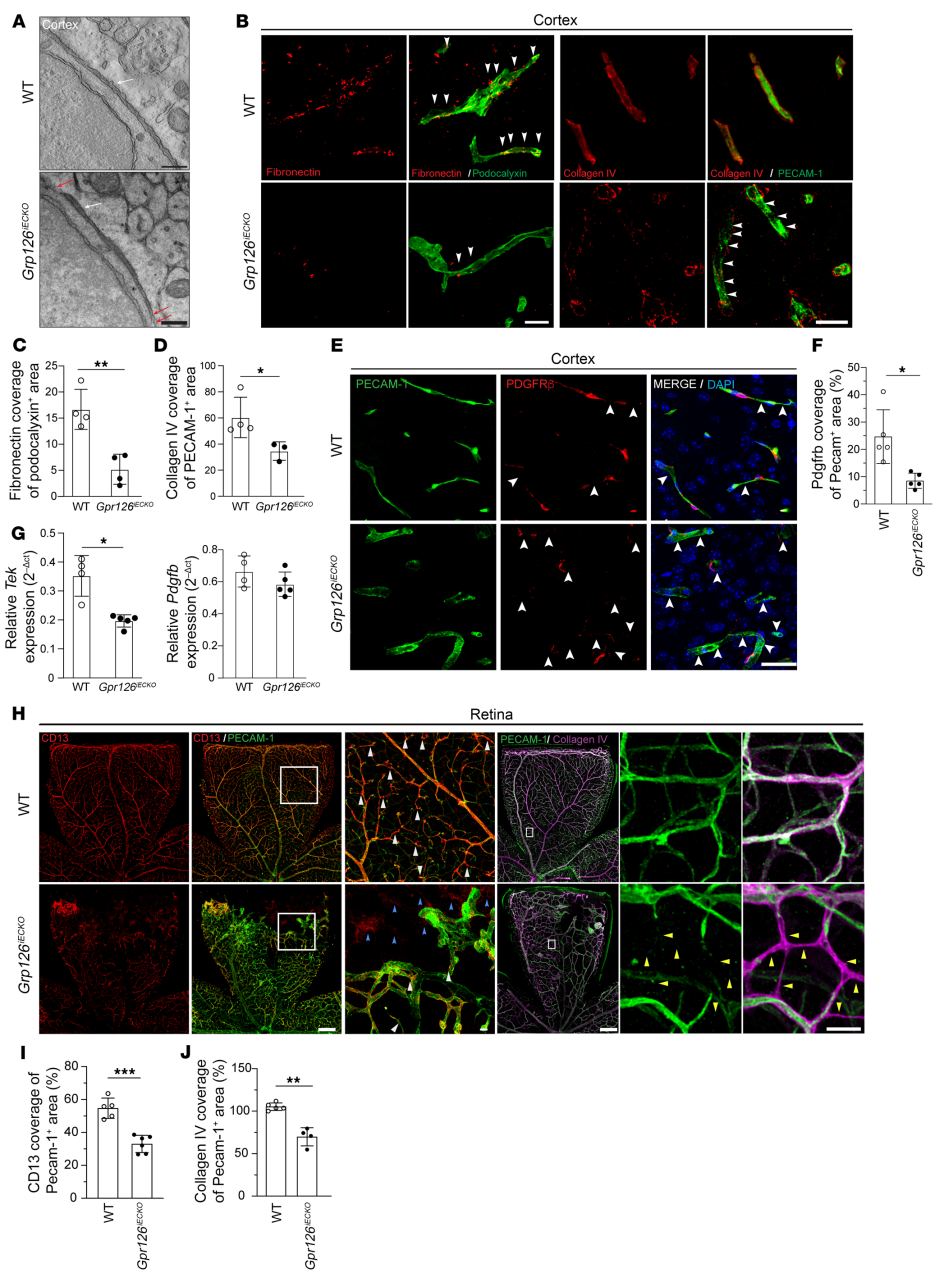
GPR126 orchestrates BM protein deposition and ensures vascular pericyte coverage. (**A**) Tomography slices of brain capillary longitudinal sections from WT and *Gpr126^iECKO^* mice at P18. White arrows, regular BM thickness; red arrows, interrupted or thin BM. See Supplemental Figure 5A for full image. Scale bars: 350 nm. (**B**) Confocal images of brain cortex cryosections from WT and *Gpr126^iECKO^* mice at P18. Vessels stained with podocalyxin or PECAM-1 (green), and BM with fibronectin or collagen IV (red). Arrowheads, protein colocalization and discontinuous collagen IV staining. See Supplemental Figure 5B for full image. Scale bars: 100 μm. (**C** and **D**) Quantification of fibronectin and collagen IV coverage in podocalyxin-positive and PECAM-1–positive areas, shown in **B** (*n* = 4 WT, *n* = 3–4 *Gpr126^iECKO^* mice). (**E**) Confocal images of brain cortex cryosections from WT and *Gpr126^iECKO^* mice at P18 show PECAM-1 (green, ECs) and PDGFR-β (red, pericytes). Arrowheads, discontinuous or absent PDGFR-β staining. Scale bar: 200 μm. (**F**) Quantification of PDGFR-β coverage in PECAM-1–positive areas, shown in **E** (*n* = 5 WT, *n* = 5 *Gpr126^iECKO^* mice). (**G**) Real-time qPCR of *tek* and *Pdgfrb* expression in fBECs (*n* = 4 WT, *n* = 5 *Gpr126^iECKO^* mice). (**H**) Confocal images of PECAM-1 (green, ECs), CD13 (red, pericytes), and collagen IV (magenta, BM) in WT and *Gpr126^iECKO^* retinas at P18. See Supplemental Figure 6A for the same field with different staining. Scale bars: 500 μm. Magnified insets show pericyte bodies, CD13 staining, and collagen IV structures without PECAM-1. Scale bars: 50 μm. (**I**) Quantification of CD13 coverage in PECAM-1–positive areas from images in **H** (*n* = 5 WT, *n* = 6 *Gpr126^iECKO^* retinas). (**J**) Quantification of collagen IV coverage in PECAM-1–positive areas, shown in **H** (*n* = 5 WT, *n* = 4 G*pr126^iECKO^* mice). Data are shown as means ± SD, unpaired *t* tests with Welch’s correction. *P <0.05; **P <0.01; ***P <0.001.

Brain ECs are embedded in the BM with pericytes, and both cell types contribute to the secretion of components like collagen IV and fibronectin, laminin-211 (α2β1γ1), laminin-511 (α5β1γ1), and laminin-411 (α4β1γ1) (57). As an adhesion GPCR, GPR126 has an important role during development via many processes, primarily through cell-cell and cell-ECM interactions, by binding to collagen IV or laminin-211 (28, 29, 34). Thus, we analyzed the structure and composition of the vascular BM. At the ultrastructural level, the cortical capillaries and the retina BM of *Gpr126^iECKO^* mice showed irregular thickness, characterized by zones without protein matrix, in comparison with WT mice, where the BM appeared as a continuous layer (Figure 3A and Supplemental Figure 5A). Then, we investigated the expression of the BM components fibronectin, collagen IV, and laminin α2 in the cortical capillaries by immunofluorescence (Figure 3B). Deposition of fibronectin around the podocalyxin-positive blood vessels of *Gpr126^iECKO^* mice was significantly reduced throughout the cortex (Figure 3, B and C, and Supplemental Figure 5B). Collagen IV showed integral and continuous structures around the PECAM-1–positive microvessels in the WT cortex that were lost in *Gpr126^iECKO^* mice, in which collagen IV deposition was discontinuous, irregular, and significantly different from WT (Figure 3, B and D). Immunostaining quantification showed a 66% decrease in collagen IV endothelial surface coverage without GPR126, compared with WT (Figure 3D). Moreover, collagen IV and laminin α2 coexpression in the BM of vascular structures was significantly reduced by 45% in the microvasculature of *Gpr126^iECKO^* mice, compared with WT (Supplemental Figure 5, C and D). The irregular distribution of collagen IV and laminin α2 was also confirmed in cBECs, suggesting that the loss of GPR126 in ECs specifically mediates the secretion and aberrant deposition of laminin α2 (Supplemental Figure 5E). To address whether altered fibronectin and collagen IV deposition is related to reduced expression or different matrix dynamic of *Fn1*, *Col4a1*, and *Col4a2*, we performed real-time qPCR in fBECs of WT and *Gpr126^iECKO^* pups at P18. We found a slight increase in *Col4a1* and *Col4a2* expression and similar *Fn1* expression in *Gpr126^iECKO^* compared with control mice (Supplemental Figure 5, F–H), suggesting that the reduced collagen IV around the vasculature was due to impaired protein deposition, not decreased mRNA expression in ECs. Consistently, increased transcript levels of metalloproteases *Mmp3*, *Mmp9*, and *Mmp14* were observed in ECs of *Gpr126^iECKO^* mice compared with WT (Supplemental Figure 5, I–K), potentially causing BBB disruption through ECM degradation.

Next, for an overall analysis of the BBB development, we shifted our focus from ECs and BM components to perivascular cells. Immunostaining of brain microvasculature for PDGFR-β (a pericyte marker) and PECAM-1 (an EC marker) showed a 60% decrease in pericyte coverage in *Gpr126^iECKO^* compared with WT (Figure 3, E and F). Then, we investigated the role of GPR126 in pericyte recruitment around vascular ECs through known pathways such as PDGFβ/PDGFR-β and angiopoietin-1 (ANG1)/TIE2 (58). Though there was no significant impact on *Pdgfb* transcript levels in ECs lacking GPR126, the expression of *tek*, the endothelial receptor for ANG1, was reduced in *Gpr126^iECKO^* mice compared with WT (Figure 3G). Since ANG1-TIE2 interaction is crucial for pericyte recruitment to the vessel wall (59), reduced *tek* expression may explain the decreased pericyte coverage in *Gpr126^iECKO^* brain vasculature. Similar data were obtained for the retina. In control mice, retinal blood vessels were positive for the pericyte marker CD13, while *Gpr126^iECKO^* mice showed abnormal vasculature characterized by a reduced, irregular, and disorganized distribution of pericytes (Figure 3, H and I). Consistently, α-smooth muscle actin (αSMA) was significantly reduced in *Gpr126^iECKO^* mice (Supplemental Figure 6, A and B). Moreover, the presence of CD13-positive and PECAM-1–negative structures in the front of the retina of *Gpr126^iECKO^* mice at P18 implied a regression of the endothelial layers of the growing vessels (Figure 3H). This process was also confirmed by the appearance of numerous collagen IV–positive and PECAM-1–negative stained structures that represented empty matrix sleeves in the *Gpr126^iECKO^* vasculature and decorated blind-ended vessels (Figure 3, H and J).

Collectively, these findings indicate that GPR126 is essential for correct deposition of the BM and pericyte recruitment during vessel growth.

### GPR126 induces angiogenesis in the CNS.

**Figure 4:**
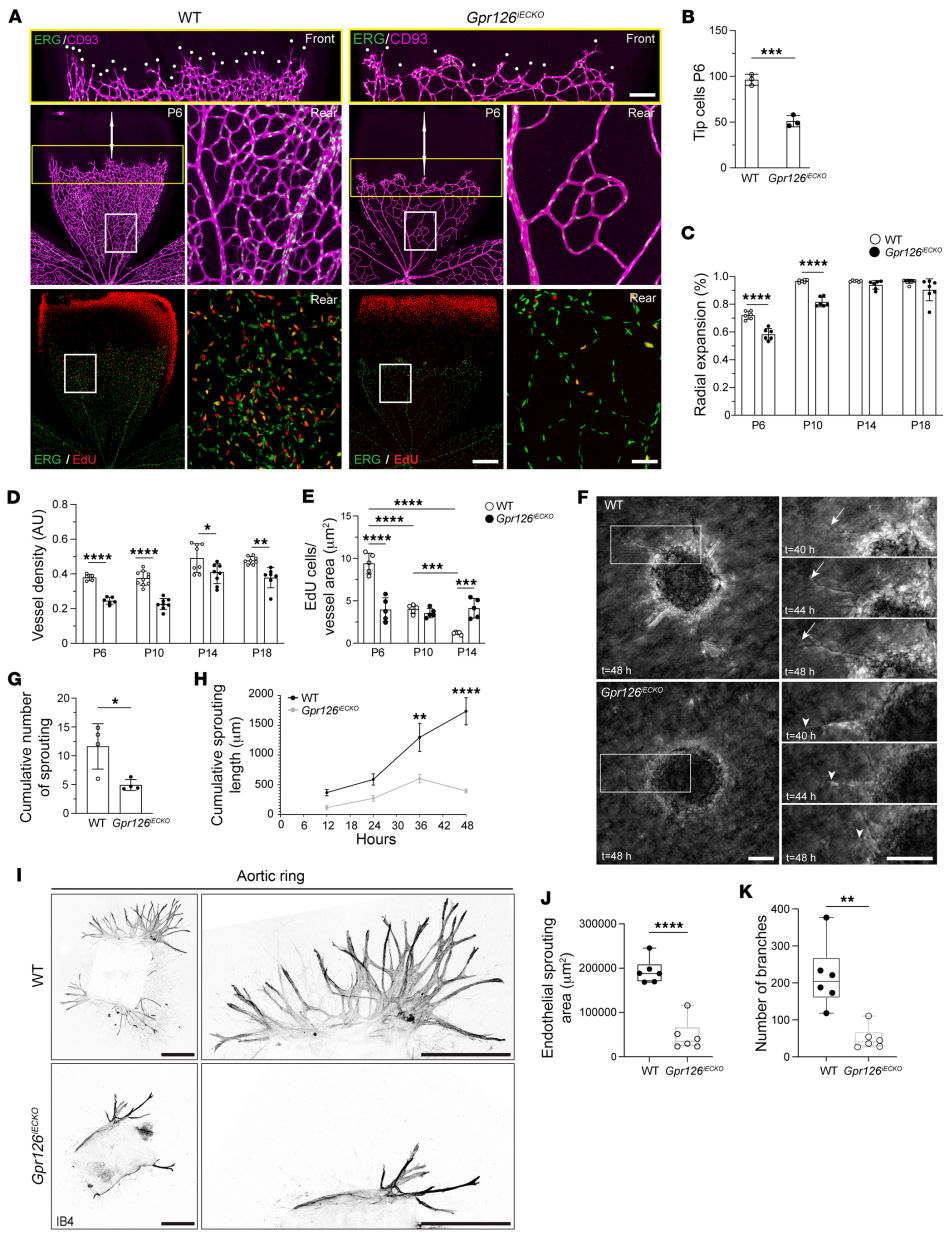
GPR126 is required for angiogenesis in the retina and in the brain. (**A**) Confocal images of CD93 (magenta, ECs), ERG (green, EC nuclei), and EdU (red, proliferation) in WT and *Gpr126^iECKO^* mouse retinas at P6 show retinal vessels at the petal, front, and rear regions. White dots highlight tip cells. Scale bars: petal, 500 μm; rear, 100 μm; front, 250 μm. (**B**) Tip cell quantification in the yellow rectangle in **A** (*n* = 3 WT, *n* = 3 *Gpr126^iECKO^* retinas). (**C**) Retinal vasculature radial expansion at P6, P10, P14, and P18 in WT and *Gpr126^iECKO^* mice (*n* = 6–8 WT, *n* = 6–7 *Gpr126^iECKO^* retinas). (**D**) Postnatal retinal vessel density (P6, P10, P14, P18) in WT and *Gpr126^iECKO^* mice (*n* = 5–10 WT, *n* = 6–9 *Gpr126^iECKO^* retinas). (**E**) EdU-positive ECs/μm^2^ of vessel area postnatally (P6, P10, P14, P14) in WT and *Gpr126^iECKO^* retinas (*n* = 5 WT, *n* = 5 *Gpr126^iECKO^* retinas). (**F**) Phase-contrast images of sprouting spheroids from fBECs of WT and *Gpr126^iECKO^* mice at P18 after stimulation with VEGF (80 ng/mL) and FGFb (50 ng/mL). Right: Magnified images at *t* = 40, 44, and 48 hours (for time-lapse, see Supplemental Video 1). Arrows, sprouting ECs; arrowheads, retracting ECs. Scale bars: 40 μm. (**G**) Cumulative sprouts per spheroid of WT (*n* = 16) and *Gpr126^iECKO^* (*n* = 16) fBECs. Each symbol represents an experiment (*n* = 4 WT, *n* = 4 *Gpr126^iECKO^* mice). (**H**) Cumulative sprouting lengths per spheroid of WT (*n* = 12) and *Gpr126^iECKO^* (*n* = 12) fBECs after 12, 24, 36, and 48 hours (*n* = 6 WT, *n* = 6 *Gpr126^iECKO^* mice). (**I**) Aortic rings from WT and *Gpr126^iECKO^* mice show vascular sprouts (magnified) via IB4 immunostaining. Scale bars: 500 μm. (**J** and **K**) Endothelial sprouting area (**J**) and branch numbers (**K**) as depicted in **I** (*n* = 6 WT, *n* = 6 *Gpr126^iECKO^* mice). Data are shown as means ± SD. (**B**–**D**, **G**, **J**, and **K**) Unpaired *t* tests with Welch’s correction; (**E** and **H**) 2-way ANOVA. *P < 0.05; **P < 0.01; ***P < 0.001; ****P < 0.0001.

Brain angiogenesis is very tightly coupled to the formation of the BBB (60). Thus, we investigated the role of GPR126 in developmental retinal angiogenesis after birth. At P6, the growing superficial vessel plexus had not reached the retina’s periphery, and numerous sprouts were visible at the angiogenic growth front. Tamoxifen-induced *Gpr126* depletion in ECs at P1 significantly reduced tip cell numbers, radial expansion, and vascular density, indicating GPR126’s importance in retina angiogenesis (Figure 4, A–D).

At P14, the extension of the superficial vascular network was complete in both WT and *Gpr126^iECKO^* mice (Figure 4C and Supplemental Figure 7A). However, the deep plexus network formation was still significantly delayed in the *Gpr126^iECKO^* retina (Supplemental Figure 7, A and B), which suggested that endothelial GPR126 deletion impaired the ECs’ migratory performance. Moreover, at P14, vessel density remained significantly reduced without GPR126 compared with WT, and veins enlarged (Figure 4D and Supplemental Figure 7, A and B).

Ethynyl deoxyuridine (EdU) incorporation, indicating endothelial proliferation, was observed in WT ERG-positive EC nuclei at P6. However, this significantly decreased by P14, with EdU-labeled ECs primarily found in the most distal, peripheral sections of veins (Figure 4, A and E, and Supplemental Figure 7C). Conversely, EdU and ERG double-positive cells in the *Gpr126^iECKO^* retina were significantly reduced at P6 compared with WT, with similar numbers observed at P14 (Figure 4, A and E, and Supplemental Figure 7C). It is noteworthy that at P14, ERG-positive EC nuclei were significantly increased in the *Gpr126^iECKO^* retina compared with WT, indicating that absence of GPR126 delayed vasculature development (Figure 4E and Supplemental Figure 7C).

To investigate the involvement of GPR126 in brain angiogenesis, we performed ex vivo experiments using cBECs from WT and *Gpr126^iECKO^* mice at P18. Cell proliferation and migration are intricately linked to angiogenesis; hence, we explored the role of GPR126 in both processes. Proliferation was measured using bromodeoxyuridine incorporation and Ki67 staining. In cBECs from *Gpr126^iECKO^* mice, proliferation was reduced under standard culture conditions compared with WT (Supplemental Figure 7, D–F). Similarly, cBECs from *Gpr126^iECKO^* mice showed significantly slower cell migration compared with WT, as evidenced by the closing of the wound in a scratch wound healing assay (Supplemental Figure 7, G and H).

Finally, to determine how GPR126 regulates EC movement during angiogenesis, spheroids composed of fBECs from WT and *Gpr126^iECKO^* mice were placed in a 3D collagen matrix to monitor VEGF-FGFβ–induced angiogenesis. Depletion of GPR126 significantly decreased sprout formation and elongation from spheroids (Figure 4, F–H), confirming its critical role in angiogenesis. Moreover, in live-cell time-lapse, sprouting length increased over time in WT ECs (Figure 4, F–H, and Supplemental Video 1), whereas absence of GPR126 resulted in decreased sprout length, with regression events occurring after 2 days of growth (Figure 4, F and H). This vessel regression phenotype was also observed in the retina at P18. Indeed, the absence of GPR126 significantly decreased vascular density, with a trend toward reduced radial expansion, indicating defective vascular maturation and consequent vessel regression (Figure 3, H–J, and Figure 4, C and D).

To further confirm defective angiogenesis, we investigated the impact of GPR126 depletion using an aortic ring assay. Angiogenic sprouting was observed from aortic rings in both control and *Gpr126^iECKO^* mice. However, the endothelial sprouting area and the number of branches were diminished in *Gpr126^iECKO^* mice, indicating that GPR126 supports sprouting angiogenesis (Figure 4, I–K).

Collectively, these data show that impaired proliferation and migration in GPR126-deficient ECs cause angiogenesis defects in the CNS of *Gpr126^iECKO^* mice.

### GPR126 modulates the expression levels of LRP1.

To mechanistically clarify the role of GPR126 during BBB formation, we profiled the brain endothelial transcriptional changes upon GPR126 depletion at P18. Differential expression analyses revealed significant transcriptional rewiring in *Gpr126^iECKO^* compared with WT (Supplemental Figure 8, A and B).

**Figure 5:**
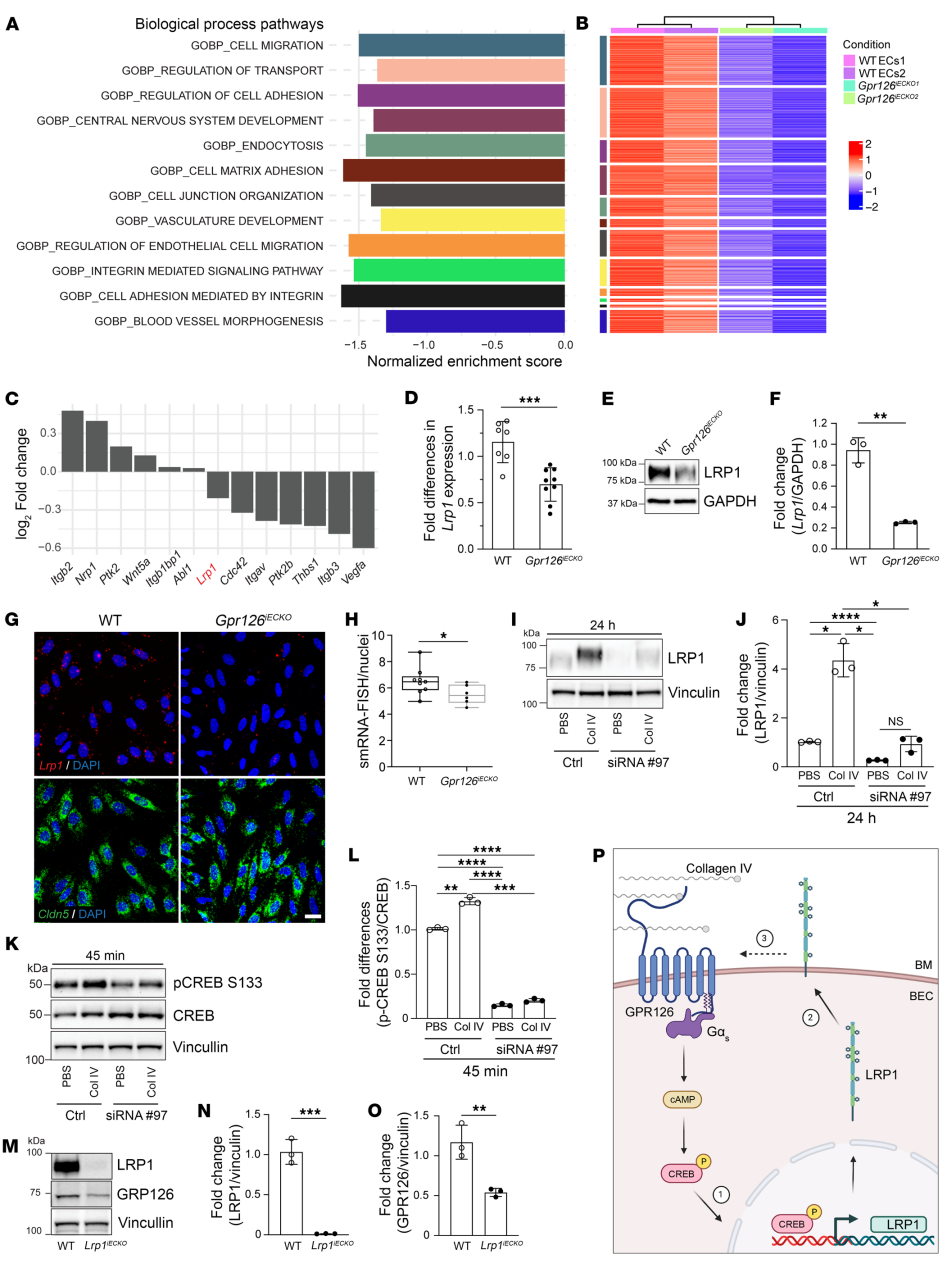
GPR126 modulates LRP1 expression levels. (**A**–**C**) RNA sequencing of fBECs from WT and *Gpr126^iECKO^* mice at P18. (**A**) Normalized enrichment scores from GSEA for differentially expressed genes in *Gpr126^iECKO^* (*P* ≤ 0.01, adjusted *P* ≤ 0.05, at least 30 altered genes). (**B**) Heatmap displaying *z* scores of leading-edge genes from GSEA for selected GO terms (*n* = 4 WT, *n* = 4 *Gpr126^iECKO^* mice per replicate). (**C**) Log_2_ fold change of selected genes differentially expressed in *Gpr126^iECKO^*. (**D**) Real-time qPCR of *Lrp1* in fBECs (*n* = 7 WT, *n* = 9 *Gpr126^iECKO^*). (**E** and **F**) Immunoblotting for LRP1 in fBECs from WT and *Gpr126^iECKO^* mice at P18 (**E**) normalized over GAPDH (**F**) (*n* = 3 WT, *n* = 3 *Gpr126^iECKO^*). (**G**) FISH confocal images for *Lrp1* (red) and *Cldn5* (green) mRNA in cBECs from WT and *Gpr126^iECKO^* mice at P18. Scale bar: 20 μm. (**H**) Single-molecule RNA (smRNA) of *Lrp1* per nucleus in **G**. Each symbol represents a field of 40 cells (*n* = 9 WT, *n* = 6 *Gpr126^iECKO^*). (**I** and **J**) Immunoblotting for LRP1 (**I**) normalized over vinculin (**J**) in cBECs transfected with control or *Gpr126* siRNA and treated with collagen IV or PBS (**I**) (*n* = 6 WT mice). (**K** and **L**) Immunoblotting for total CREB and phospho-CREB S133 (**K**) normalized over vinculin (**L**) in cBECs treated as in **I** (*n* = 12 WT mice). (**M**–**O**) Immunoblotting for LRP1 and GPR126 in cBECs from adult WT and *Lrp1^iECKO^* (**M**), normalized over vinculin (**N** and **O**) (*n* = 3 WT, *n* = 3 *Lrp1^iECKO^*). (**P**) GPR126 regulates LRP1 expression and vice versa. 1. Collagen IV–GPR126 interactions induce cAMP, phospho-CREB, and LRP1. 2. LRP1 localizes at the plasma membrane. 3. This supports GPR126 expression and signaling. Dashed arrow, undefined LRP1-mediated induction of GPR126. Data are shown as means ± SD. (**F**, **J**, **K**, **N**, and **O**) Each symbol represents an experiment. (**D**, **F**, **H**, **N**, and **O**) Unpaired *t* tests with Welch’s correction; (**J** and **L**) Brown-Forsythe and Welch’s ANOVA, Dunnett’s T3 multiple-comparison tests. *P < 0.05; **P < 0.01; ***P < 0.001; ****P < 0.0001.

According to gene set enrichment analysis (GSEA), loss of GPR126 resulted in global downregulation of a variety of biological processes. Thus, we focused on a subset of Gene Ontology (GO) terms related to specific BBB properties and functions (Figure 5A), with a negative enrichment score for most of these genes (Supplemental Figure 8C). Moreover, the leading-edge genes (i.e., the subset of genes that contributes the most to the signal) of these altered GO terms showed concordant downregulation among samples (Figure 5B). Finally, we selected for validation those genes that were present in at least 75% of all the selected GO terms (Figure 5C and Supplemental Table 1). Among these genes, we focused on *Lrp1*, given increasing evidence of its involvement in key EC functions like BBB transcytosis, permeability, and angiogenesis (7, 61–63).

We confirmed expression of both *Lrp1* mRNA and protein in WT ECs, significantly reduced in the absence of GPR126 in fBECs from mice at P18 (Figure 5, D–F). Consistently, in situ hybridization detected single-molecule RNA of *Lrp1* in cBECs expressing *cldn5* (endothelial marker), with a significant decrease of *Lrp1* signal observed in *Gpr126^iECKO^* cBECs (Figure 5, G and H) as well as in their corresponding immortalized cells (GPR126-KO) (Supplemental Figure 9, A and B).

We further studied LRP1 using different approaches to modulate GPR126 expression and its downstream signaling activity. Acute GPR126 downregulation in WT cBECs using small interfering RNAs (siRNAs) (Supplemental Figure 9, C–F) significantly decreased LRP1 expression at both protein and mRNA levels, in comparison with control cells (Figure 5, I and J, and Supplemental Figure 9G).

Next, we investigated whether LRP1 expression is triggered by the activation of GPR126 receptor binding to its known ligand collagen IV, which produces cyclic adenosine monophosphate (cAMP) and induces CREB phosphorylation (29). Treatment of WT cBECs with soluble collagen IV induced CREB phosphorylation at S133, while in the absence of GPR126, the levels of phospho-CREB S133 were comparable to those in vehicle-treated cells (Figure 5, K and L). Moreover, collagen IV treatment of WT ECs for 24 hours stimulated GPR126 signaling and increased LRP1 protein and mRNA levels, which was not seen upon GPR126 depletion (Figure 5, I and J, and Supplemental Figure 9G).

Finally, to further describe the connections between LRP1 and GPR126 receptors, we investigated whether the absence of LRP1 affected the expression of GPR126, using BECs isolated from recombined *Slco1c1-*CreER^T2^/*Lrp1^fl/fl^* mice (7). In the *Lrp1^iECKO^* BECs, both *Lrp1* and *Gpr126* mRNA and protein levels were downregulated, compared with WT (Figure 5, M–O, and Supplemental Figure 9, H and I).

These data (Figure 5P) demonstrate that LRP1 is a specific target of activated GPR126 when it binds to collagen IV, highlighting a transcriptional loop between LRP1 and GPR126.

### GPR126 synergizes with LRP1 and β_1_ integrin to steer EC migration during angiogenesis.

**Figure 6:**
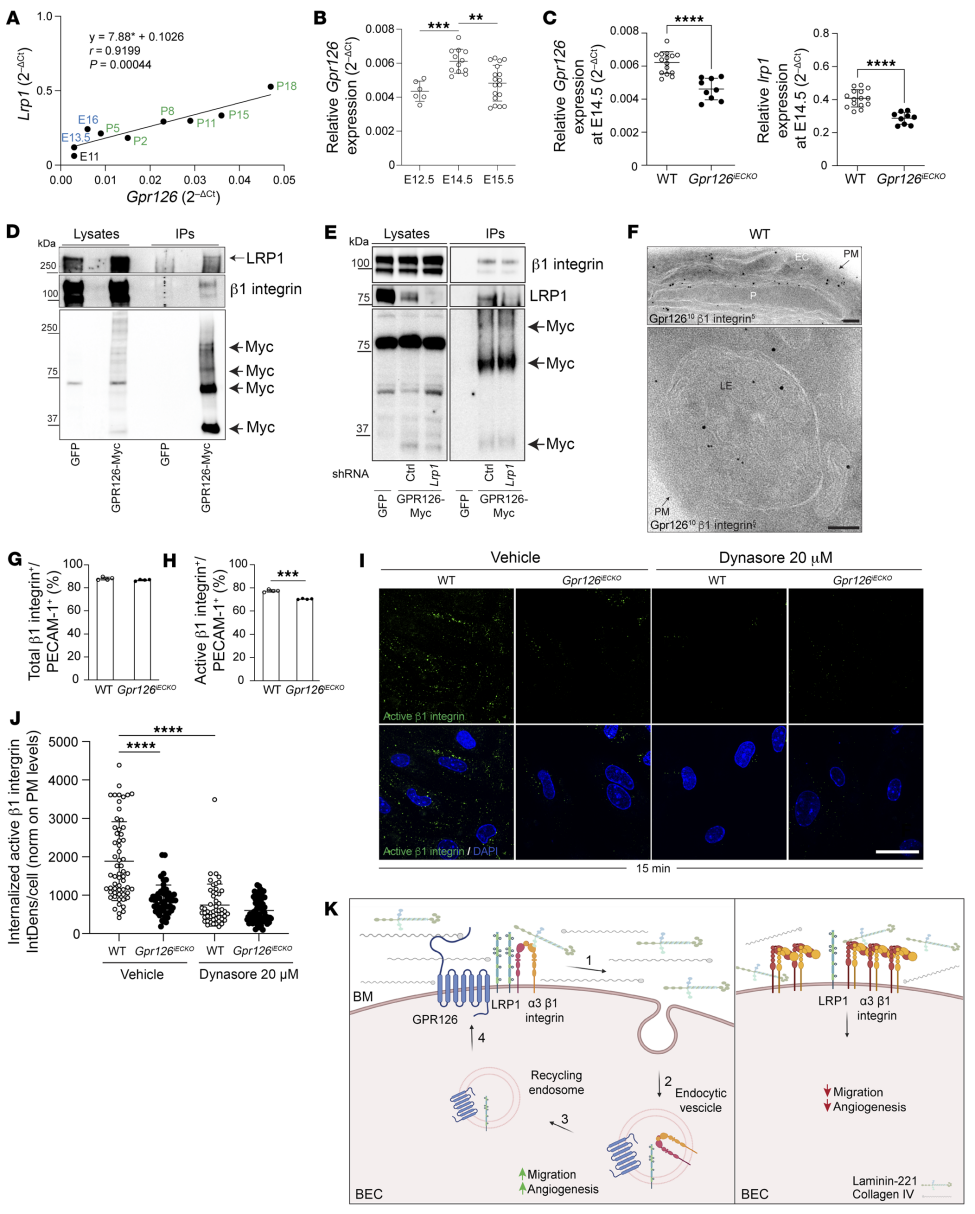
GPR126 synergizes with LRP1 and β_1_ integrin to steer EC migration during angiogenesis. (**A**) Pearson’s correlation analysis (*r*) of *Lrp1* and *Gpr126* mRNA in fBECs from WT mice at embryonic (E11–E16) and postnatal (P2–P18) stages. (**B**) Real-time qPCR of *Gpr126* in fBECs at E12.5, E14.5, and E15.5 (*n* = 6 E12.5, *n* = 12 E14.5, *n* = 18 E15.5; Brown-Forsythe and Welch’s ANOVA, Dunnett’s T3 multiple-comparison tests). (**C**) Real-time qPCR of *Gpr126* and *Lrp1* in fBECs at E14.5 (*n* = 15 WT, *n* = 9 *Gpr126^iECKO^*). (**D** and **E**) Immunoblotting for LRP1, β_1_ integrin, and Myc in iBECs transfected with GFP or GPR126-Myc (**D**) and infected with control shRNA (Ctrl) and shRNA against *Lrp1* (**E**). Lysates were immunoprecipitated (IP) with anti-rabbit-Myc agarose (**D**) or with anti-mouse-Myc agarose (**E**). GFP-transfected iBECs were used as IP controls. Data represent 3 independent experiments. (**F**) Immunogold for GPR126 (10 nm) and β_1_ integrin (5 nm) in cortical capillaries from WT mouse at P18. PM, plasma membrane; P, pericyte; LE, late endosome. Scale bars: 100 nm. (**G** and **H**) Percentage of total β_1_ integrin–positive (**G**) and active β_1_ integrin–positive (**H**) fBECs (gated as PECAM-1^+^ cells) isolated from WT and *Gpr126^iECKO^* mice at P18 and analyzed by flow cytometry (*n* = 3 WT, *n* = 3 *Gpr126^iECKO^*). (**I** and **J**) Internalized active β_1_ integrin (**I**) and single-cell fluorescence intensity (**J**) measured in Dynasore- or vehicle-treated cBECs from WT and *Gpr126^iECKO^* (*n* = 9 WT, *n* = 6 *Gpr126^iECKO^*; 1-way ANOVA, Šidák’s multiple-comparison test, assuming a single pooled variance). IntDens, integrated density. Scale bar: 30 μm. (**K**) GPR126 complex dynamic. Left: GPR126 binds LRP1 and α_3_β_1_ upon collagen IV induction (step 1). LRP1-mediated endocytosis promotes EC migration and angiogenesis (step 2). Recycling endosomes (step 3) restore LRP1 and GPR126 to the PM (step 4). Right: Absence of GPR126 decreases BM deposition and LRP1 expression. α_3_β_1_ Integrin is not internalized, reducing migration and angiogenesis. Data are shown as means ± SD. (**C**, **G**, and **H**) Unpaired *t* tests with Welch’s correction. **P < 0.01; ***P < 0.001; ****P < 0.0001.

Recent studies have linked LRP1 with endothelial function and angiogenesis (61). Indeed, LRP1 depletion in the mouse embryo leads to angiogenic defects and disruption of endothelial integrity (63). Hence, we postulated that GPR126 and LRP1 cooperate during angiogenesis. To address this hypothesis in vivo, we analyzed the mouse forebrain between E10.0–E11.5, when angiogenesis has been shown to begin, and P18, before brain vasculature becomes functionally mature and quiescent at P25 (64). Thus, we compared the dynamic expression of *Lrp1* with that of *Gpr126* in fBECs at different developmental stages between E11 and P18. We found a significant correlation between *Gpr126* and *Lrp1* mRNA levels (Pearson’s correlation coefficient, *r* = 0.91, *P* = 0.00044) (Figure 6A).

Since BBB maturation begins already at E15 (54), we then focused on earlier developmental stages, when angiogenesis is the dominant event characterizing this step of BBB formation (9). This analysis allows for a specific focus on brain angiogenesis before the complete maturation of the BBB.

First, we compared the expression of *Gpr126* in fBECs from mice at different embryonic stages: E12.5, E14.5, and E15.5. *Gpr126* transcript levels in the fBECs were low at E12.5, peaked at E14.5, and significantly decreased at E15.5 (Figure 6B). Since *Gpr126* endothelial expression peaked at E14.5, we then inactivated *Gpr126* in ECs after tamoxifen administration from E9 to E11 and obtained fBECs at E14.5. Real-time qPCR revealed a 30% reduction in *Gpr126* expression in fBECs from *Gpr126^iECKO^* mouse embryos, resulting in decreased *Lrp1* transcript levels compared with WT (Figure 6C). These data indicate concordant regulation of *Gpr126* and *Lrp1* expression during the angiogenic phase of BBB development, with GPR126 necessary to maintain this temporal synchronization.

Moreover, it is established that LRP1 acts as a potential regulator of cell migration and adhesion by controlling the endocytosis of β_1_ integrin (65), which itself plays a crucial role in angiogenesis (66). Therefore, we hypothesized that GPR126 could synergize with LRP1 and β_1_ integrin in regulating EC migration during angiogenesis.

To investigate whether GPR126 interacts with LRP1 and β_1_ integrin, a Myc-tagged GPR126 protein was stably expressed in iBECs (GPR126-Myc), leading to a 6-fold *Gpr126* overexpression and increased *Lrp1* mRNA levels, compared with GFP-overexpressing cells (Supplemental Figure 10A). Then, using immunoprecipitation assays, we found that in iBECs, GPR126-Myc coimmunoprecipitated both LRP1 and β_1_ integrin (Figure 6D), and that a β_1_ integrin–specific antibody coimmunoprecipitated with GPR126-Myc, which demonstrated the presence of both GPR126 and LRP1 in the protein complex (Supplemental Figure 10B).

Finally, to determine whether the interaction between β_1_ integrin and GPR126 requires LRP1, we initially produced GPR126-Myc cells that were stably infected with lentiviral vectors expressing shRNA targeting LRP1. We tested 4 distinct shRNA constructs to establish stable cell lines deficient in LRP1. The efficiency of the different constructs was assessed using real-time qPCR, revealing that sh#*Lrp1A* and sh#*Lrp1B* RNAs significantly reduced *Lrp1* mRNA expression by approximately 80%–90% compared with the control (Supplemental Figure 10C). Subsequently, sh#*Lrp1A* was chosen to investigate whether the downregulation of LRP1 affected the interaction between GPR126 and β_1_ integrin. In accordance with the diminished mRNA levels (Supplemental Figure 10C), the protein signals were reduced in the presence of sh#*Lrp1A* RNA compared with the control (Supplemental Figure 10, D–F). However, despite the downregulation of LRP1, GPR126 and β_1_ integrin were observed to still interact, as demonstrated by protein coimmunoprecipitation (Figure 6E), suggesting a direct interaction between GPR126 and β_1_ integrin.

The overall data further clarified the link between GPR126, LRP1, and β_1_ integrin. Specifically, the synchronized expression dynamics of GPR126 and LRP1 during BBB development led to the formation of a specific protein complex with β_1_ integrin. Notably, GPR126 and β_1_ integrin can also directly interact in the absence of LRP1 (Figure 6E).

Furthermore, at the electron microscopy level, β_1_ integrin and GPR126 were detected on the plasma membrane and in late endosomes of ECs in the brain cortex. This observation suggests that both proteins undergo internalization, trafficking to late endosomes, and recycling to the plasma membrane (Figure 6F). Additionally, it is known that integrins undergo trafficking with differing kinetics based on their activity status, influencing cell migration and invasion (67). Consequently, the active form of β_1_ integrin is mainly localized in endosomes during migration, while the inactive integrin is in detached membrane protrusions on the plasma membrane, likely involved in adhesion processes.

Given that the absence of GPR126 impaired EC migration (Figure 4, A–C, and Supplemental Figure 7, G and H), we hypothesized that this effect might be due to reduced β_1_ integrin trafficking and activation. To investigate, we analyzed surface levels of total and active β_1_ integrin in fBECs from WT and *Gpr126^iECKO^* mice at P18 using flow cytometry. While total β_1_ integrin remained similar, the active form was slightly but significantly reduced in the absence of GPR126 (Figure 6, G and H).

Moreover, to corroborate the involvement of GPR126 in β_1_ integrin trafficking, we investigated the endocytosis of active β_1_ integrin. Importantly, β_1_ integrin, in its active form, displayed reduced internalization in GPR126-depleted cells to an extent similar to that observed with Dynasore treatment (Figure 6, I and J; see also Supplemental Figure 10, G and H, for positive control of Dynasore treatment). This effect was not enhanced by Dynasore treatment in GPR126-depleted cells (Figure 6, I and J), indicating that GPR126 supports dynamin-dependent internalization of active β_1_ integrin. Notably, integrins are typically recycled back to the plasma membrane after internalization, crucial for regulating plasma membrane levels and polarized localization of active β_1_ integrin. GPR126’s role in β_1_ integrin endocytosis is expected to influence dynamic cell adhesion site rearrangement at the surface, critical for regulating cell migration. Further evidence supporting this interconnection comes from the wound healing assay in which GPR126-depleted cells, as well as Dynasore-treated cells, exhibited decreased collective motility and delayed wound closure (Supplemental Figure 10, I and J). Given the established role of LRP1 in β_1_ integrin endocytosis (65) and the reduced expression of LRP1 in *Gpr126^iECKO^* cells (Figure 5, D–H), multiple lines of evidence suggested the involvement of the GPR126-LRP1 complex in cell migration, regulating adhesiveness levels through β_1_ integrin activation and recycling. To determine the α subunit coupled to β_1_ integrin, we found that GPR126-Myc coimmunoprecipitated with α_3_β_1_ integrin but not with the α_1_ subunit (Supplemental Figure 10K).

Thus, we identified a molecular mechanism involving GPR126 and its partners in the regulation of EC migration during angiogenesis, as summarized in Figure 6K.

Altogether, our findings identify the GPR126–LRP1–α_3_β_1_ integrin complex as a pivotal control module linking Wnt signaling and angiogenesis during BBB development. This regulatory function has a broad effect, steering EC migration, BM deposition, and pericyte coverage, all cooperating in structuring both BBB morphogenesis and function.

## Discussion

The formation of the BBB requires precise coordination among various cellular players, including vascular ECs, pericytes, perivascular fibroblasts, and ECM components. During BBB development, ECs undergo an organotypic specification to acquire unique molecular and functional features. This multistep process is initiated by Wnt ligands activating a BBB-specific angiogenic program (16, 17, 19, 20). However, the exact downstream effectors of Wnt signaling in directing brain angiogenesis toward a BBB differentiation program remain unclear.

Our study identified *Gpr126* as a crucial canonical Wnt target gene that links Wnt signaling to BBB development. We found that *Gpr126* is highly upregulated in response to Wnt3a (canonical) but not Wnt5a (non-canonical) stimulation in cBECs. Both in vitro and in vivo inhibition of β-catenin signaling confirmed the specific effect of canonical Wnt signaling on endothelial *Gpr126* expression. Additionally, astrocyte-derived Wnt7a/b, the primary source of canonical Wnt ligand in the brain (46, 68), induces *Gpr126* elevation, mimicking the effect of recombinant Wnt3a. Notably, Wnt3a does not induce *Gpr126* upregulation in other GPR126-expressing ECs, such as primary lung ECs, indicating an organ-specific induction by canonical Wnt ligands in the brain.

Considering that one of the most prominent events regulated by Wnt signaling is BBB development, here we have investigated the role of GPR126 as a Wnt effector molecule linking Wnt signaling and BBB formation (16, 17, 69). In line with this initial speculation, GPR126 expression is low during embryonic stages but increases postnatally, peaking at P18, matching with BBB establishment (9), and then decreases in adulthood (39, 52). This temporal correlation suggests a potential synergistic role with other Wnt/β-catenin target genes in BBB development such as *Axin2* and *Ctnnb1*. Using an inducible EC-specific GPR126-knockout mouse (*Gpr126^iECKO^*), we bypassed embryonic lethality and assessed the role of GPR126 postnatally. *Gpr126^iECKO^* mice exhibited vascular abnormalities, including a sparser network of enlarged vessels and frequent angiogenic sprouts in both brain and retina.

Alongside this aberrant vasculature, we found a relevant impairment of BBB function with a significant cadaverine accumulation in the cerebral cortex where the vessels were most enlarged and the fenestration marker PLVAP was upregulated. However, the increased or similar expression of junctional proteins (claudin-5, JAM-A, VE-cadherin, occludin, plakoglobin, and ZO-1) in the *Gpr126^iECKO^* mice suggests that the structural components regulating the paracellular route for permeability remain intact despite the absence of GPR126. On the other hand, reduced endothelial *Vegfa* expression does not explain the barrier breakdown, as VEGF-A typically causes leakage and is produced by astrocytes affecting local ECs (68, 70). Similar VEGF-A levels in whole-brain RNA extracts from WT and *Gpr126^iECKO^* mice suggest that the increased permeability is VEGF independent. Instead, increased dynamin-dependent endocytosis and transcytosis of cadaverine indicate GPR126’s role in the BBB’s transcellular pathway. Although no changes were observed in *Mfsd2a* and *Cav1* transcript levels, future research should explore the interaction between GPR126, Wnt signaling, and lipid metabolism in brain ECs (71).

Beyond permeability, GPR126 affected BBB structure. GPR126-deficient mice showed ultrastructural changes in cortical and retinal blood vessels, with irregular BM thickness and reduced perivascular deposition of fibronectin, collagen IV, and laminin α2. However, despite no impact on BM protein transcript levels, GPR126 inhibition leads to significant dysregulation of metalloproteases, suggesting a mechanism whereby GPR126 influences BBB integrity through ECM remodeling and degradation, rather than transcriptional control. Moreover, our data showed a significant reduction in pericyte coverage in both the brain and retina of *Gpr126^iECKO^* mice, emphasizing GPR126’s role in vascular architecture. The reduced pericyte coverage suggests disrupted endothelial-pericyte communication, compromising BBB integrity. We explored the impact of GPR126 deficiency on pericyte recruitment pathways, including PDGFβ and TIE2. While *Pdgfb* transcript levels remained steady, expression of *tek* (the ANG1 receptor) decreased in *Gpr126^iECKO^* mice, potentially explaining the reduced pericyte coverage. This highlights the connection between GPR126 signaling and angiogenic pathways crucial for pericyte recruitment and BBB maintenance.

Our findings demonstrated that GPR126 is a canonical Wnt target, linking brain Wnt signaling to BBB maturation, including BEC structure, permeability regulation, basal membrane deposition, and pericyte coverage. Our study also highlights GPR126’s critical role in CNS vascular development. GPR126 depletion impairs BEC proliferation and migration, crucial for angiogenesis. In 3D BEC spheroid cultures from *Gpr126^iECKO^* mice, sprout formation and elongation were significantly reduced, emphasizing GPR126’s role in sprouting angiogenesis. Live-cell imaging showed decreased sprout length and vessel regression without GPR126. Similar defects in retinal angiogenesis, including reduced tip cells, vascular density, and delayed vascularization, underscore GPR126’s role in vessel sprouting and remodeling. Additionally, an aortic ring assay showed diminished endothelial sprouting and branching in *Gpr126^iECKO^* mice, reinforcing GPR126’s importance in angiogenesis across different vascular contexts.

Given that brain angiogenesis has been demonstrated to be closely linked to the formation and maturation of the BBB (60), our study reveals the involvement of endothelial GPR126 in both the development of a functionally impaired BBB and angiogenesis. Determining whether GPR126’s role in BBB formation is a result of the angiogenesis phenotype or represents an independent, concurrent process is not straightforward, and will require further investigation in future studies. Indeed, while unraveling the molecular mechanisms of GPR126 during BBB formation, our transcriptomic analysis revealed that GPR126 regulated genes related to angiogenesis, including *Lrp1*. GPR126 sustained LRP1 expression via the cAMP/CREB pathway upon collagen IV binding. Conversely, GPR126 expression is downregulated in brain ECs lacking LRP1, indicating a transcriptional loop between LRP1 and GPR126. Recent studies have linked LRP1 to EC growth, migration, and angiogenesis, although the molecular mechanisms remain largely unknown (63, 72). We observed a significant correlation between *Gpr126* and *Lrp1* mRNA levels from E10.0 in the embryonic stage to P18, coinciding with angiogenesis onset and prior to mature brain vasculature formation. Additionally, inactivating *Gpr126* in embryonic brain vessels at E14.5 reduced *Lrp1* transcript levels, highlighting their collaborative role during BBB development.

Considering that LRP1 facilitates the endocytosis of many ligand-receptor complexes (61), including β_1_ integrin (73), and several GPCRs work in concert with β_1_ integrin for directed cell migration (74, 75), it is not surprising that GPR126 regulates angiogenesis by exploiting a similar machinery. Indeed, we showed that GPR126 interacted with LRP1 and β_1_ integrin, regulating EC migration during angiogenesis by balancing the levels of β_1_ integrin activation and recycling through a dynamin-dependent mechanism. Notably, dynamin is involved in multiple endocytic mechanisms (e.g., clathrin mediated, caveolin mediated, non-clathrin endocytosis, fast endophilin-mediated endocytosis). Thus, the fact that GPR126-depleted cells showed an increase of dynamin-dependent endocytosis of some cargoes (i.e., cadaverine) and a decrease of others (i.e., β_1_ integrin) might indicate a differential impact on distinct endocytic mechanisms.

Our findings underscored GPR126 as a key regulator of BBB permeability, maintaining low transcytosis activity in brain endothelium. Future research should prioritize elucidating the regulatory mechanisms governing BBB permeability, focusing on paracellular and transcellular transport pathways. Beyond its homeostatic role, GPR126 governed EC proliferation, sprouting, and migration. Understanding the interactions between GPR126, LRP1, and β_1_ integrin is crucial for insights into angiogenesis and potential therapeutic interventions.

In summary, GPR126 has a multifaceted role in CNS vascular biology, encompassing BBB development and angiogenesis. Further exploration of GPR126’s molecular mechanisms is essential for understanding CNS vascular homeostasis and may offer therapeutic avenues for neurological disorders characterized by vascular dysfunction.

## Methods

Detailed information on materials and methods is provided in Supplemental Methods.

### Sex as a biological variable.

Our study examined male and female animals, and similar findings are reported for both sexes.

### Mice.

The following mouse strains were used: *Gpr126^fl/fl^* (model TF0269, Taconic Biosciences GmbH, Lexicon Pharmaceuticals), *Cdh5(PAC)*-CreER^T2^ (13073, Taconic Biosciences GmbH), conditional knockin of tdTomato-T2A-dnTCF4 (*dnTcf4^flox(stop)/flox(stop)^*) in the ROSA26 locus (generated by Taconic Biosciences GmbH), and C57BL/6J mice (Charles River). *Gpr126^fl/fl^* mice were generated by Lexicon Pharmaceuticals using the following strategy: exons 3 and 4 of the *Gpr126* gene were floxed with *loxP* sequences. A neomycin (NEO) cassette flanked by FRT sites was included in the floxed *Gpr126* sequence. Heterozygous animals were obtained on a mixed background (129/SvEv-C57BL/6). The marker-assisted accelerated backcrossing method (MAX-BAX, Charles River) was used to obtain *Gpr126^fl/fl^* strain on a pure C57BL/6J background.

The *Gpr126^fl/fl^* mice were crossed with *Cdh5(PAC)*-CreER^T2^ mice to generate a *Gpr126* conditional knockout (*Gpr126^iECKO^*).

The *dnTcf4^flox(stop)/flox(stop)^* conditional knockin mice were interbred with the *Cdh5(PAC)*-CreER^T2^ transgenic line. *DnTcf4^flox(stop)/wt^Cdh5(PAC)-*CreER^T2+^ mice were interbred with *dnTcf4^flox(stop)/flox(stop)^* mice to generate litters containing *dnTcf4^flox(stop)/flox(stop)^*/*Cdh5(PAC)*-CreER^T2+^ (*dnTCF4^iECKI^*) mice and control *dnTcf4^flox(stop)/flox(stop)^*/*Cdh5(PAC)*-CreER^T2^ mice (WT) (21).

### Statistics.

The mice were grouped and randomized during the experiments using the online randomization tool Research Randomizer (http://www.randomizer.org). Moreover, both males and females (in equal proportions) within each experiment originated from different litters.

The normality of the data sets was assessed using Shapiro-Wilk normality tests. For data sets with normal distributions, 2-tailed, 2-sided, unpaired Welch’s *t* tests (for pairwise comparison) or 1-way Brown-Forsythe ANOVA followed by Dunnett’s T3 tests (for post hoc multiple comparisons) or 2-way ANOVA were used. Non-parametric (Mann-Whitney) tests were applied to data sets that did not show normal distributions. Wherever applicable, details about the statistical test applied and the sample sizes (*n*) are provided in the figure legends. *P* values less than 0.05 were considered statistically significant. The standard software package GraphPad Prism (v9.4.0 and v10.2.3) was used.

### Study approval.

All animal procedures were conducted in accordance with the Institutional Animal Care and Use Committee regulations of both IFOM and the San Raffaele Scientific Institute and complied with the guidelines outlined in the Principles of Laboratory Animal Care of both IFOM and the San Raffaele Scientific Institute (directive 86/609/EEC). Additionally, they received approval from the Italian Ministry of Health (Authorization 675/2021-PR).

### Data availability.

RNA sequencing data are available through the European Molecular Biology Laboratory–European Bioinformatics Institute (EMBL-EBI) ArrayExpress BioStudies portal with accession number E-MTAB-13914. All supporting data are provided in the Supporting Data Values file.

## Author contributions

MG, NK, and DI conceived and designed the study. NK, MG, and AAS performed the in vitro experiments. CM, NK, AAS, and LC performed the in vivo experiments and analyzed the results with MG. AFB performed endocytosis/transcytosis assay and analyzed the results with SS and MG. CB and CF provided neonatal astrocytes. EM and SM performed imaging analysis. MGL, CC, NK, ED, GM, MI, KB, MB, GSG, AP, MD, and MDG contributed to scientific discussions. FI and FZ performed the bioinformatics analyses. NK, GVB, and AAM performed the electron microscopy experiments. MG and DI wrote the manuscript, which was revised by CC, ED, and MB.

## Supplementary Material

Supplemental data

Unedited blot and gel images

Supplemental video 1

Supporting data values

## Version 1 08/01/2024

Electronic publication
