# Supplementary material for: GPR126 is a specifier of blood-brain barrier formation in the mouse central nervous system
Source: J Clin Invest. 2024 Jun 6;134(15):e165368. doi: 10.1172/JCI165368 (PMC11290973; doi:10.1172/JCI165368)
Supplement: Supplemental data [file jci-134-165368-s057.pdf]

Supplemental Material

Gpr126 regulates blood–brain barrier formation in the mouse central nervous system.

Nikolaos Kakogiannos[1*], Anna Agata Scalise[1*], Emanuele Martini[1,2], Claudio Maderna[1], Andrea Francesco Benvenuto[3], Michele D'Antonio[4], Laura Carmignani[1], Serena Magni[1], Giorgia Serena Gullotta[5], Maria Grazia Lampugnani[1], Fabio Iannelli[1], Galina V. Beznoussenko[1], Alexander A. Mironov[1], Camilla Cerutti[3], Katie Bentley[6,7], Andrew Philippides[8], Federica Zanardi[1], Marco Bacigaluppi[5,9], Sara Sigismund[2,3], Claudia Bassani[10], Cinthia Farina[10], Gianvito Martino[5,9], Marco De Giovanni[4], Elisabetta Dejana[1], Matteo Iannacone[4,9,#] Donato Inverso[4,9,#,§] and Monica Giannotta[1,4,#,§]

Material and Methods

Mice

Tamoxifen (T5648; Sigma-Aldrich) was dissolved in 10% ethanol-sunflower oil (019187_017; Farmalabor, 10 mg/mL) and administrated to the mice to induce Cre activity and genetic modifications. For inducible endothelial-specific deletion of *Gpr126*, *Cdh5(PAC)*-CreER^{T2}- *Gpr126*$^{flox/flox}$ pups were injected subcutaneously with tamoxifen at the age of post-natal day (P)1 (20 µg), P2 (20 µg), P3 (30 µg), P4 (30 µg). Pregnant females were identified following overnight mating, and the following morning, they were examined for the presence of a vaginal plug; if present, this was recorded as day 0.5 post-coitus (pc). Pregnant females were intraperitoneally treated with three injections of 1 mg tamoxifen on days 9, 10 and 11 pc. In all genetic experiments, the control mice (WT) refer to tamoxifen-injected mice lacking the Cre transgene. All mice underwent genotyping to confirm the mutations, as previously described [for *Gpr126* (1); for *Cre* (2)].

Cells and treatments

Freshly isolated brain endothelial cells (fBECs) and primary cultured (c)BECs

WT and *Gpr126*^{iECKO} ECs were derived from brains of embryos and pups at different post-natal stages upon tamoxifen treatment, as previously reported. Briefly, the brains were digested enzymatically in combination with gentle dissociation (MACS; Neural tissue dissociation kit [130-092-628] for embryos and pups <P7; adult brain dissociation kit [130-107-677] for pups P8 and older; Miltenyi Biotech). After dissociation, the myelin cell debris and erythrocytes were removed according to the manufacturer protocol. ECs were enriched by depletion of CD45-positive cells with CD45 microbeads (30-052-301;

Miltenyi Biotech). After AN2 re-expression according to the manufacturer protocol, pericytes and astrocytes were depleted using AN2 microbeads (130-097-171; Miltenyi Biotech) and ACSA-2 microbeads (130-097-678; Miltenyi Biotech), respectively, followed by positive selection using CD31 microbeads (30-097-418; Miltenyi Biotech).

Alternatively, EC isolation and cultured brain microvascular fragments (referred to as primary cBECs in the main text) were processed as previously described (3, 4). Capillary fragments were seeded into rat collagen I (354236; BD Biosciences)-coated wells (0.5 brains/4 cm^2) and cultured in Dulbecco's modified Eagle's medium (DMEM) plus GlutaMAX (10564011; ThermoFisher Scientific), 20% fetal bovine serum (SH30070.03; Hyclone), 100 mg/mL heparin (H3149; Sigma-Aldrich), and 5 mg/mL Endothelial Cell Growth Supplement (E2759; Sigma-Aldrich). After 3 days of puromycin selection (4 mg/mL; AG-CN2-0078; Adipogen), cBECs were treated with purified Wnt3a (100 ng/mL; 1324-WN; R&D) or purified Wnt5a (250 ng/mL; 645-WN; R&D), or Wnt7a (100 ng/mL; CSB-MP026141MO, Cusabio), or Wnt7b (100 ng/mL; CSB-MP026142MO, Cusabio) in complete medium, every other day for a duration of 5 days. Alternatively, cBECs cells were treated with L-supernatant or Wnt3a-conditioned medium diluted 1:2 in complete medium, every day for a duration of 5 days (5). For the in vitro inhibition of Wnt/β-catenin signalling, cBECs were treated with MSAB (30 μM; HY-120697; MedChemExpress LLC), or IWR-1 (10 μM; HY-12238; MedChemExpress LLC) either alone or in combination of purified Wnt3a (100 ng/mL; 1324-WN; R&D) in complete medium every other day for a duration of 5 days.

For GPR126 receptor activation, WT and *Gpr126*^{iECKO} cBECs were treated with 5 μg/mL collagen IV (3410-010-01; Cultrex) or phosphate-buffered saline (PBS) for 2 days in DMEM plus GlutaMAX and 1% fetal bovine serum (Hyclone, starving medium). cBECs were cultured in starving medium for 16 h prior to treatment for 45 min with collagen IV. The final cell pellets were washed with PBS and processed for protein or RNA extraction.

Lung endothelial cells in culture (cLEC)

ECs were derived from lungs of WT adult mice (8 weeks old). Briefly, the lungs were digested enzymatically in combination with gentle dissociation (MACS; lung dissociation kits; 130-095-927; Miltenyi Biotech). After dissociation, the ECs were enriched by depletion of CD45-positive cells with CD45 microbeads (30-052-301; Miltenyi Biotech). After AN2 and CD326 re-expression according to the manufacturer protocol, immune cells, lymphatic ECs, fibroblasts, epithelial cells, smooth muscle

cells, and pericytes were removed using the following microbeads: anti-CD45 (130-052-301), anti-CD90.2 (130-121-278), anti-CD326 (130-105-958), anti-CD138 (130-098-257) and anti-AN2 (130-097-170). Finally, the ECs were positively selected using CD31 microbeads (30-097-418; Miltenyi Biotech). LECs were seeded into 0.1% gelatin (214340; Difco)-coated wells (1 lung/ 2 cm^2) and cultured in DMEM plus GlutaMAX (10564011; ThermoFisher Scientific), 20% fetal bovine serum (Hyclone), 100 mg/mL heparin (H3149; Sigma-Aldrich), and 5 mg/mL Endothelial Cell Growth Supplement (E2759; Sigma-Aldrich). After 5 days, cLECs were treated with purified Wnt3a (100 ng/mL; 1324-WN; R&D), in complete medium described above, every other day for a duration of 5 days. The final cell pellets were washed with PBS and processed for protein or RNA extraction.

Immortalized brain endothelial cells (iBECs)

Brain ECs were isolated from *Gpr126$^{flox/flox}$* pups (P9) (as described above for cBECs), and then seeded into collagen I (354236; BD Biosciences)-coated wells (1 brain/ 2 cm^2) and cultured in MCDB-131 (10372-019; ThermoFisher Scientific), 20% fetal bovine serum (Hyclone), 100 mg/mL heparin (H3149; Sigma-Aldrich), and 5 mg/mL Endothelial Cell Growth Supplement (E2759; Sigma-Aldrich) (complete medium). After a day, the cells were immortalized in culture through retroviral expression of polyoma middle T antigen, as described previously (6), and then left to grow for 4-5 weeks, changing the medium every 3-4 days. Gpr126 was inactivated by treating the cultured iBECs with TAT-Cre recombinase (100 µg/mL) and chloroquine (50 µM) (C6628; Sigma-Aldrich) for 1 h in Hyclone medium. Finally, iBECs were cultured on plates coated with 0.1% gelatin in complete medium. For the in vitro activation/inhibition of Wnt/β-catenin signalling, iBECs were cultured on 12-well plates coated with 0.5% gelatin embedded with purified Wnt3a (100 ng/mL; 1324-WN; R&D) or Wnt7a (100 ng/mL; CSB-MP026141MO, Cusabio), or Wnt7b (100 ng/mL; CSB-MP026142MO, Cusabio) either alone or in combination with IWR-1 (10 µM; HY-12238; MedChemExpress LLC) for 24 h.

Primary mouse astrocytes

Brains were extracted from new-born C57BL/6J wild type mice, mechanically dissociated, and digested with Neural Tissue Dissociation Kit ([130-092-628], Miltenyi Biotec). Mixed cultures were maintained in DMEM-high glucose medium containing 1% Pen/Strep, 200 mM l-Glutamine, 100 mM Sodium Pyruvate and 10% FBS (Euroclone, Italy)). Ten-day-old primary cultures were vigorously shaken to discard microglia and oligodendrocytes. The remaining adherent cells were detached and, following an

adhesion step, non-adherent cells were reseeded on poly-d-lysine (P4707, Sigma-Aldrich) coated flasks for 4 days. Cells were then expanded without any coating and used for experiments by passage 10. Purity of astrocyte cultures was >95% according to GFAP immunofluorescence. For co-culture experiments, primary mouse astrocytes were seeded at 130,000 cells per well in 6-well plates and allowed to achieve complete adherence. After 24 h, iBECs (250,000 cells per well) were cultured alone or added to neonatal astrocytes to allow direct contact between the two cell types in complete astrocyte medium. After 48 h, the cells were trypsinized, and astrocyte depletion was performed using ACSA-2 microbeads (130-097-678; Miltenyi Biotech), followed by a positive selection of ECs using CD31 microbeads (30-097-418; Miltenyi Biotech). To inhibit Wnt secretion, primary mouse astrocytes were pre-treated with Wnt-59 (2 μM; HY-15659; MedChemExpress LLC) for 2 h before adding iBECs in complete medium.

In vivo cadaverine permeability assay

Lysine-fixable, Alexa Fluor-555 conjugated cadaverine (3.125 mg/mL in saline; 950 Da; A30677; ThermoFisher Scientific) was injected intraperitoneally in mice at P18 and adults (25 mg/kg). The circulation time was 2 h. The animals were anesthesised by intraperitoneal injection of avertin (20 mg/kg; T48402; Sigma-Aldrich) 20 min before sacrifice. Then, the mice were perfused for 1 min to 2 min with Hank's balanced salt solution (14025-050; ThermoFisher Scientific), followed by 5 min perfusion with 4% paraformaldehyde (PFA) in PBS, pH 7.2. The brains from 3 animals per group were dissected out and fixed for up to 8 h by immersion in 4% PFA at 4 °C, then washed in PBS, and prepared for vibratome sectioning. Brains were fully sectioned. Sagittal vibratome sections were incubated with anti CD93 (AF1696, R&D Systems) in blocking/ permeabilisation solution (5% donkey serum [017-000-1210; Jackson Immuno Research], 0.3% Triton X-100, in PBS) overnight at 4 °C. This was followed by incubation with anti-sheep Alexa Fluor 488 (713-545-147; Jackson Immuno Research). For more details see Tissue processing and immunohistochemistry.

Endocytosis assay

cBECs and iBECs were cultured on μ-Slide 8 Well high (80806, Ibidi). For the uptake assays with AlexaFluor-555-conjugated cadaverine and AlexaFluor-647-conjugated transferrin (T23366, Thermofisher), cells were either pretreated with Dynasore 20 μM (dynamin inhibitor, D7693, Sigma Aldrich) for 30 min or vehicle. Cells were then incubated with 0.1 μM cadaverine or 50 μg/mL transferrin at 37°C for 15 minutes (for cadaverine) and 30 minutes (for transferrin), to allow internalization.

Following this step, cells were put on ice and any remaining fluorescently labeled molecules at the cell surface were removed with acid wash treatments (0.1 M glycine, HCl pH= 2.2, three washes of 45 sec/each). Cells were then washed with PBS and fixed in 4% PFA for 10 min. Subsequently, cells were permeabilized with 0.1% Triton for 8 min, blocked with 1% BSA for 1 h and nuclei were stained with DAPI (4′,6-diamidino-2-phenylindole). Endocytosis was visualized using confocal microscopy (SP8 CSU, Leica). Quantification of endocytosis was performed by analyzing the integrated density of fluorescence and normalizing it to the number of cells (Fiji, ImageJ).

For integrin endocytosis assays, cells were either treated with 20 μM Dynasore or with vehicle. Plasma membrane integrins were then labelled with an antibody recognizing the active conformation of β1-integrins (active CD29 Clone 9EG7, 553715; BD Biosciences) at 4 °C for 1 h. Cells were immediately fixed for plasma membrane staining or switched to 37 °C for 15 min to induce endocytosis of the protein-antibody complex. Surface-bound antibody was removed by acid wash treatment (0.1 M glycine, HCl pH= 2.2, three washes of 45 sec/each). Cells were then washed with PBS and fixed in 4% PFA for 10 min. Subsequently, cells were permeabilized with 0.1% Triton for 8 min, blocked with 1% BSA for 1 h, and incubated with a fluorescent secondary antibody and nuclei were stained with DAPI (4′,6-diamidino-2-phenylindole). Endocytosis was visualized and quantified as described above.

Transcytosis assay

iBECs were cultured to confluence on 0.5% gelatin-coated 6.5 mm Transwell® inserts with a 5.0 μm pore polycarbonate membrane (3413; Corning). The integrity of the cell monolayer was verified by measuring trans-endothelial electrical resistance (TEER) (Millicell-ERS, Millipore). Subsequently, cells in the upper chamber were incubated with AlexaFluor-555-conjugated cadaverine or AlexaFluor-488-conjugated transferrin, either in the presence or absence of 20 μM Dynasore, for a duration of 120 min. After incubation, the medium from the lower chamber was collected, and its fluorescence was measured using the GloMax® Discover Microplate Reader (Promega) to assess the transcytosis of the fluorescent molecules. At the end of the assay, the endothelial monolayer on the permeable supports were fixed with 4% paraformaldehyde for 10 min at room temperature. Once fixed, the cells were stained with Gram's crystal violet solution (1092180500; Merk) for 5 min, and then washed three times with PBS with Ca^{2+} and Mg^{2+}. The monolayers were visualized using a widefield microscope (EVOS-Fl).

Lentiviral preparation

To stably express GPR126, a custom-made lentiviral vector was purchased from Vector Builder. This is a bicistronic expression vector that uses a self-cleaving 2A peptide to co-express murine GPR126 and EGFP under the control of a single CMV promoter. *Lrp1* Mouse shRNA Plasmid (Locus ID 16971, #TL512679) and a non-targeting shRNA (#TR30023) as a control were purchased from Origene. The lentiviruses were generated in HEK 293T cells, as described previously (7). The lentiviral vectors were produced (8) with the packaging plasmids donated by L. Naldini (HSR-TIGET, San Raffaele Telethon Institute for Gene Therapy, Milan, Italy). Infectious viruses were purified and titred using standard techniques. Two consecutive cycles of infections were performed in complete medium.

***In-situ* hybridization using the RNAscope technique**

In-situ hybridization using the RNAscope technique was performed according to the manufacturer instructions (Advanced Cell Diagnostics), using multiplex fluorescent Reagent Kit v2. Briefly, brains derived from WT mice were dissected out and post-fixed in 4% formaldehyde overnight at 4 °C, and then processed using the standard paraffin-embedding procedure.

For paraffin-embedding, the brains were processed using a Diapath automatic processor, as follows. Tissues were dehydrated through 70% (60 min), 2 changes of 95% (90 min each), and 3 changes of 99% (60 min each) ethanol, cleared through 3 changes of xylene (90 min each), and finally immersed in 3 changes of paraffin (1 h each). Samples were embedded in a paraffin block and prepared for sectioning. Four-µm-thick sagittal sections were deparaffinized and pretreated with H_2O_2, followed by target retrieval and a 15 min incubation with protease IV. The probes *Cldn5* (#491611-C2; Advanced Cell Diagnostics) and *Gpr126* (#1038161-C1; Advanced Cell Diagnostics) were hybridized on the tissue sections for 2 h at 4 °C (HybEZ oven; Advanced Cell Diagnostics), followed by signal amplification with the reagents included in Reagent Kit v2. The signal was detected for *Cldn5* with OPAL520 (SKU FP1487001KT; Akoya Bioscience) and for *Gpr126* with OPAL650 (SKU FP1496001KT; Akoya Bioscience), and tissue sections were counterstained with DAPI (Advanced Cell Diagnostics). The probe was designed to recognize exon 3 of the *Gpr126* gene, which is deleted upon gene inactivation. iBECs without and with a stably expressed murine GPR126-Myc tagged protein and after *Gpr126* gene inactivation were used to validate the probe. Indeed, iBECs (45000 cells/ 2 cm^2) or cBECs (1 brain/ 2 cm^2) were seeded on chambered cell culture slides (#354118, BD Falcon) that were previously coated with glutaraldehyde-crosslinked gelatin as follows: the culture supports were incubated for 1 h at RT

with 0.5% gelatin, followed by crosslinking with 2% glutaraldehyde solution (G7776 Sigma-Aldrich) for 15 min at RT. The glutaraldehyde was then replaced by 70% ethanol. After 1 h, 5 washes with sterile PBS were followed by an overnight incubation with PBS containing 2 mM glycine. Before cell seeding, the slides were washed 5 times with sterile PBS.

In-situ hybridization was performed using RNAscope® protocol for adherent cells cultured on coverslips according to the manufacturer instructions.

Cells were washed once with PBS, before fixation with 4% PFA for 30 min at RT and then two further washes with PBS. Coverslips were immersed in 50% ethanol, then 75% and finally 100%. Coverslips were rehydrated using the same sequence of immersions in reverse and then washed with PBS for 10 min. This was removed and a 15 min incubation was performed with Protease III (1:15 dilution) at RT before two washes with PBS. The probes *Cldn5* (#491611-C2; Advanced Cell Diagnostics) and *Lrp1* (#465231-C1; Advanced Cell Diagnostics) were hybridized on the tissue sections for 2 h at 4 °C (HybEZ oven; Advanced Cell Diagnostics), followed by signal amplification with the reagents included in Reagent Kit v2. The signal was detected for *Cldn5* with OPAL520 (SKU FP1487001KT; Akoya Bioscience) and for *Lrp1* with OPAL650 (SKU FP1496001KT; Akoya Bioscience), and tissue sections were counterstained with DAPI (Advanced Cell Diagnostics)

Fluorescent tilescan images that visualized the signals for mRNA expression were acquired using a fluorescent microscope (DMi8; Leica), at 40× magnification. Representative close-up images were then acquired with the confocal microscope at 63× magnification. For comparison purposes, different sample images of the same probe combinations were acquired under constant acquisition settings.

Gene expression analysis

Affymetrix

Total mRNA was extracted from cBECs after treatment with Wnt3a-conditioned medium *versus* control medium for 5 days. Gene expression was analyzed using the GeneChip ST 1.0 array (Affymetrix) covering 29,000 murine genes. For each condition, mRNA obtained from three different experiments was assayed to account for biological variability. Genes differentially expressed between the two conditions were identified as those having at least a two-fold change and $P<0.05$ using Fisher's least significant difference tests.

RNA sequencing

Total RNA was extracted from fBECs of WT and *Gpr126^{iECKO}* mice at P18 (two biological replicates under each condition (n=4 WT and 4 *Gpr126^{iECKO}* mice for each replicate) using Maxwell RSC simplyRNA tissue kits with the Maxwell RSC Instruments (Promega Corporation). The amount of RNA was measured using the Nanodrop technique and its integrity assessed using Agilent Bioanalyzer 2100 with Nano RNA kits (RIN > 8). Libraries for RNA sequencing were prepared following the manufacturer protocols for transcriptome sequencing with the Illumina NextSeq 550DX sequencer (Illumina). mRNA-seq indexed library preparation was performed starting from 500 ng of total RNA with the Illumina ligation stranded mRNA (Illumina) according to the manufacturer instructions. Indexed libraries were quality controlled on an Agilent Bioanalyzer 2100 with High Sensitivity DNA kits, quantified with Qubit HS DNA, normalized and pooled to perform multiplexed sequencing runs. A PhiX (1%) control was added to the sequencing pool, to serve as a positive run control. Sequencing was performed in PE mode (2×75nt) on an Illumina NextSeq550Dx platform, to generate on average 50 million PE reads per sample, on average.

RNA-sequencing data analysis

Reads were aligned to the mm10 assembly mouse reference genome using the STAR aligner [v 2.6.1d (9)] and were quantified using Salmon [v1.4.0 (10)]. Differential gene expression analysis was performed using the Bioconductor package DESeq2 [v1.30.0 (11)] that estimates variance-mean dependence in count data from high-throughput sequencing data and tests for differential expression exploiting a negative binomial distribution-based model.

Preranked gene set enrichment analysis (GSEA) for evaluating pathway enrichment in transcriptional data was carried out using the Bioconductor package fgsea (12), taking advantage of the GO Biological process gene sets available from the GSEA Molecular Signatures Database (https://www.gsea-msigdb.org/gsea/msigdb/genesets.jsp?collections).

RNA-seq data is being uploaded to the EMBL-EBI functional genomics data collection (ArrayExpress) and can be accessed with the accession number E-MTAB-13914.

Quantitative RT-PCR analysis

Total RNA was extracted from fBECs from WT and *Gpr126^{iECKO}* mice at P18 using Maxwell RSC simplyRNA tissue kits with a Maxwell RSC instrument (Promega Corporation). The amount of RNA was measured using the Nanodrop technique and reverse transcribed with random hexamers (High-Capacity cDNA Archive kits; Applied Biosystems), following the manufacturer instructions. For gene

expression analysis, 5 ng cDNA was amplified (in triplicate) in a reaction volume of 10 μL that contained the following reagents: 5 μL "TaqMan Fast Advanced Master Mix (4444557; Applied Biosystems), 0.5 μL TaqMan Gene expression assay 20× (ThermoFisher Scientific). For the time course of Gpr126 expression, the samples were preamplified using TaqMan PreAmp Master Mix (ThermoFisher Scientific), then diluited 1:4 with TE 1× buffer, and 2 μL/well was used for RT-PCR analysis. Real-time PCR was carried out on a real-time PCR system (7500; ThermoFisher Scientific) using a pre-PCR step of 20 s at 95 °C, followed by 4 cycles of 1 s at 95 °C and 20 s at 60 °C. A specific TaqMan assay (ThermoFisher Scientific) was used for each gene. Raw data (Ct) were analyzed using the Biogazelle qbase plus software, and the fold-changes are expressed as calibrated normalized relative quantities (CNRQs) with standard error (SE). The GeNorm Software chose GAPDH and 18s as the best housekeeping genes, and the geometric mean of GAPDH and 18S was used to normalise the data.

Electron microscopy (EM)

WT and *Gpr126^{iECKO}* mice were anesthetized by intraperitoneal injection of avertin 20 mg/kg (T48402, Sigma) and perfused with 2.5% PFA and 2.5% glutaraldehyde (15949; EMS) mixture in 0.2 M sodium cacodylate pH 7.2 at the physiological pressure for 5 min (1.8 ml/min for adult mice and 0.6 ml/min for pups at P18). Brain cortex was excised and processed for EM examination and EM tomography as previously described ((13)). For immuno-labelling, the grids were prepared as previously described (14). Briefly, the grids were incubated for 10 min in 0.5% BSA-c in PBS (bovine serum albumin-c™, acetylated, 10% in water; Aurion) to prevent nonspecific labelling, and then incubated on 10 μL droplets of primary anti-GPR126 (ab218046; Abcam) diluted 1:10 for 2 h, and then with protein-A gold 10 nm (PAG10, CMC, Utrecht, The Netherlands) 1:50 diluted in blocking solution for 20 min at room temperature. The specificity of the anti-GPR126 was confirmed in GPR126-KO ECs. The grids were rinsed six times with 0.1% BSA-c in PBS and post-fixed with 1% glutaraldehyde in 0.15 M HEPES (pH 7.3) for 5 min. The primary antibody was monoclonal anti-β1 integrin (AF 2325; R&D Systems) at the dilution of 1:10. The secondary antibody was the rabbit polyclonal anti-goat immunoglobulin antibody (bridge antibody; Jackson Immuno Research) diluted 1:250, and then with protein-A gold 5 nm (PAG5, CMC, Utrecht, The Netherlands) diluted in blocking solution 1:50 for 20 min at room temperature. Finally, the grids were stained for 10 min in 1.8% methyl cellulose plus 0.4% uranyl acetate, on ice. The grids were retrieved and after air-drying, they were examined under an electron microscope (FEI, Tecnai20; ThermoFisher Scientific, The Netherlands).

Tissue processing and immunohistochemistry

All immunostainings of both WT and *Gpr126*^{iECKO} mice were carried out simultaneously and under the same conditions. Tissues were prepared and processed for immunohistochemical analysis as described previously (15, 16). Briefly, mice were anesthetized by intraperitoneal injection of Avertin 20 mg/kg (T48402, Sigma), and perfused with 1% PFA in PBS. The mouse brains were carefully dissected and post-fixed overnight by immersion in 4% PFA at 4 °C. The next day, they were washed in PBS and processed for sectioning as follows.

For vibratome sections, brains were embedded in 4% low-melting-point agarose, sectioned with a vibratome (100 mm thickness; VT1200s; Leica) and immune stained. Sagittal vibratome sections were incubated in PBST solution (PBS with 0.3% Triton X-100) supplemented with 5% donkey serum and containing the primary antibodies, overnight at 4 °C. This was followed by washes with PBST, followed by the secondary antibody solutions (overnight at 4 °C). Sections were then washed, post-fixed for 2 min in 1% PFA, and mounted in Vectashield that contained DAPI (H-1200; Vector). Detection of claudin-5 (ab15106; Abcam) and PLVAP (550563 clone MECA-32; BD Biosciences) was carried out as above, with the only exception that brains were post-fixed overnight in 100% methanol at 4 °C (instead of 4% PFA) and rehydrated for 1 h in PBS before sectioning.

For cryosectioning, fixed brains were cryo-protected in 30% sucrose overnight at 4 °C, embedded in Killik embedding medium (059801; BioOptica) and frozen on the surface of the cold isopentane/2-methylbutane next to the liquid nitrogen. Sections (10 µm) were cut using a cryostat (Leica), then mounted on positively charged glass slides and stained as described above.

To analyze the mouse retina vascular phenotype, the eyes were collected and fixed in 4% PFA in PBS for 2 h at 4 °C. Retinas were dissected as described previously (16) and incubated at 4 °C overnight in primary antibodies diluted in PBSTC buffer (PBS with 0.5% Triton X-100, 0.1 mM $CaCl_2$) supplemented with 5% donkey serum, followed by the suitable species-specific Alexa Fluor-conjugated secondary antibody staining, overnight at 4 °C. These samples were then flat mounted on glass microscope slides with Prolong Gold antifade reagent (P36930; ThermoFisher Scientific). For immunofluorescence analysis, negative controls using isotype matched normal IgG were included to check for antibody specificity.

RNA interference

cBECs derived from WT pups at p18 were seeded into collagen I-coated wells (1.5 brain/ 10 cm^2) and cultured in DMEM complete medium. After 3 days of puromycin selection, they were transfected with either non-targeting siRNAs (StealthTM RNAi Negative Control Duplexes; Invitrogen) or siRNAs against mouse *Gpr126* (MSS278013: #13 CGACUGCCAAGGGCCUGUCAUUUAA MSS210995: #95 GCCUCCAAAUUUGCUUGAGAAUUUA; MSS210997: #97 CCGUGUUACCCUAAUGACUACCCUA, ThermoFisher Scientific). Transfection was performed using Lipofectamine 2000 (LS11668019; ThermoFischer Scientific) or Lipofectamine RNAiMAX (13778150; ThermoFischer Scientific), according to the manufacturer instructions.

Western blotting

Total proteins were extracted by solubilizing the cells in boiling sample buffer (2×; 2.5% sodium dodecyl sulfate, 20% glycerol, 0.125 M Tris-HCl, pH 6.8) enriched in protease inhibitor R cocktail set III (EDTA free; 539134; Calbiochem) and phosphatase inhibitors (cocktail 2; P5726; and cocktail 3; P0044; Sigma-Aldrich). Lysates were incubated for 5 min at 100 °C, to promote protein denaturation, and then centrifuged at 16,000× *g* for 5 min, to pellet the cell debris. Protein concentrations were determined using BCA Protein Assay kits (23225; Pierce), according to the manufacturer instructions. Equal amounts of protein were loaded onto custom gels (Bio-Rad or Invitrogen), separated by SDS-PAGE, transferred to membranes (Protran nitrocellulose hybridisation transfer membrane; pore size, 0.2 µm; 10600001; GE Healthcare), and blocked for 1 h at room temperature in 5% milk or 5% bovine serum albumin, in 0.05% Tween-20 in Tris-buffered saline (TBS). The membranes were incubated overnight at 4 °C or for 1 h at room temperature, with the primary antibodies diluted in blocking solution. The membranes were rinsed three times with washing solution (0.05% Tween-20 in TBS) and incubated for 1 h at room temperature with horseradish-peroxidase-linked secondary antibodies (diluted in blocking solution). The specific binding was detected by Super Signal West Femto Maximum Sensitivity Substrate (34096; ThermoFisher Scientific) or Super Signal Dura Extended Duration Substrate (34076; ThermoFisher Scientific), using a ChemiDoc MP system (BioRad). The molecular masses of the proteins were estimated relative to the electrophoretic mobility of a co-transferred prestained protein marker (Precision Plus Protein Standards Dual Colour; 161-0374; BioRad). Densitometry analysis of the bands was carried out used the Fiji software (open source; htpp://fiji.sc/) and with the band analysis tools of the ImageLab

software, version 4.1 (BioRad). Gapdh, vinculin, tubulin, or VE-cadherin (loading control) were used to normalize the quantified bands, as specified in the Figures.

Flow cytometry analysis

To obtain enriched fBECs by depletion of CD45-positive cells, the brains from 4 animals per group of WT and *Gpr126*^{iECKO} mice (P18) were processed as described above. Flow cytometry was performed using a Dako FACS instrument, Attune NxT flow cytometer (ThermoFisher Scientific). Phenotypic analysis was performed with the following antibodies: Rat anti-mouse active β1 integrin (active CD29 Clone 9EG7, 553715; BD Biosciences); Alexa647-Armenian hamster anti-mouse β1-integrin (total β1 integrin 102214; Biolegend); Alexa488-goat anti-mouse PECAM-1 (FAB3628G; R&D Systems). Fc receptor blocking reagent (130-092-575; Miltenyi) was used to block unwanted binding of antibodies to mouse cells expressing Fc receptors. Cy3 anti-rat secondary antibody (712-165-153; Jackson Immuno Research) was used to detect active β1 integrin. Cells were first gated to remove doublets, for light scatter and then for specific markers. Quantitative analysis was performed using Summit 4.2 from Dako, Kaluza software from Beckman Coulter. Alexa488 conjugated anti-PECAM-1 antibody was used to identify the EC population, where the proportions (%) of positive cells expressing active and total β1 integrin were analyzed.

Sprouting angiogenesis assay

For the angiogenic sprouting assay (17), WT and *Gpr126*^{iECKO} ECs were isolated from brains of pups at P18, as previously described. Briefly, after dissociation, myelin cell debris was removed according to the manufacturer protocol. ECs were enriched by depletion of CD45-positive cells with CD45 microbeads (30-052-301; Miltenyi Biotech), followed by positive selection using CD31 microbeads (30-097-418; Miltenyi Biotech). Then, the enriched ECs were resuspended in MCDB-131 complete medium containing 0.1% methylcellulose (4000 cP, #M0512; Sigma). For spheroid formation, 10000 cells per 25 μL methylcellulose medium were seeded in wells of U-bottomed 96-wells suspension plates and incubated for 5 days. Then, spheroids (25 μL) were collected and resuspended in 600 μL/well 3 mg/mL Type I rat tail collagen (High Conc collagen I, 354249; Corning) in 199 medium (21180021; ThermoFisher Scientific) and placed at 37 °C for 30 min. After polymerization of the collagen gel, the spheroids were stimulated with 80 ng/mL mVEGF-A (450-32; Peprotech) and 50 ng/mL FGFβ (450-33; Peprotech), to induce sprouting, for 48 h in complete medium. Pictures were acquired using the Thunder

imaging system and the 20× objective. Sprouting numbers and lengths were analyzed using the line tool of Fiji.

Aortic ring assay

Thoracic aortas were taken from WT and *Gpr126*^{iECKO} mice (P18) and cut into 1 mm rings after removal of the connective tissues. Rings were embedded between two layers of rat tail collagen, type I (A1048301, Thermo Fisher Scientific), and cultivated in MCDB-131 (Gibco) supplemented with 10% FBS and 30 ng/mL mVEGF-A (450-32; Peprotech). After 4 days of initial cultivation, samples were fixed in 4% PFA (Sigma) and analyzed by microscopy after immunostaining. To quantify the endothelial sprouting area and the number of branches, a custom Fiji plugin was developed. After manual drawing of the area to analyse, the sprouting area on the Maximum Projection of the IB4-647 channel is identified using the Triangle Threshold method. The automatic threshold is set after pre-processing the image with contrast enhancement, median and Gaussian filters, and rolling ball background subtraction. The sprouting was then measured as the number of branches in the skeletonized sprouted area.

Scratch wound migration assay

cBECs (2,5 brains /well) were cultured on 6-well plates coated with rat collagen I. After reaching confluency, the monolayers were scratched cross-wise with a p200 pipette tip, washed with MCDB-131 medium and 2% fetal bovine serum, and imaged at time 0 using an Optika Microscopy C-P6 FL imaging system and the 20× objective. Then the 6-well plates were incubated for 36 h and were imaged at the end point. The wound width at T0 (scratch time) and after 36 h was estimated as the average distance of three vertical lines drawn by hand between the two cell layers and always at the same positions along the wound, using the line tool of Fiji. The wound closure was then calculated, as normalizing using the T0 measurements.

Alternatively, cBECs were seeded into Incucyte® ImageLock 96-well plates (Sartorius) (1 brain / 4 wells), previously coated with rat tail collagen I. cBECs were labeled with 0.7 µM CellTracker™ Green CMFDA (C2925, Molecular Probes, Invitrogen) for 20 min at 37°C and pre-incubated with vehicle or 20 µM Dynasore in MCDB-131 medium and 2% fetal bovine serum (starving medium).

For the wound healing assays, confluent cells were scratched using the Incucyte 96-well Wound Maker tool (Sartorius), washed once to remove floating cells, and prevent reattachment in the wound area. The wells were replenished with starving medium containing vehicle or 20 µM Dynasore in the presence of

30 ng/mL mVEGF-A. The cells were imaged in the green channel using the 10× objective in the Incucyte S3 Live Cell Analysis System with Incucyte S3 software (Sartorius). Images were taken every 2 h for 30 h.

The Incucyte® S3 Live-Cell Analysis System's module Scratch Wound Cell Migration & Invasion Assay software was used to extract the area covered by the cells. The speed of closure was then measured as the slope of a linear fitting (using the SLOPE function in Microsoft Excel) of the area covered, considering all the frames acquired during the first 20 h.

Co-immunoprecipitation assay

Confluent monolayers of iBECs transfected with GFP or GPR126-Myc (2 ×10^7 cells) were solubilised in cold lysis CHAPS buffer (50 mM Tris-HCl, pH 7.4, 50 mM NaCl, 2 mM $MgCl_2$, 1 mM EDTA, 15 mM CHAPS (C5070; Sigma), protease inhibitors (4693116001; Roche) and phosphatase inhibitors (P2850, P5726; Sigma-Aldrich), and incubated on ice for 1 h. For GPR126-Myc immunoprecipitation, after preclearing with protein A-Sepharose, 4.5 mg of the protein extracts were incubated with anti-c-Myc agarose affinity gels, antibody (rabbit, A7470, Sigma) overnight at 4 °C. For β1 integrin immunoprecipitation, after preclearing with protein G-Sepharose 4B plus (101241, Thermo Fisher scientific) 9 mg protein extracts were incubated with goat anti-β1 integrin (AF2405; R&D Systems) or goat IgG (negative control, 705-005-003, Jackson immunoresearch) overnight at 4 °C, followed by incubation with protein G-Sepharose 4B plus for 1 h. After ten washing steps, the immune complexes were eluted in 100 µl elution buffer (8 M urea in 100 mM Tris-HCl, pH 7) and Western blotting samples were prepared by adding sample buffer (3× Laemmli Sample Buffer 1610747; BioRad) plus 3.57 M β-mercaptoethanol (2-mercaptoethanol, 63689; Sigma-Aldrich). The following antibodies were used for blotting: anti-Myc mouse (2276; Cell Signaling), anti-LRP1 α-chain mouse B411E2 (1:10000; WB), anti-integrin β1 goat (1:200; AF2405; R&D) anti-α1 integrin goat (ab243032; Abcam), and anti-α3 integrin goat (AF2787; R&D). Anti-β1 integrin rabbit (4706; Cell Signaling) was used to detect immunoprecipitated β1 integrin. Alternatively, confluent monolayers of iBECs were incubated with 80 µg/mL dithiobis-(succinimidyl) propionate (Pierce, Rockford, IL, USA) for 30 min at 37°C. The cells were lysed in RIPA buffer (100 mM Tris–HCl, pH 7.4, 150 mM NaCl, 1% Triton, 1% deoxycholic acid, 0.1% SDS, 2 mM $CaCl_2$, protease inhibitors and phosphatase inhibitors) and after preclearing with protein G-Sepharose 4B plus, 4.5 mg of the protein extracts were incubated with anti-c-myc agarose affinity gel antibody (mouse, 20168; Thermo Fisher Scientific) overnight at 4 °C. The immune

complexes were processed as previously described. The following antibodies were used for blotting: anti-Myc rabbit (2276; Cell Signaling), anti-LRP1 rabbit [EPR3724] (1:1000; ab215997; Abcam), anti-β1 integrin goat (1:200; AF2405; R&D).

Image acquisition, processing, and analysis

Confocal microscopy was performed using a confocal microscope (SP8; Leica). For image analysis, the Fiji software was used (open source, http://fiji.sc/). The figures were assembled and processed using Adobe Photoshop and Adobe Illustrator. The only adjustments used in the preparation of the figures were for brightness and contrast. For comparison purposes, different sample images of the same antigen were acquired under constant acquisition settings.

To quantify *Gpr126* mRNA expression levels using fluorescence in situ hybridization (FISH) in the brain microvasculature, an automated custom Fiji plugin (18) was developed. The number of single molecule RNA (smRNA) of *Gpr126* in the vessels assessed by *Cldn5* positive regions was measured on the maximum projected images. *Cldn5* regions were identified on the 488-channel using the Li's threshold (ttps://imagej.net/plugins/auto-threshold#li) method after maximum filtering, rolling ball background subtraction and Gaussian filtering. Within these regions the number of smRNA of *Gpr126* were identified using the 'Find Maxima' tool after 'rolling ball' subtraction on the 647-channel.

To quantify *Gpr126* and *Lrp1* mRNA expression levels using FISH on the adherent cells cultured on coverslips, the presence of smRNA was estimated as the ratio between the number of nuclei and the smRNA in a field, using a custom Fiji custom script.

To identify the smRNA of *Gpr126* or *Lrp1* on the 647-channel the 'Find Maxima' algorithm was used after 'rolling-ball' background removal and a 'median' filter. The nuclei marked with DAPI were found using StarDist[4] with the built-in Versatile (fluorescent nuclei) model, after Gaussian filtering.

In vivo cadaverine leakage was measured as follows. Each mouse brain sagittal section was acquired under 10× magnification. Then, using Fiji, eight regions of interest (ROIs) were defined (three in the cortex, one in the olfactory bulb, cerebellum, midbrain, pons, medulla, hypothalamus, striatum). The ROI sizes were defined to roughly cover 80% of the area of the region. The mean intensities of each ROI were measured. The size and the relative position of the ROIs were kept constant across each comparison. Two sections for each sample were used for quantification. Quantification of the leakage was carried out using two distinct operators. The first operator acquired the confocal stacks, while the second operator performed the quantification in a blind test.

Quantification of brain vessel average width was determined using two custom Fiji plugins. The first plugin generated a distance map as an intermediate result, using the local thickness tool after the Huang thresholding method on the image filtered with a median and Gaussian filter, in which the vessels were stained for podocalyxin. The second plugin applied a grid on the local thickness distance map and for each tile determined the vessel average width as the mean intensity of the pixels with a value above 0 (where intensities represent the distances on the local thickness distance map).

The numbers of microcapillaries in the brains stained with Pecam-1 were assessed using a custom Fiji plugin. This tool identified and counted the microcapillaries using the triangle thresholding method on the sum projection of the PECAM-1 channel filtered with a Median filter.

The vessel (podocalyxin, PECAM-1) coverage by pericyte (PDGFRβ, CD13) and BM (fibronectin, collagen IV, laminin α2) was evaluated using a semi-automated Fiji plugin on the maximum projected image. Vessel region was identified by manual thresholding after rolling ball background subtraction, maximum and median filtering. Within this region, the coverage area (pericytes or BM) was quantified by manual thresholding after rolling ball background subtraction and Gaussian filtering.

PLVAP and claudin-5 mean intensities in the vessel area (assessed by podocalyxin) were quantified using a custom Fiji plugin. This tool identified the vessel areas using the triangle thresholding method on podocalyxin channel filtered with a median and maximum filter after rolling ball background subtraction. Within the vessel area, the claudin-5 intensity is measured after background correction, which was estimated using the mean intensity of a region drawn by hand.

Quantification of tip cells in the retina was carried out by two separate operators. The first operator acquired the confocal stacks, and the second operator manually counted tip cells in a blind test. Cell counting was through the cell counter plugin of Fiji.

The radial expansion in the retina was measured as the ratio between the mean distance covered by the vessels (growing from the optic nerve) and the total length of the petal. The lengths were measured by hand using the line tool of Fiji on the maximum projected image.

The vessel density in the retina was determined using a semi-automated Fiji plugin. This tool quantified the vessel density as the ratio between the area covered by vessels (colored pixels of stained vessels) and the whole retina region (dark pixels of the retina tissue) on the maximum projected image. The whole retina region area was identified by manual thresholding and Fill Holes binary operation after a large Gaussian filtering. Within this region, the vessel area was identified by manual thresholding after median filtering.

Quantification of the EdU EC nuclei in the retina was carried out on the maximum projected image using a custom automated Fiji plugin. The vessel area (CD93 staining) was defined using the Li thresholding method after filtering the image with a median filter. Within this area the plugin identified the nuclei EC area (ERG staining) using the Otsu thresholding method, after filtering the image with median and Gaussian filters. The double ERG- and EdU-positive nuclei were counted with the ImageJ Find Maxima tool within the vessel area.

To measure the ratio between the Ki67- or BrdU-positive cells and ERG-positive cBECs, a custom Fiji plugin was developed. The plugin counted the ERG-positive nuclei using the Yen's threshold method on the sum projected image, after median and Gaussian filtering. Within the ERG-positive nuclei, the plugin counted the cells on the Ki67 and BrdU staining using the same process.

Antibodies

The antibodies used in this study were: anti-GPR126 rabbit (1:500, ab75456; Abcam; WB; IF), anti-GPR126 rabbit (1:10, Ab218046; Abcam; EM) anti-GAPDH mouse (1:1000, sc32233; Santa Cruz; WB), anti-Podocalyxin goat (1:200, AF1556; R&D; IF), anti-Tubulin mouse (1:2000, T9026; Sigma-Aldrich; WB), anti-Vinculin mouse (1:5000, V9131; Sigma-Aldrich; WB), anti-phospho-CREB rabbit S133 (1:1000, 9198; Cell Signaling; WB), anti-CREB rabbit (1:1000, 4820; Cell Signaling; WB), anti-Myc mouse (1:1000, 2276; Cell Signaling; WB), anti-Myc rabbit (1:1000, 2272; Cell Signaling; WB), anti-c-Myc agarose affinity gel antibody rabbit (A7470; Sigma-Aldrich; IP), anti-c-Myc agarose affinity gel antibody mouse (20168; Thermo Fisher Scientific; IP) anti-LRP1 mouse (1:1000; ab215997; Abcam; WB), anti-LRP1 rabbit [EPR3724] (1:1000; ab215997; Abcam; WB), anti-LRP1 α-chain mouse B411E2 (1:10000; WB) (19), anti-LRP1 rabbit β-chain 1704 (1:1000; WB) (20), anti-β1 integrin goat (1:200; AF2405; R&D; IP, WB), anti-β1 integrin rabbit (1:1000; 4706; Cell Signaling; WB), rat anti-mouse β1 integrin (1:50, 553715; BD Biosciences, active β1, FACS, IF); alexa Fluor 647 anti-mouse/rat β1 integrin (1:50, 102214; Biolegend, FACS); anti-β1 integrin (1:10, AF 2325; R&D Systems; EM), anti-α1 integrin goat (1:500; ab243032; Abcam; WB), anti-α3 integrin goat (1:3500; AF2787; R&D; WB), Horse anti-rabbit biotinylated IgG (1:400; VC-BA-1100-MM15; Vector Laboratories; IF), Alexa Fluor 555 conjugate Streptavidin (1:400; S32355; Invitrogen; IF), anti-GFP chicken (1:1000; ab13970; Abcam; IF), anti-PECAM-1 goat (1:200; AF3628; R&D Systems; IF), anti-PECAM-1 rat (1:200; 550274; BD Pharmingen™; IF), Cy3-conjugated monoclonal anti–α-SMA mouse (1:500; C6198; Sigma-Aldrich; IF) and anti-Isolectin-B4 (1:200; B-1205; Vector Laboratories; IF); Alexa Fluor 488 conjugated anti-

PECAM-1 (1:50, FAB3628G; R&D Systems; FACS, IF); anti-Claudin-5 (1:200, Ab15106; Abcam; IF) and PLVAP (1:200, 550563 clone MECA-32; BD Biosciences, IF); HRP-linked anti-mouse and anti-rabbit (1:2000; Cell Signaling; WB), AlexaFluor 555-conjugated donkey anti-rabbit (1:400; Invitrogen; IF); AlexaFluor 488-conjugated donkey anti-goat (1:400; Invitrogen; IF); Cy3-donkey anti-rat IgG (1:200, Jackson Secondary Antibody, 712-165-153; FACS).

Supplemental Figures

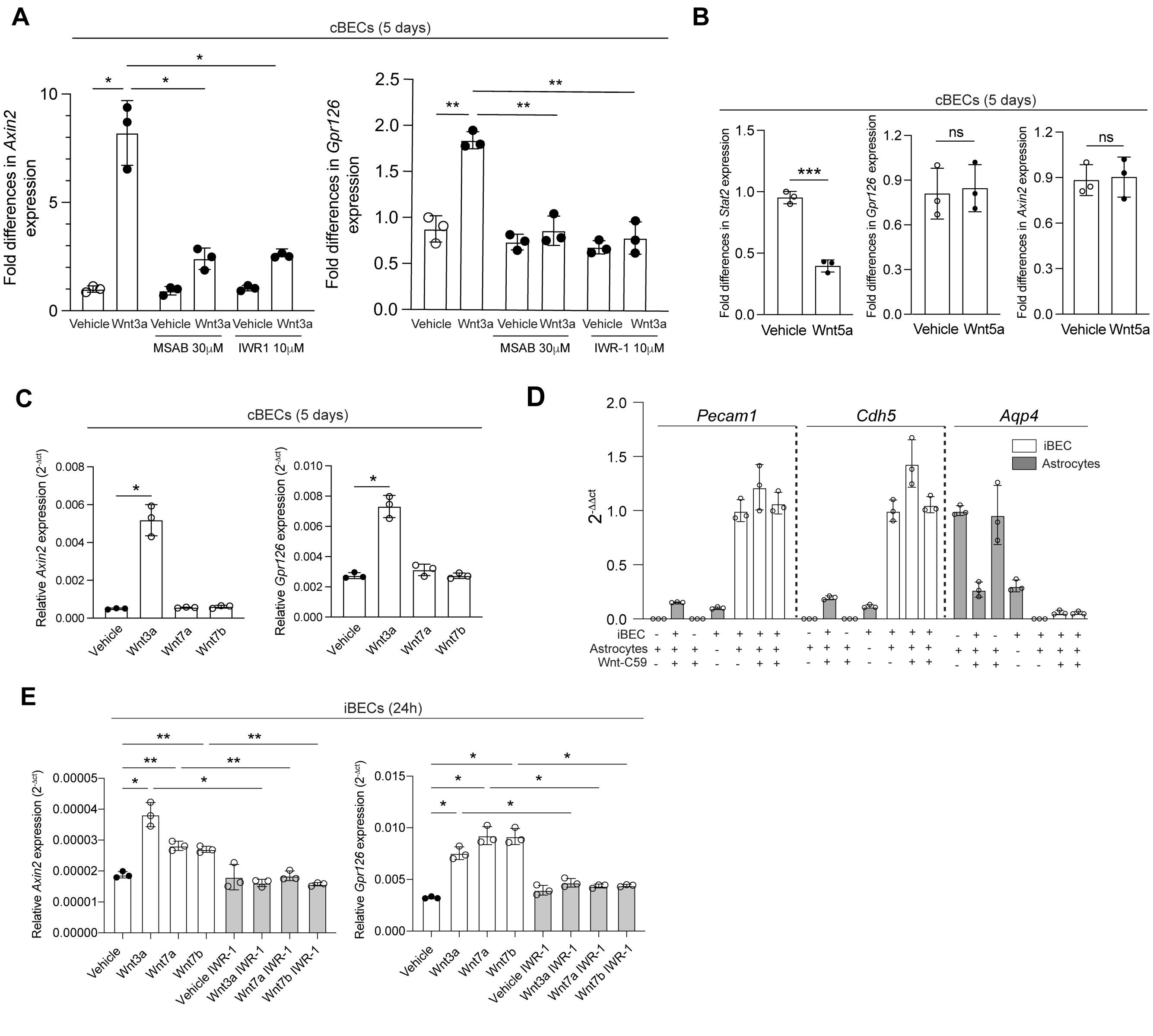

Supplemental Figure 1. GPR126 is a target of the canonical Wnt signaling.

(**A**) Quantification of fold differences in *Axin2* and *Gpr126* gene expression in primary murine endothelial cells derived from the brain microvasculature in culture (cBECs) from adult WT mice following RT-qPCR analysis. The cells were treated with either vehicle or Wnt3a in the presence or in absence of of MSAB (30 μM) or IWR1 (10 μM). Each symbol represents a single experiment (n=3 WT mice for each independent experiment, as means ±SD). *, *P*<0.05; **, *P*<0.01 (Brown-Forsythe and Welch ANOVA, Dunnett's T3 multiple comparisons tests). (**B**) Quantification of fold differences in

Axin2, Gpr126 and *Stat2* gene expression in cBECs from adult WT mice following RT-qPCR analysis. The cells were treated with recombinant Wnt5a, or vehicle as control every other day for a duration of 5 days. Each symbol represents a single experiment (n=4 WT mice for each independent experiment, as means ±SD). ***, *P*<0.001; (unpaired t-tests with Welch's correction). (**C**) Quantification of relative expression of *Axin2* and *Gpr126* in cBECs from adult WT mice, treated with Wnt3a, Wnt7a, Wnt7b or vehicle as a control, every other day for a duration of 5 days. Each symbol represents a single experiment (n=3 WT mice for each independent experiment, as means ±SD). *, *P*<0.05 (Brown-Forsythe and Welch ANOVA, Dunnett's T3 multiple comparisons tests). (**D**) Quantification of relative expression of *Pecam1*, *Cdh5* and *Aqp4* immortalized brain endothelial cells (iBECs) and astrocytes pre-treated or not with Wnt-C59, following RT-qPCR analysis. Each symbol represents a single experiment (n=3 for each independent experiment, as means ±SD). (**E**) Quantification of relative *Axin2* and *Gpr126* expression in iBECs from WT mice. The plate was coated with gelatin embedded purified Wnt3a, Wnt7a and Wnt7b (100 ng/mL) and vehicle as control. iBEC were cultured on for 24 h in the presence of IWR-1 (10 μM) and vehicle as control. Each symbol represents a single experiment (n=3 for each independent experiment, as means ±SD). **P*<0.05; ***P*<0.01 (Brown-Forsythe and Welch ANOVA, Dunnett's T3 multiple comparisons tests).

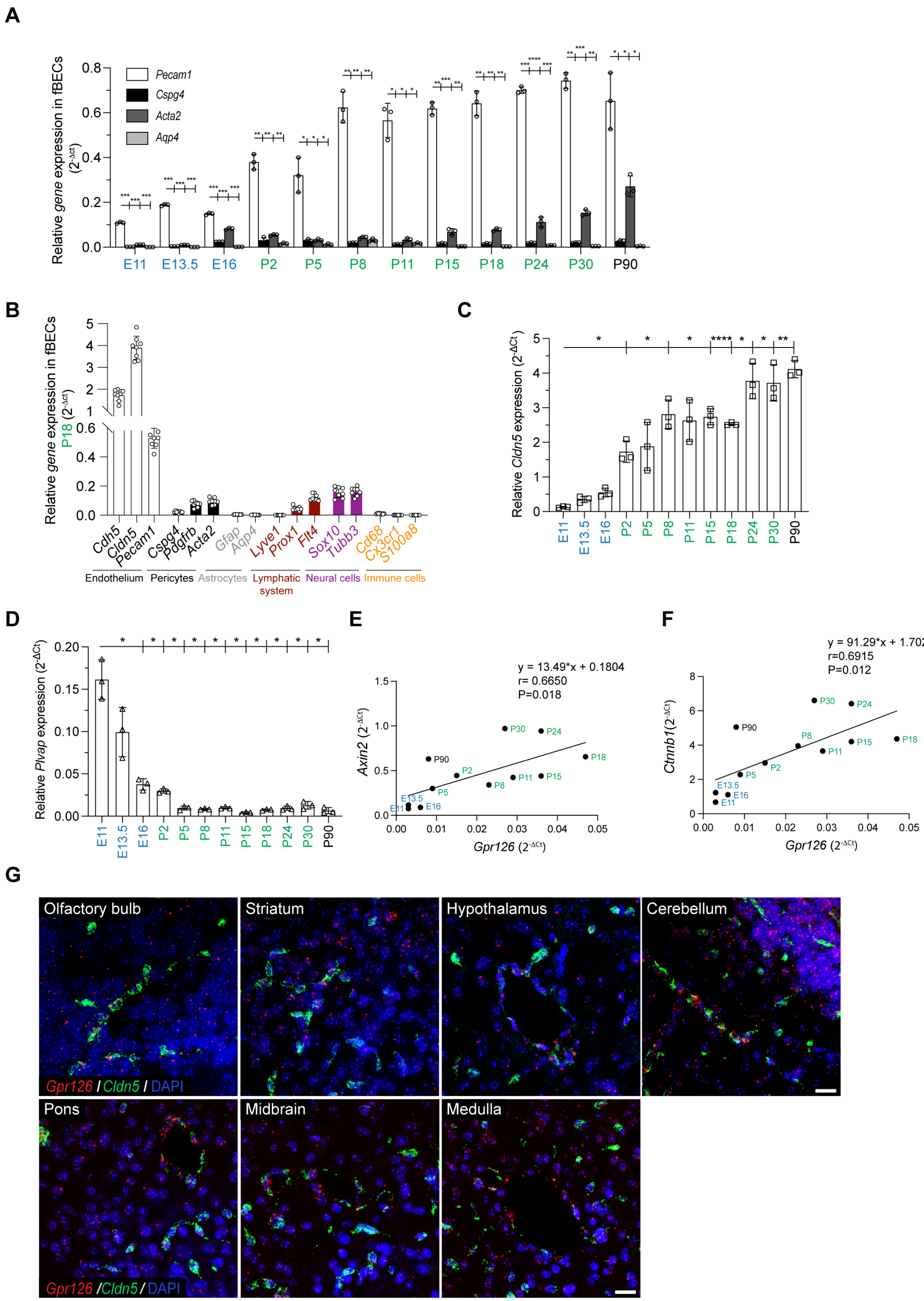
A
Relative gene expression in fBECs (2^-Δct)
Pecam1
Cspg4
Acta2
Aqp4
E11 E13.5 E16 P2 P5 P8 P11 P15 P18 P24 P30 P90
B
Relative gene expression in fBECs P18 (2^-Δct)
Cdh5 Cldn5 Pecam1 Cspg4 Pdgfrb Acta2 Gfap Aqp4 Lyve1 Prox1 Flt4 Sox10 Tubb3 Cd68 Cx3cr1 S100a8
Endothelium
Pericytes
Astrocytes
Lymphatic system
Neural cells
Immune cells
C
Relative Cldn5 expression (2^-ΔCt)
D
Relative Plvap expression (2^-ΔCt)
E
y = 13.49*x + 0.1804
r= 0.6650
P=0.018
Axin2 (2^-ΔCt)
Gpr126 (2^-ΔCt)
F
y = 91.29*x + 1.702
r=0.6915
P=0.012
Ctnnb1(2^-ΔCt)
Gpr126 (2^-ΔCt)
G
Olfactory bulb
Striatum
Hypothalamus
Cerebellum
Gpr126 / Cldn5 / DAPI
Pons
Midbrain
Medulla
Gpr126 /Cldn5 / DAPI

Supplemental Figure 2. GPR126 is expressed in the brain microvasculature during BBB development.

(**A**) Quantification of relative expression of *Pecam1* (ECs), *Cspg4* (pericytes), *Acta2* (smooth muscle cells) and *Aqp4* (astrocytes) in freshly isolated brain endothelial cells (fBECs) from WT mice at different stages during embryonic (E11-E16), postnatal (P2-P30) development, and in the adult (P90) following RT-qPCR analysis. Each symbol represents a single experiment (n=8 mice for embryos, n=5 mice for P2-P5 and n=3 mice for P8-P90 for each independent experiment, as means ±SD). *, *P*<0.05; **, *P*<0.01; ***, *P*<0.001 (Brown-Forsythe and Welch ANOVA, Dunnett's T3 multiple comparisons tests). (**B**) Quantification of relative expression of *Cdh5, Cldn5* and *Pecam1* (ECs), *Cspg4, Pdgfrb,* and *Acta2* (pericytes), *Gfap* and *Aqp4* (astrocytes), *Lyve1*, *Prox1* and *Flt4* (lymphatic system), *Sox10* and *Tubb3* (neural cells), *CD68*, *Cx3cr1* and *S100a8* (immune cells) in fBECs from WT mice at P18, following RT-qPCR analysis. Each symbol represents a single mouse (n=8 mice for each independent experiment, as means ±SD). (**C**) Quantification of relative *Cldn5* expression in fBECs from WT mice at different stages during embryonic (E11-E16), postnatal (P2-P30) development and in the adult (P90) following RT-qPCR analysis. Each symbol represents a single experiment (n=8 mice for embryos, n=5 mice for P2-P5 and n=3 mice for all the rest time points for each independent experiment, as means ±SD). *, *P*<0.05; **, *P*<0.01; ****, *P*<0.0001 (Brown-Forsythe and Welch ANOVA, Dunnett's T3 multiple comparisons tests). (**D**) Quantification of relative *Plvap* expression in fBECs from WT mice at different stages during embryonic (E11-E16), postnatal (P2-P30) development and in the adult (P90) following RT-qPCR analysis. Each symbol represents a single experiment (n=8 mice for embryos, n=5 mice for P2-P5 and n=3 mice for all the rest time points for each independent experiment, as means ±SD). *, *P*<0.05 (Brown-Forsythe and Welch ANOVA, Dunnett's T3 multiple comparisons tests). (**E, F**) Pearson´s correlation analysis (r) of gene expression in fBECs from WT mice at different stages during embryonic (E11-E16), postnatal (P2-P30) development and in the adult (P90) following RT-qPCR analysis. (**E**), Positive correlation between *Gpr126* expression and *Axin2* (p = 0.018, r = 0.6650). (**F**) Positive correlation between *Gpr126* expression and *Ctnnb1 (*p = 0.012, r = 0.6915). (**G**) Representative confocal images of RNA scope *in-situ* hybridization for *Gpr126* (red) and *Cldn5* (green) mRNA in different regions of mouse brain at P18. DAPI (blue) stains nuclei. Scale bar: 20 µm.

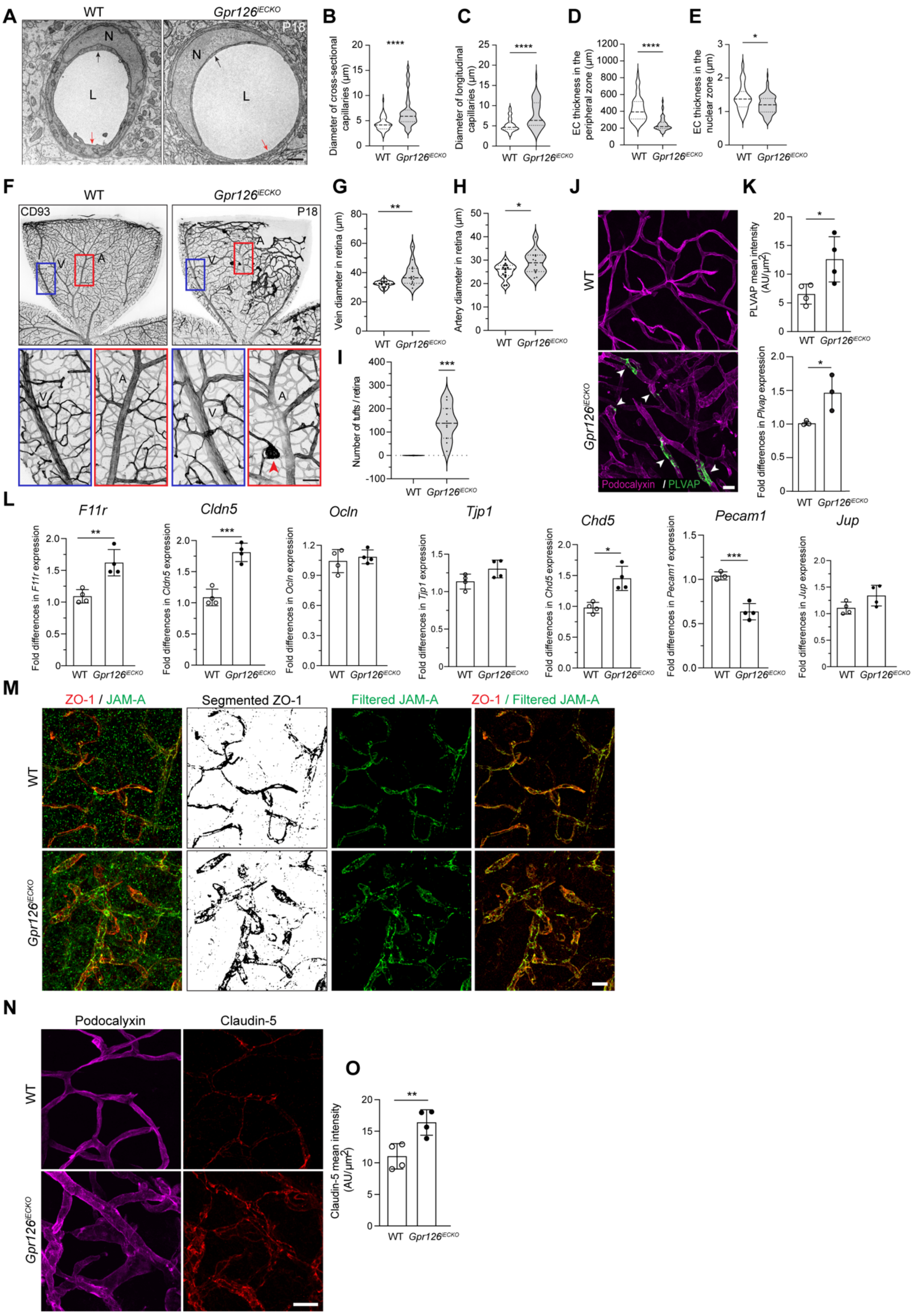
A
WT
Gpr126iECKO
P18
N
L
B
Diameter of cross-sectional capillaries (μm)
C
Diameter of longitudinal capillaries (μm)
D
EC thickness in the peripheral zone (μm)
E
EC thickness in the nuclear zone (μm)
F
CD93
G
Vein diameter in retina (μm)
H
Artery diameter in retina (μm)
I
Number of tufts / retina
J
Podocalyxin / PLVAP
K
PLVAP mean intensity (AU/μm2)
Fold differences in Plvap expression
L
F11r
Cldn5
Ocln
Tjp1
Chd5
Pecam1
Jup
M
ZO-1 / JAM-A
Segmented ZO-1
Filtered JAM-A
ZO-1 / Filtered JAM-A
N
Podocalyxin
Claudin-5
O
Claudin-5 mean intensity (AU/μm2)

Supplemental Figure 3. The endothelial GPR126 is required for the formation of the correct retina and brain vasculature.

(**A**) Representative electron microscopy of cross sections of blood capillaries in WT and *Gpr126*^{iECKO} mice. Images were acquired at the same magnification. Black arrows, luminal perinuclear zone; red arrows, peripheral zone of the EC. Scale bar: 1 µm. L, lumen, N, nucleus. (**B-E**) Quantification of cross-sectional (**B**) and longitudinal (**C**) diameters, and EC thickness in the peripheral zone (**D**) and in the nuclear zone (**E**) (n=3 WT and 3 *Gpr126*^{iECKO} mice, as means ±SD). *, *P* <0.05; ****, *P* <0.00005 (unpaired t-tests with Welch's correction). (**F**) Representative confocal images for CD93 in the retina of WT and *Gpr126*^{iECKO} mice at P18. Scale bar: 500 µm. Bottom: Blue and red rectangles, magnified retina regions; red arrowhead, a tuft malformation in the retinal vasculature of *Gpr126*^{iECKO} mouse. V, vein, A, artery. Scale bar: 200 µm. (**G, H**) Quantification of vein (**G**) and artery (**H**) diameters in retina of WT and *Gpr126*^{iECKO} mice at P18. Each symbol represents a single retina (n=7 WT and 7 *Gpr126*^{iECKO} mice, as means ±SD). *, *P* <0.05; **, *P* <0.01 (unpaired t-tests with Welch's correction). (**I**) Quantification of the number of tuft malformations in retina of WT and *Gpr126*^{iECKO} mice at P18. Each symbol represents a single retina. (n=6 WT and 6 *Gpr126*^{iECKO} mice, as means ±SD). ***, *P* <0.001 (Wicoxon signed rank tests). (**J**) Representative confocal images for Podocalyxin (ECs, magenta) and PLVAP (fenestrated vessels, green) in brain cortex vibratome sections (100 µm) of WT and *Gpr126*^{iECKO} mice at P18. The arrowheads indicate the presence of PLVAP staining. Scale bar: 20 µm. (**K**) Quantification of PLVAP signal (mean intensity) in the vessels area of brain cortex cryosections, as shown in (**J**) and expressed as arbitrary units (AU). Each symbol represents a mean of 6 sections for each mouse (n=4 WT and 4 *Gpr126*^{iECK}O mice, as means ±SD). *, *P* <0.05 (unpaired t-tests with Welch's correction). Bottom: quantification of fold differences in *Plvap* gene expression in fBECs from WT and *Gpr126*^{iECKO} pups at P18. Each symbol represents a mouse. (n=3 WT and 3 *Gpr126*^{iECKO} mice, as means ±SD). *, *P* <0.05 (unpaired t-tests with Welch's correction). (**L**) Quantification of the fold differences in F11 receptor (*F11r*), claudin-5 (*Cldn5*), occludin (*Ocln*), tight junction protein 1 (*Tjp1*), VE-cadherin (*Cdh5*), platelet and endothelial cell adhesion molecule-1 (*Pecam1*) and junction plakoglobin (*Jup*) gene expression in fBECs from WT and *Gpr126*^{iECKO} pups at P18. Each symbol represents a mouse (n=3-5 WT and 3-5 *Gpr126*^{iECKO} mice, as means ±SD). *, *P*<0.05; **, *P*<0.01; ***, *P*<0.001; (unpaired t-tests with Welch's correction). (**M**) Representative confocal images for zonula occludens-1 (ZO-1; red) and junctional adhesion molecule-A (JAM-A; green) in brain cortex vibratome sections (100 µm) of WT and *Gpr126*^{iECKO} mice at P18. ZO-1 staining was segmented with threshold 350-4.096 (Segmented ZO-1) for

merged images of filtered JAM-A (ZO-1/filtered JAM-A) Scale bar: 20 µm. (n=3, WT and n=3, *Gpr126*^{iECKO}). (**N**) Representative confocal images for podocalyxin (magenta) and claudin-5 (red) in brain cortex vibratome sections (100 µm) of WT and *Gpr126*^{iECKO} mice at P18. Scale bar: 20 µm. (**O**) Quantification of claudin-5 signal (mean intensity) in the vessels area of brain cortex cryosections, as shown in **N** and expressed as arbitrary units (AU). Each symbol represents a mean of 6 sections for each mouse (n=4 WT and 4 *Gpr126*^{iECKO} mice, as means ±SD). *, P <0.05 (unpaired t-tests with Welch's correction).

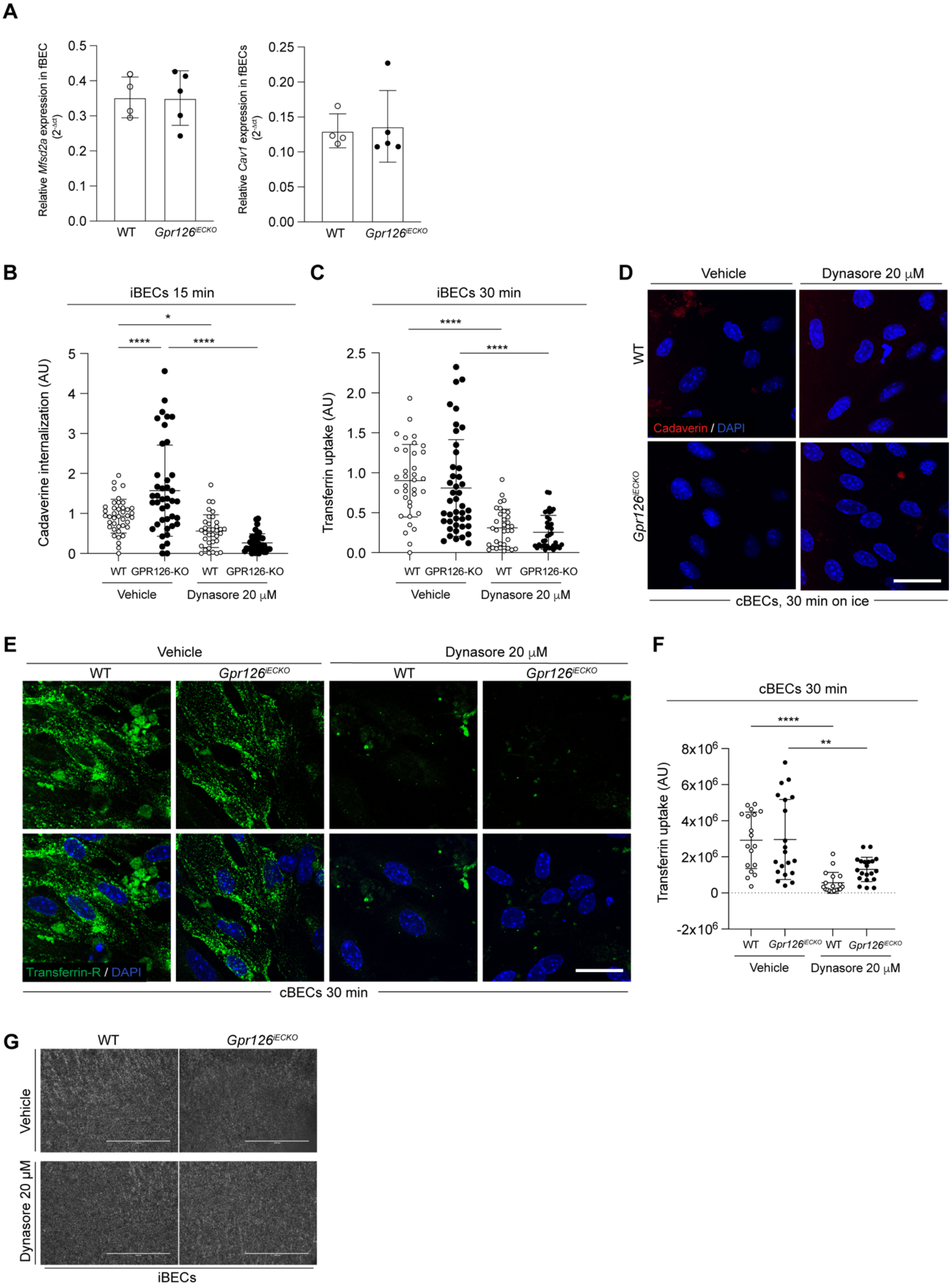
A
Relative Mfsd2a expression in fBEC (2^-Δct)
WT
Gpr126iECKO
Relative Cav1 expression in fBECs (2^-Δct)
B
iBECs 15 min
Cadaverine internalization (AU)
WT GPR126-KO WT GPR126-KO
Vehicle
Dynasore 20 μM
C
iBECs 30 min
Transferrin uptake (AU)
D
Vehicle
Dynasore 20 μM
Cadaverin / DAPI
cBECs, 30 min on ice
E
Transferrin-R / DAPI
cBECs 30 min
F
cBECs 30 min
Transferrin uptake (AU)
G
iBECs

Supplemental Figure 4. The permeability induced by GPR126 inactivation is mediated by enhanced activity of the transcellular route.

(**A**) Quantification of relative expression of *Mfsd2a* and *Cav1* in fBECs from WT and *Gpr126*^{iECKO} pups at P18, following RT-qPCR analysis. Each symbol represents a mouse (n=4 WT mice and n=5 *Gpr126*^{iECKO} mice, as means ±SD). (unpaired t-tests with Welch's correction). (**B**) WT and GPR126-KO iBECs, were treated with Dynasore (20μM) or vehicle as control, for 30 minutes. Cells were then incubated with cadaverine (red) for 15 minutes for the uptake assay. Acid wash was performed prior fixation to remove cadaverine at the plasma membrane. Quantification of the relative internalized cadaverine fluorescence intensity, expressed as arbitrary units (AU), in WT and GPR126-KO iBECs is shown. Each symbol represents a single field/10 cell (as means ±SD). *, $P<0.05$; ****, $P<0.0001$; (one-way ANOVA and Sidak's multiple comparison test, assuming a single pooled variance). (**C**) WT and GPR126-KO iBECs were treated as in (**B**) and then incubated with transferrin (green) for 30 minutes for the uptake assay. Quantification of the relative internalized transferrin fluorescence intensity, expressed as arbitrary units (AU), in WT and GPR126-KO iBECs is shown. Each symbol represents a single field/10 cell (as means ±SD). ****, $P<0.0001$; (one-way ANOVA and Sidak's multiple comparison test, assuming a single pooled variance). (**D**) Representative confocal images of WT and *Gpr126*^{iECKO} cBECs treated with Dynasore for 30 minutes, or vehicle as a control. Images show cadaverine (red) at 4°C (a condition that typically prevents endocytosis) after 30 min. DAPI stains nuclei. Scale bar: 30 μm. (**E**) Representative confocal images of WT and *Gpr126*^{iECKO} cBECs treated with Dynasore for 30 minutes, or vehicle as a control. Images show transferrin-R endocytosis (green) after 30 min. Acid wash was performed prior fixation to remove cadaverine at the PM. DAPI stains nuclei. Scale bar: 30 μm. (**F**) Quantification of the relative internalized transferrin fluorescence intensity, expressed as arbitrary units (AU), in WT and *Gpr126*^{iECKO} cBECs, as shown in (**E**). Each symbol represents a single field/ 10 cells (n=9 WT, n=6 *Gpr126*^{iECKO}, as means ±SD). **, $P<0.01$; ****, $P<0.0001$; (one-way ANOVA and Sidak's multiple comparison test, assuming a single pooled variance). (**G**) Representative crystal violet staining of filters from WT and GPR126-KO iBEC monolayers in the presence or absence of Dynasore as shown in Figure 2L. Monolayers were stained at the end of the transcytosis assay (120 min). Scale bar: 1000 μm (quintuplicate filters/condition; n=5)

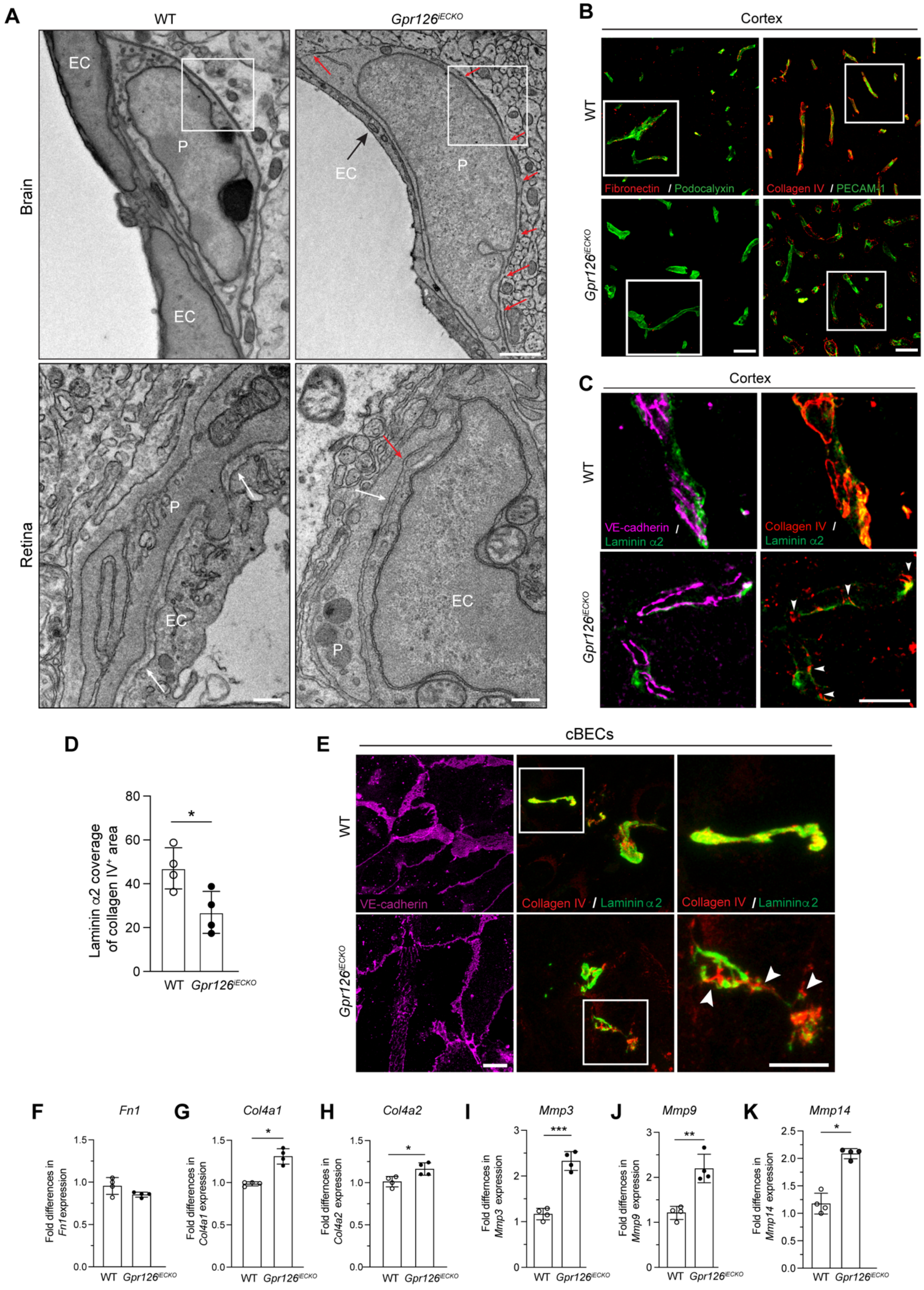
A
WT
Gpr126iECKO
Brain
Retina
EC
P
B
Cortex
Fibronectin / Podocalyxin
Collagen IV / PECAM-1
C
VE-cadherin / Laminin α2
Collagen IV / Laminin α2
D
Laminin α2 coverage of collagen IV+ area
E
cBECs
VE-cadherin
F
Fn1
Fold differences in Fn1 expression
G
Col4a1
H
Col4a2
I
Mmp3
J
Mmp9
K
Mmp14

Supplemental Figure 5. GPR126 is required for proper basal membrane protein deposition in the brain.

(**A**) Representative tomography virtual slices of brain (upper panels) and retina (lower panels) blood capillary longitudinal cortex section from WT and *Gpr126*^{iECKO} mice at P18. White arrows indicate regular thickness of the basal membrane. Red arrows indicate decreased thickness or absence of the BM. White boxes show regions that are magnified in Figure 3 A. EC= endothelial cell; P=pericyte. Scale bar (upper panels): 1 µm. Scale bar (lower panels): 330 nm. (**B**) Representative confocal images of brain cortex cryosections (4 µm) from WT and *Gpr126*^{iECKO} mice at P18. For the vasculature, podocalyxin (green), or PECAM-1 (green), were used. The BM was stained for fibronectin (red) or collagen IV (red). Merged images are shown. White boxes show magnified regions reported in Figure 3B. Scale bar: 200 µm. (**C**) Representative confocal images of brain cortex cryosections (4 µm) from WT and *Gpr126*^{iECKO} mice at P18. For the vasculature VE-cadherin (magenta) is shown. The BM was stained for laminin α2 (green). Collagen IV/Laminin α2: arrowheads show absence of colocalization of the two proteins. Scale bar: 100 µm. (**D**) Quantification of collagen IV coverage of laminin α2-positive areas, as shown in (**C**). Each symbol represents a mouse (n=4 WT and 3 or 4 *Gpr126*^{iECKO} mice, as means ±SD). *, $P<0.05$; **, $P<0.005$ (unpaired t-tests with Welch's correction). (**E**) Representative confocal images for VE-cadherin (magenta), collagen IV (red) and laminin α2 (green) in cBECs from WT and *Gpr126*^{iECKO} mice at P18. White boxes show magnified region depicted on the right. Collagen IV/laminin α2: arrowheads show the absence of colocalization of the two proteins. Scale bar: 10 µm. (**F-K**) Quantification of fold differences in fibronectin (*Fn1*), collagen4a1 (*Col4a1*), collagen4a2 (*Col4a2*), matrix metalloprotease 3 (*Mmp3*), matrix metalloprotease 9 (*Mmp9*) and matrix metalloprotease 14 (*Mmp14*) gene expression in fBECs from WT and *Gpr126*^{iECKO} mice at P18. Each symbol represents a mouse (n=4 WT and 4 *Gpr126*^{iECKO} mice, as means ±SD). *, $P<0.05$; **, $P<0.01$; ***, $P<0.001$; (unpaired t-tests with Welch's correction).

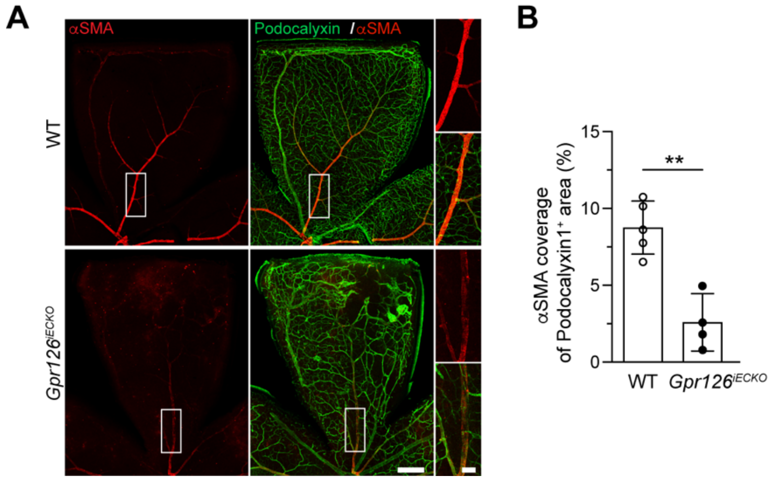

Supplemental Figure 6. GPR126 is required for proper pericytes coverage in the retina.

(**A**) Representative confocal images for α-smooth muscle actin, (αSMA veins; red) and podocalyxin (green) in WT and *Gpr126*^{iECKO} mouse retinas at P18. The same petal with a different staining is shown in Figure 3J. Scale bar: 500 µm. White rectangles are shown in magnification on the right of each image. Scale bar: 100 µm. (**B**) Quantification of αSMA coverage of podocalyxin-positive area, expressed as percentage, as shown in (**A**). Each symbol represents a retina for each mouse. (n=5 WT and 4 *Gpr126*^{iECKO} mice, as means ±SD). **, *P*<0.01 (unpaired t-tests with Welch's correction).

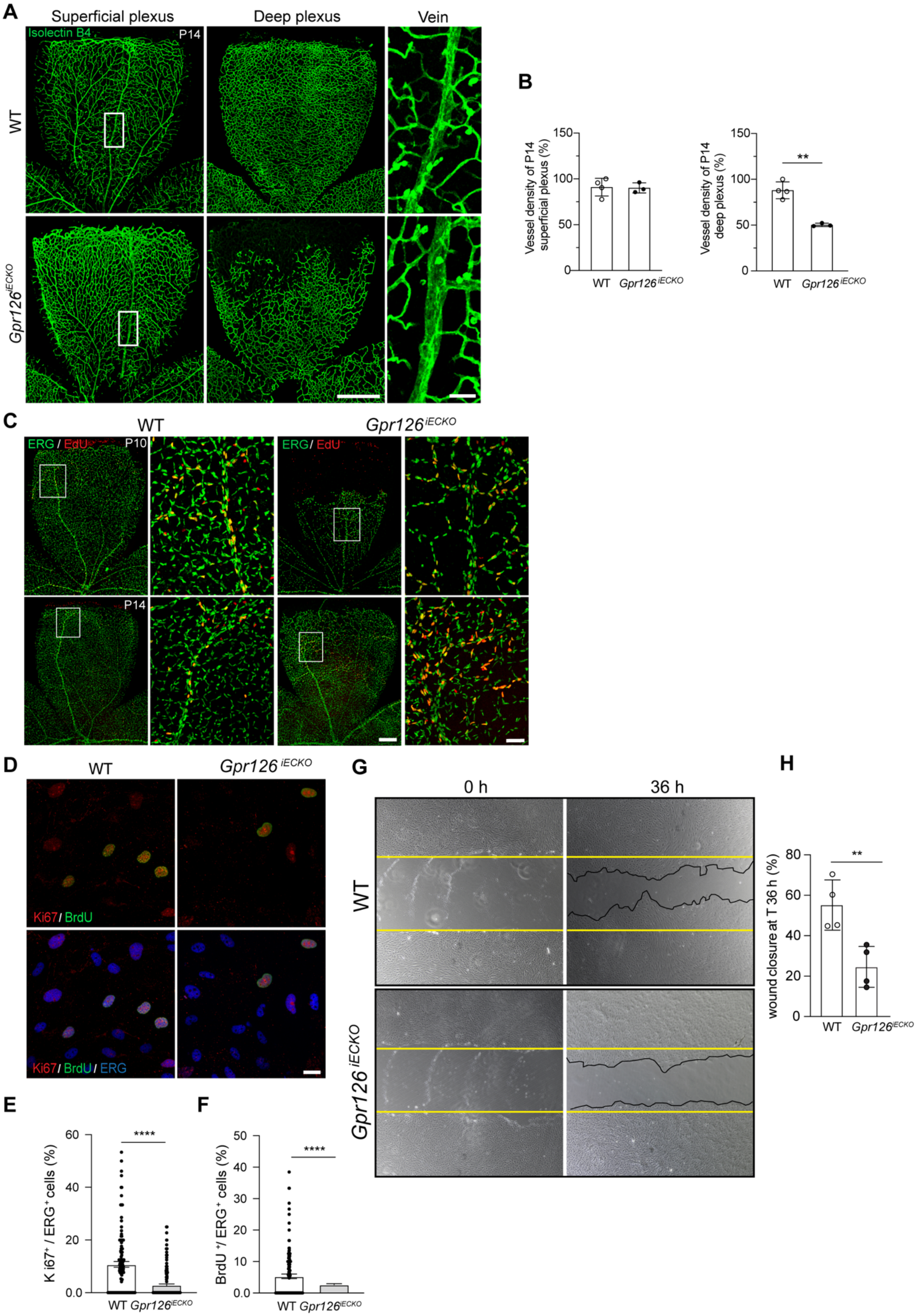
A
Superficial plexus
Deep plexus
Vein
Isolectin B4
P14
WT
Gpr126 iECKO
B
Vessel density of P14 superficial plexus (%)
Vessel density of P14 deep plexus (%)
**
WT
Gpr126 iECKO
C
WT
Gpr126 iECKO
ERG / EdU
P10
P14
D
WT
Gpr126 iECKO
Ki67 / BrdU
Ki67 / BrdU / ERG
E
K i67+ / ERG+ cells (%)
****
F
BrdU +/ ERG+ cells (%)
****
G
0 h
36 h
WT
Gpr126 iECKO
H
wound closure at T 36 h (%)
**

Supplemental Figure 7. GPR126 is required for the correct development of the retinal vasculature that supports EC proliferation and migration.

(**A**) Representative confocal images for isolectin B4 (green) in the superficial or the deep retinal vasculature plexus from WT and *Gpr126*^{iECKO} mice at P14. Scale bar: 500 µm. White boxes are shown in magnification on the right of each image. Scale bar: 50 µm. (**B**) Quantification of the vessel density superficial and deep plexus of the retinas, as shown in (**A**), expressed as percentage. Each symbol represents a retina for each mouse. (n=4 WT and 3 *Gpr126*^{iECKO} mice, as means ±SD). **, *P*<0.01; (unpaired t-tests with Welch's correction). (**C**) Representative confocal images for ERG (EC nuclei, green) and EdU (proliferating cell nuclei, red) in WT and *Gpr126*^{iECKO} mouse retinas at P10 (upper panels) and P14 (lower panels). Scale bar: 500 µm. White rectangles are shown in magnification on the right of each image. Scale bar: 100 µm. (**D**) Representative confocal images for Ki67 (proliferating cells, red), BrdU (S phase cells, green) and ERG (EC nuclei, blue) in cBECs from WT and *Gpr126*^{iECKO} mice at P18. Scale bar: 20 µm. (**E-F**) Quantification of the percentage ratio of cells positive for Ki67 (**E**) or BrdU (**F**) to the total number of cells (ERG-positive), as shown in (**D**). Each symbol represents a mean of 24 fields for each mouse (n=4 WT and 4 *Gpr126*^{iECKO} mice, as means ±SEM). ****, *P*<0.0001 (unpaired t-tests with Welch's correction). (**G**) Representative phase-contrast images of the scratch wound migration assay at time 0 and 36 h post-scratch performed in cBECs from WT and *Gpr126*^{iECKO} mice at P18. The yellow lines indicate the edges of the wound at time 0. The punctuated black lines indicate the boundaries of the wound after 36 h. (**H**) Quantification of the wound closure after 36 h to the total width of the wound at time 0, expressed as a percentage. Each symbol represents a single experiment (n=20 WT and 20 *Gpr126*^{iECKO} mice for each independent experiment, as means ±SD). **, *P*<0.01 (unpaired t-tests with Welch's correction).

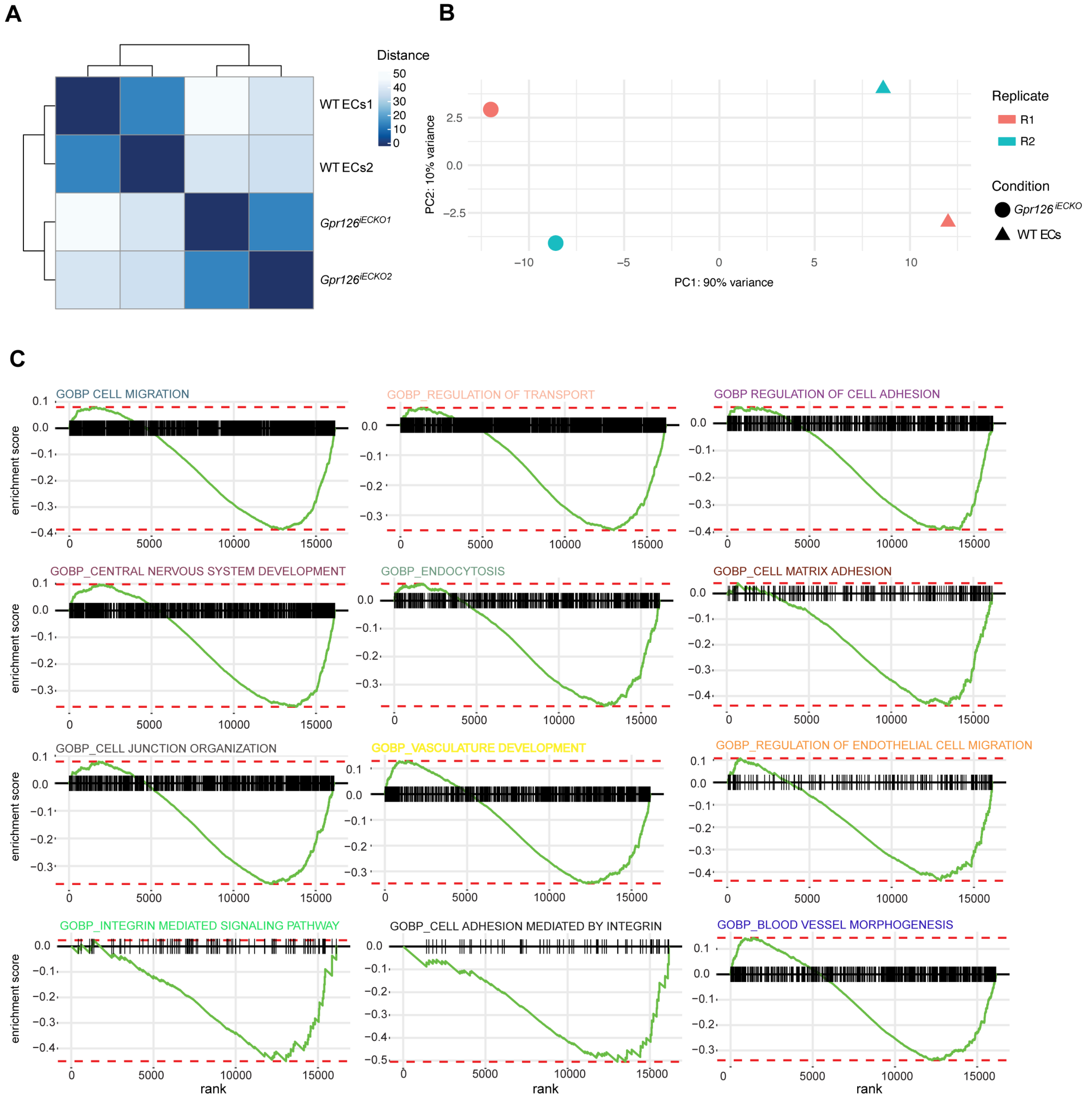

Supplemental Figure 8. Quality control of RNA-seq data.

(**A, B, C**) fBECs were isolated from WT and *Gpr126*^{iECKO} mice at P18 and the mRNA was processed for bulk RNA-sequencing. (**A**) Distance matrix heatmap showing hierarchical clustering of sample-to-sample distances among the four samples that are the 2 biological replicates of each condition (WT ECs and *Gpr126*^{iECKO}). (**B**) Principal component analysis (PCA) plot showing the samples in the 2D plane

spanned by their first two principal components. Each symbol represents a biological replicate (R1, R2) for each condition (n=4, 2 WT [circle] and 2 *Gpr126*^{iECKO} [triangle] mice). (**C**) Gene Set Enrichment Analysis (GSEA) plot of selected Gene Ontology Biological Process terms (GOBP). The green curve corresponds to the enrichment score (ES) curve, which is the running sum of the weighted enrichment score obtained from the fGSEA software.

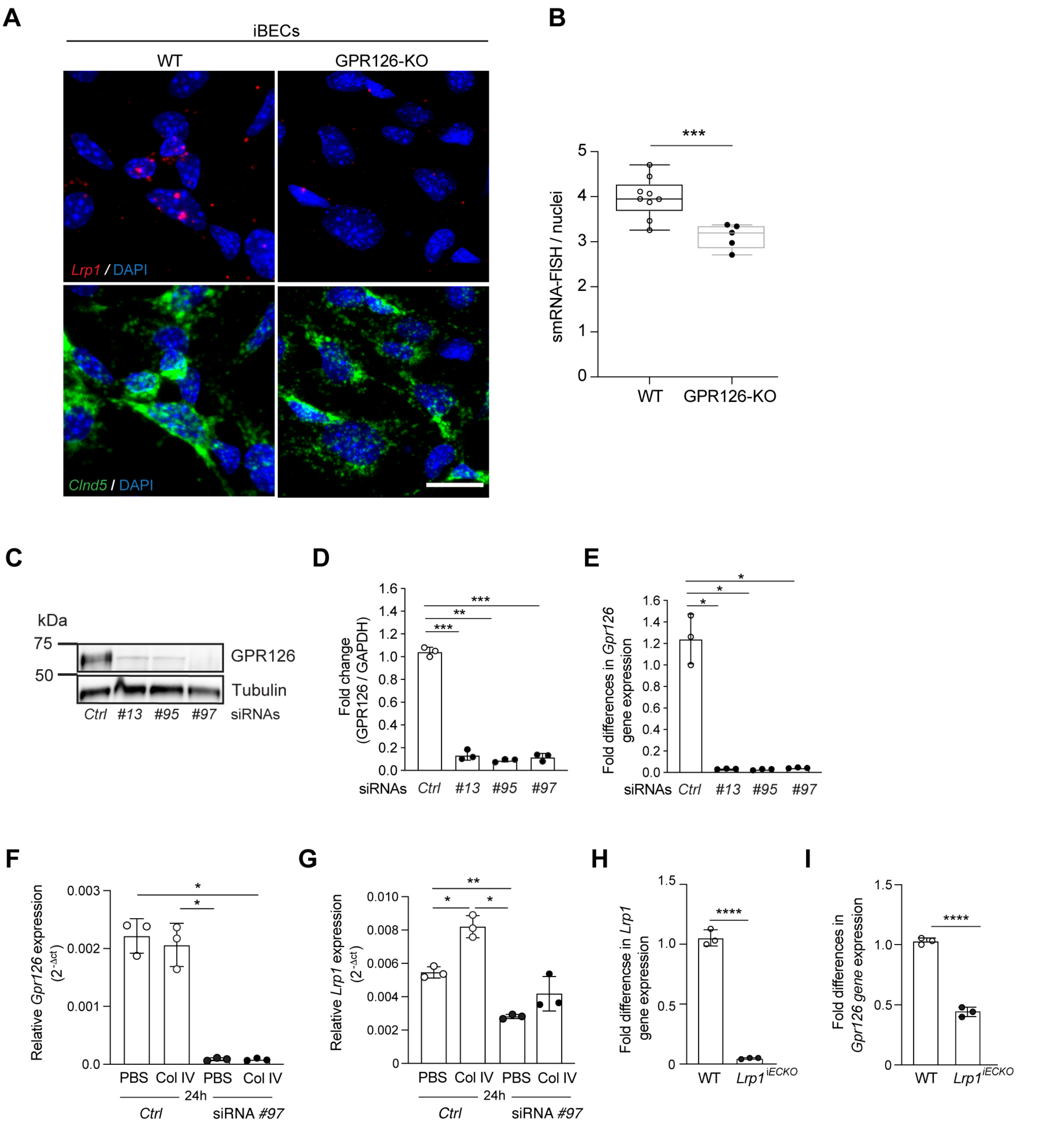

Supplemental Figure 9. LRP1 is a novel target of the GPR126 signaling pathway.

(**A**) Representative confocal images of FISH for *Lrp1* (red) and *Cldn5* (green) mRNA in WT and GPR126-KO iBECs. DAPI stains nuclei. Scale bar: 20 µm. (**B**) Quantification of single molecules RNA of *Lrp1* per nuclei as shown in (**A**). Each symbol represents a single field/40 cell (as means ±SD). ***,

P<0.001 (unpaired t-tests with Welch's correction). (**C**) Representative immunoblotting for GPR126 in cBECs isolated from WT pups at P18 transfected with nontargeting small-interfering RNA (siRNA; *Ctrl* [control]) or with siRNAs against GPR126 (*#13*, *#95*, *#97*). Tubulin is shown as loading control. (**D)** GPR126/GAPDH ratio quantified by densitometry scanning and expressed as fold change, as shown in **C**. Each symbol represents a single experiment (n=12 WT mice for each independent experiment, as means ±SD). **, *P* <0.01; ***, *P* <0.001 (Brown-Forsythe and Welch ANOVA, Dunnett's T3 multiple comparison tests). (**E**) Quantification of the relative gene expression of *Gpr126* in cBECs isolated from WT pups at P18, as shown in (**C**). Each symbol represents a single experiment (n=12 WT mice for each independent experiment, as means ±SD). *, *P*<0.05; Brown-Forsythe and Welch ANOVA, Dunnett's T3 multiple comparison tests). (**F**) Quantification of the relative gene expression of *Gpr126* in cBECs isolated from pups at P18, as shown in (**D**), except for the treatment with collagen IV for 24 h. Each symbol represents a single experiment (n=12 WT mice for each independent experiment, as means ±SD). *, *P*<0.05; (Brown-Forsythe and Welch ANOVA, Dunnett's T3 multiple comparison tests). (**G**) Quantification of relative gene expression of *Lrp1* in cBECs isolated from pups at P18, as shown in **F**. Each symbol represents a single experiment (n=3 WT and 3 $Gpr126^{iECKO}$ mice for each independent experiment, as means ±SD). *, *P*<0.05; **, *P*<0.01; (Brown-Forsythe and Welch ANOVA, Dunnett's T3 multiple comparison tests). (**H**) Quantification of fold differences in gene expression of *Lrp1* in cBECs from adult WT and $Lrp1^{iECKO}$ mice. Each symbol represents a single experiment (n=3 WT and 3 $Gpr126^{iECKO}$ mice for each independent experiment, as means ±SD). ****, *P*<0.0001 (unpaired t-tests with Welch's correction). (**I**) Quantification of fold differences in gene expression of *Gpr126* in cBECs from adult WT and $Lrp1^{iECKO}$ mice. Each symbol represents a single experiment (n=3 WT and 3 $Gpr126^{iECKO}$ mice for each independent experiment, as means ±SD). ****, *P*<0.0001 (unpaired t-tests with Welch's correction).

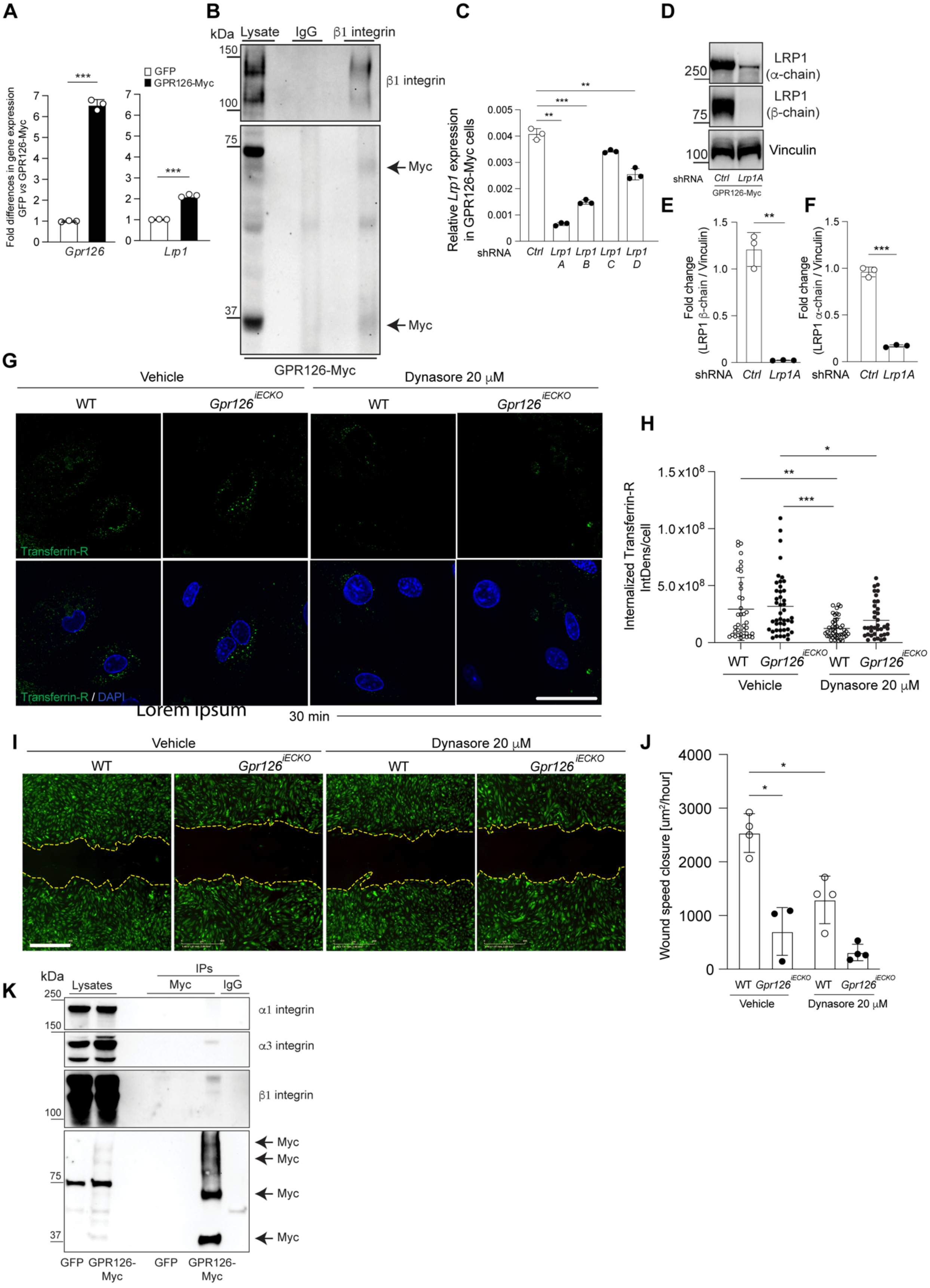
A
Fold differences in gene expression GFP vs GPR126-Myc
GFP
GPR126-Myc
Gpr126
Lrp1
B
kDa
Lysate
IgG
β1 integrin
β1 integrin
Myc
Myc
GPR126-Myc
C
Relative Lrp1 expression in GPR126-Myc cells
shRNA
Ctrl
Lrp1 A
Lrp1 B
Lrp1 C
Lrp1 D
D
LRP1 (α-chain)
LRP1 (β-chain)
Vinculin
shRNA
Ctrl
Lrp1A
GPR126-Myc
E
Fold change (LRP1 β-chain / Vinculin)
shRNA
Ctrl
Lrp1A
F
Fold change (LRP1 α-chain / Vinculin)
shRNA
Ctrl
Lrp1A
G
Vehicle
Dynasore 20 μM
WT
Gpr126 iECKO
Transferrin-R
Transferrin-R / DAPI
Lorem ipsum
30 min
H
Internalized Transferrin-R IntDens/cell
WT
Gpr126 iECKO
Vehicle
Dynasore 20 μM
I
Vehicle
Dynasore 20 μM
WT
Gpr126 iECKO
J
Wound speed closure [um²/hour]
WT
Gpr126 iECKO
Vehicle
Dynasore 20 μM
K
kDa
Lysates
IPs
Myc
IgG
α1 integrin
α3 integrin
β1 integrin
Myc
GFP
GPR126-Myc

Supplemental Figure 10. GPr126 form a complex with LRP1 and β1 integrin and sustains EC migration.

(**A**) Quantification of fold differences in *Gpr126* and *Lrp1* expression in iBECs expressing GFP (GFP) and transfected with GPR126-Myc (GPR126-Myc). following RT-qPCR analysis. (n=3, as means±SD). ***, *P*<0.001 (unpaired t-tests with Welch's correction). (**B**) Representative immunoblotting for β1 integrin and Myc in iBECs transfected with GPR126-Myc. The protein extracts (lysates) were immunoprecipitated with an anti-β1 integrin antibody [IPs (β1 integrin)] or IgG as control. Data are representative of 3 independent experiments. (**C**) Quantification of relative expression of *Lrp1* in GPR126-Myc cells treated with *scramble*-shRNA (Ctrl) and four different shRNA for *Lrp1* (*Lrp1 A*, *B, C, D*), following RT-qPCR analysis. Each symbol represents a single experiment (n=3 for each independent experiment, as means ±SD). **, *P*<0.01; ***, *P*<0.001; (Brown-Forsythe and Welch ANOVA, Dunnett's T3 multiple comparisons tests). (**D**) Representative immunoblotting for LRP1 (α-chain and β-chain) in GPR126-Myc cells, treated with sh#*scramble* (*Ctrl*) and *Lrp1A* RNAs. Vinculin is shown as the loading control. (**E**) LRP1 β-chain /vinculin ratio quantified by densitometry scanning and expressed as fold change, as show in (**D**). Each symbol represents a single experiment (n=3 Ctrl and 3 *Lrp1A* for each independent experiment, means ±SD). **, *P*<0.01 (unpaired t-tests with Welch's correction). (**F**) LRP1 α-chain /vinculin ratio quantified by densitometry scanning and expressed as fold change, as show in (**D**). Each symbol represents a single experiment (n=3 *Ctrl* and 3 *Lrp1A* for each independent experiment, means ±SD). ***, *P*<0.001 (unpaired t-tests with Welch's correction). (**G**) Representative confocal images of WT and $Gpr126^{iECKO}$ cBECs treated with Dynasore for 30 minutes, or vehicle as a control. Images show internalized transferrin (green) after 30 min. Acid wash was performed prior fixation to remove cadaverine at the PM. DAPI stains nuclei. Scale bar: 30 μm. (**H**) Quantification of the relative internalized transferrin fluorescence intensity, expressed as arbitrary units (AU), in WT and $Gpr126^{iECKO}$ cBECs, as shown in (**G**). Each symbol represents fluorescence intensity in each cell (n=9 WT, n=6 $Gpr126^{iECKO,}$ as means ±SD). *, *P*<0.05; **, *P*<0.01; ***, *P*<0.001; (one-way ANOVA and Sidak's multiple comparison test, assuming a single pooled variance). (**I**) Confluent cBECs from WT and $Gpr126^{iECKO}$ mice at P18 were labeled with CellTracker™ Green CMFDA and pre-incubated with vehicle or 20 μM Dynasore. Representative immunofluorescence images of the scratch wound migration assay after 20 h post-scratch. The yellow dashed lines indicate the edges of the wound at time 20. (**J**) Quantification of the speed of closure after 20 hours. Each symbol represents a single experiment (n=8 WT and 7 $Gpr126^{iECKO}$ mice for each independent experiment, as means ±SD). *,

$P<0.05$ (Brown-Forsythe and Welch ANOVA, Dunnett's T3 multiple comparisons tests). (**K**) Representative immunoblotting for α1 integrin, α3 integrin, β1 integrin, and Myc in iBECs transfected with GFP and GPR126-Myc. The protein extracts (lysates) were immunoprecipitated with an anti-myc antibody [IPs (Myc)] or IgG [IPs (IgG)] (only for GPR126-Myc cells), as control. Data are representative of 3 independent experiments.

Supplemental Table I. List of genes present in at least 75% of all the selected GO terms.

Gene	N Pathways	% Pathways
ABL1	11	92%
ITGB1BP1	10	83%
WNT5A	9	75%
VEGFA	9	75%
THBS1	9	75%
PTK2B	9	75%
PTK2	9	75%
NRP1	9	75%
LRP1	9	75%
ITGB3	9	75%
ITGB2	9	75%
ITGAV	9	75%
CDC42	9	75%
TNF	8	67%
THY1	8	67%
TGFB1	8	67%
SYK	8	67%
SRC	8	67%
SFRP1	8	67%
SERPINE1	8	67%
ROCK1	8	67%
RHOA	8	67%
PTN	8	67%
PTEN	8	67%
PIK3CG	8	67%
PIK3CB	8	67%
PDPK1	8	67%
JUP	8	67%
ITGB1	8	67%
ITGA2	8	67%
HRG	8	67%
GREM1	8	67%
FLNA	8	67%
CIB1	8	67%
CEACAM1	8	67%
CDH13	8	67%
BCAS3	8	67%
APOD	8	67%
WNT7A	7	58%
WNT4	7	58%
TGFB2	7	58%
TEK	7	58%
SRF	7	58%
SHH	7	58%
ROCK2	7	58%
RIN2	7	58%
RAC1	7	58%
PRKD1	7	58%
PRKCA	7	58%
PPARG	7	58%
NOTCH1	7	58%
NF1	7	58%
MIR27B	7	58%
MIR27A	7	58%
KDR	7	58%
JAM3	7	58%
ITGA5	7	58%
GATA2	7	58%
FUT1	7	58%
FN1	7	58%
EPHA2	7	58%
EMP2	7	58%
EGF	7	58%
EFNA1	7	58%
DAG1	7	58%
CAV1	7	58%
BMP4	7	58%
APOE	7	58%
ANGPT1	7	58%
AKT1	7	58%
ACVRL1	7	58%
ACTG1	7	58%

Supplemental Movie 1. Gpr126 depletion reduced sprouting spheroids in the angiogenesis assay in vitro. Time-lapse recording of sprouting spheroids from fBECs of WT and *Gpr126*^{iECKO} mice at P18, as shown in Figure 4I. Images were acquired by time-lapse widefield microscopy (Thunder Leica) using a 20× objective. Frames were taken every 30 min for 48 h. Arrows show a sprouting EC over time. Arrowheads show a retracting EC over time.

References

1. Mogha A, Benesh AE, Patra C, Engel FB, Schoneberg T, Liebscher I, et al. Gpr126 functions in Schwann cells to control differentiation and myelination via G-protein activation. *J Neurosci.* 2013;33(46):17976-85.
2. Liebner S, Corada M, Bangsow T, Babbage J, Taddei A, Czupalla CJ, et al. Wnt/beta-catenin signaling controls development of the blood-brain barrier. *J Cell Biol.* 2008;183(3):409-17.
3. Liebner S, Kniesel U, Kalbacher H, and Wolburg H. Correlation of tight junction morphology with the expression of tight junction proteins in blood-brain barrier endothelial cells. *Eur J Cell Biol.* 2000;79(10):707-17.
4. Calabria AR, Weidenfeller C, Jones AR, de Vries HE, and Shusta EV. Puromycin-purified rat brain microvascular endothelial cell cultures exhibit improved barrier properties in response to glucocorticoid induction. *J Neurochem.* 2006;97(4):922-33.
5. Paolinelli R, Corada M, Ferrarini L, Devraj K, Artus C, Czupalla CJ, et al. Wnt activation of immortalized brain endothelial cells as a tool for generating a standardized model of the blood brain barrier in vitro. *PLoS One.* 2013;8(8):e70233.
6. Balconi G, Spagnuolo R, and Dejana E. Development of endothelial cell lines from embryonic stem cells: A tool for studying genetically manipulated endothelial cells in vitro. *Arterioscler Thromb Vasc Biol.* 2000;20(6):1443-51.
7. Morini MF, Giampietro C, Corada M, Pisati F, Lavarone E, Cunha SI, et al. VE-Cadherin-Mediated Epigenetic Regulation of Endothelial Gene Expression. *Circ Res.* 2018;122(2):231-45.
8. Dull T, Zufferey R, Kelly M, Mandel RJ, Nguyen M, Trono D, et al. A third-generation lentivirus vector with a conditional packaging system. *J Virol.* 1998;72(11):8463-71.
9. Dobin A, Davis CA, Schlesinger F, Drenkow J, Zaleski C, Jha S, et al. STAR: ultrafast universal RNA-seq aligner. *Bioinformatics.* 2013;29(1):15-21.
10. Patro R, Duggal G, Love MI, Irizarry RA, and Kingsford C. Salmon provides fast and bias-aware quantification of transcript expression. *Nat Methods.* 2017;14(4):417-9.
11. Love MI, Huber W, and Anders S. Moderated estimation of fold change and dispersion for RNA-seq data with DESeq2. *Genome Biol.* 2014;15(12):550.
12. Sergushichev AA, Loboda AA, Jha AK, Vincent EE, Driggers EM, Jones RG, et al. GAM: a web-service for integrated transcriptional and metabolic network analysis. *Nucleic Acids Res.* 2016;44(W1):W194-200.
13. Beznoussenko GV, Pilyugin SS, Geerts WJ, Kozlov MM, Burger KN, Luini A, et al. Trans-membrane area asymmetry controls the shape of cellular organelles. *Int J Mol Sci.* 2015;16(3):5299-333.
14. Beznoussenko GV, Kweon HS, Sesorova IS, and Mironov AA. Comparison of the Cisterna Maturation-Progression Model with the Kiss-and-Run Model of Intra-Golgi Transport: Role of Cisternal Pores and Cargo Domains. *Int J Mol Sci.* 2022;23(7).
15. Bravi L, Rudini N, Cuttano R, Giampietro C, Maddaluno L, Ferrarini L, et al. Sulindac metabolites decrease cerebrovascular malformations in CCM3-knockout mice. *Proc Natl Acad Sci U S A.* 2015;112(27):8421-6.
16. Corada M, Orsenigo F, Morini MF, Pitulescu ME, Bhat G, Nyqvist D, et al. Sox17 is indispensable for acquisition and maintenance of arterial identity. *Nat Commun.* 2013;4:2609.
17. Heiss M, Hellstrom M, Kalen M, May T, Weber H, Hecker M, et al. Endothelial cell spheroids as a versatile tool to study angiogenesis in vitro. *FASEB J.* 2015;29(7):3076-84.
18. Schindelin J, Arganda-Carreras I, Frise E, Kaynig V, Longair M, Pietzsch T, et al. Fiji: an open-source platform for biological-image analysis. *Nat Methods.* 2012;9(7):676-82.

19. Storck SE, Meister S, Nahrath J, Meissner JN, Schubert N, Di Spiezio A, et al. Endothelial LRP1 transports amyloid-beta(1-42) across the blood-brain barrier. *J Clin Invest.* 2016;126(1):123-36.
20. Pietrzik CU, Busse T, Merriam DE, Weggen S, and Koo EH. The cytoplasmic domain of the LDL receptor-related protein regulates multiple steps in APP processing. *EMBO J.* 2002;21(21):5691-700.
