# Supplementary material for: GPR126 is a specifier of blood-brain barrier formation in the mouse central nervous system
Source: J Clin Invest. 2024 Jun 6;134(15):e165368. doi: 10.1172/JCI165368 (PMC11290973; doi:10.1172/JCI165368)
Supplement: Unedited blot and gel images [file jci-134-165368-s058.pdf]

Full unedited gel for Figure 1F

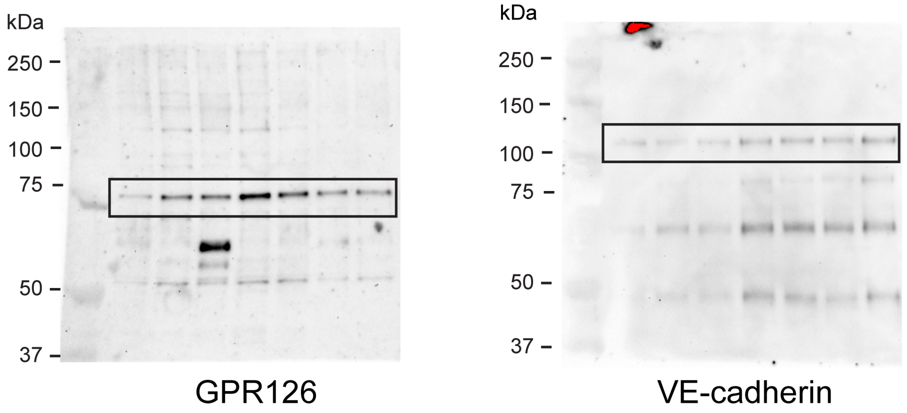

Full unedited gel for Figure 5E

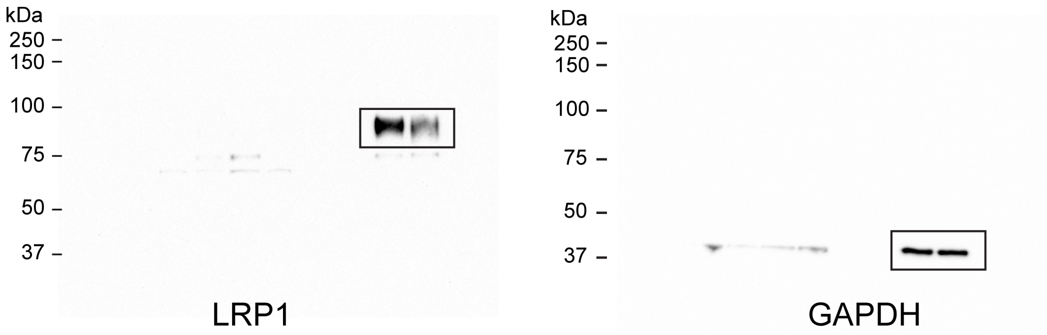

Full unedited gel for Figure 5I

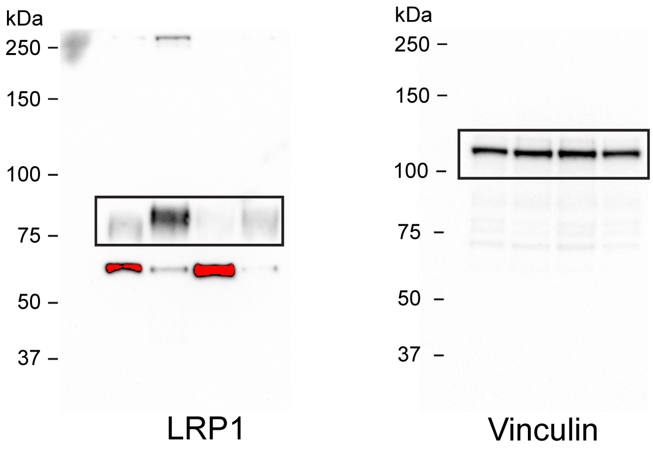

Full unedited gel for Figure 5K

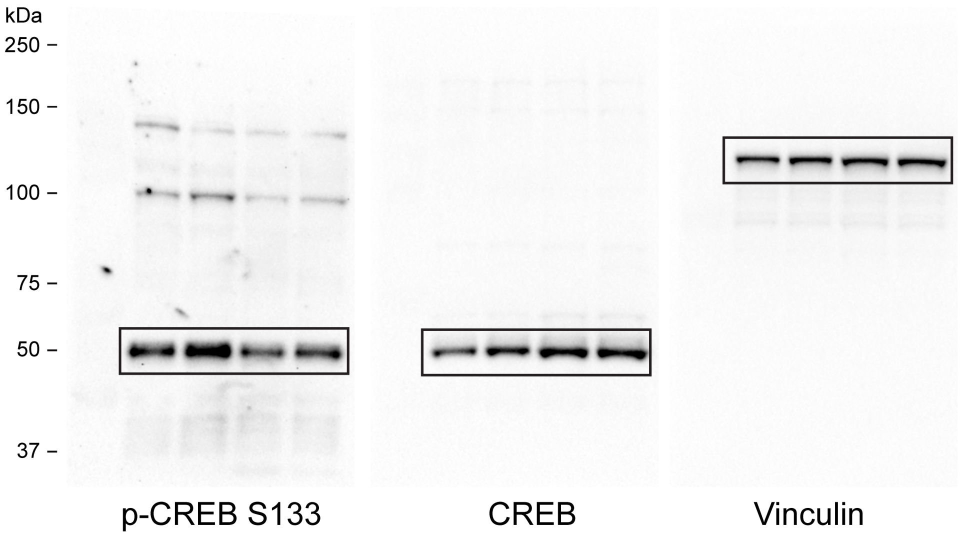

Full unedited gel for Figure 5M

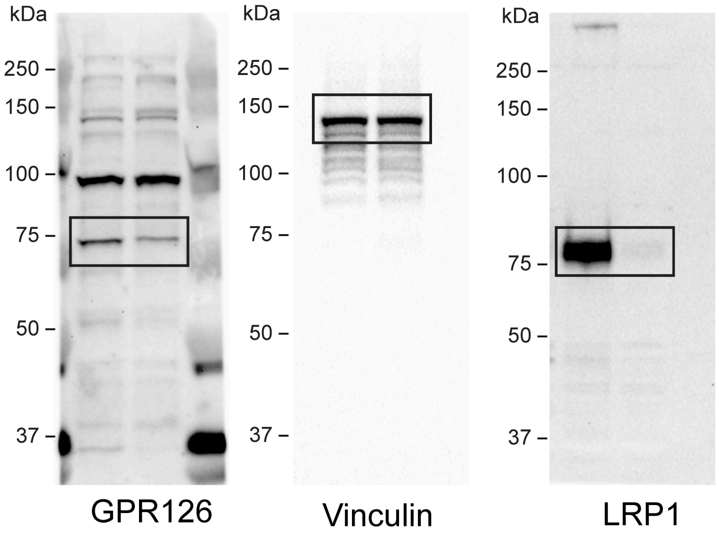

Full unedited gel for Figure 6D

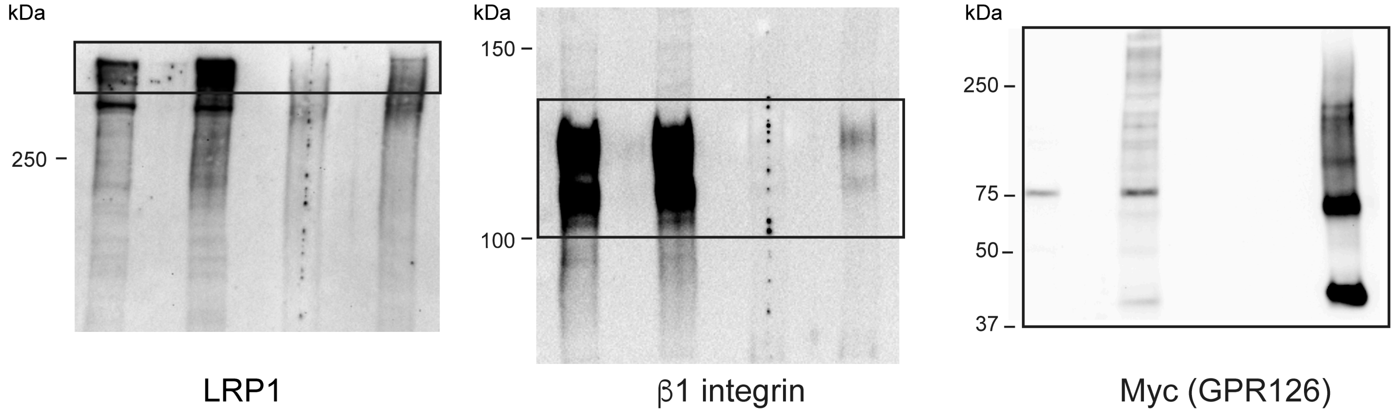

Full unedited gel for Figure 6E

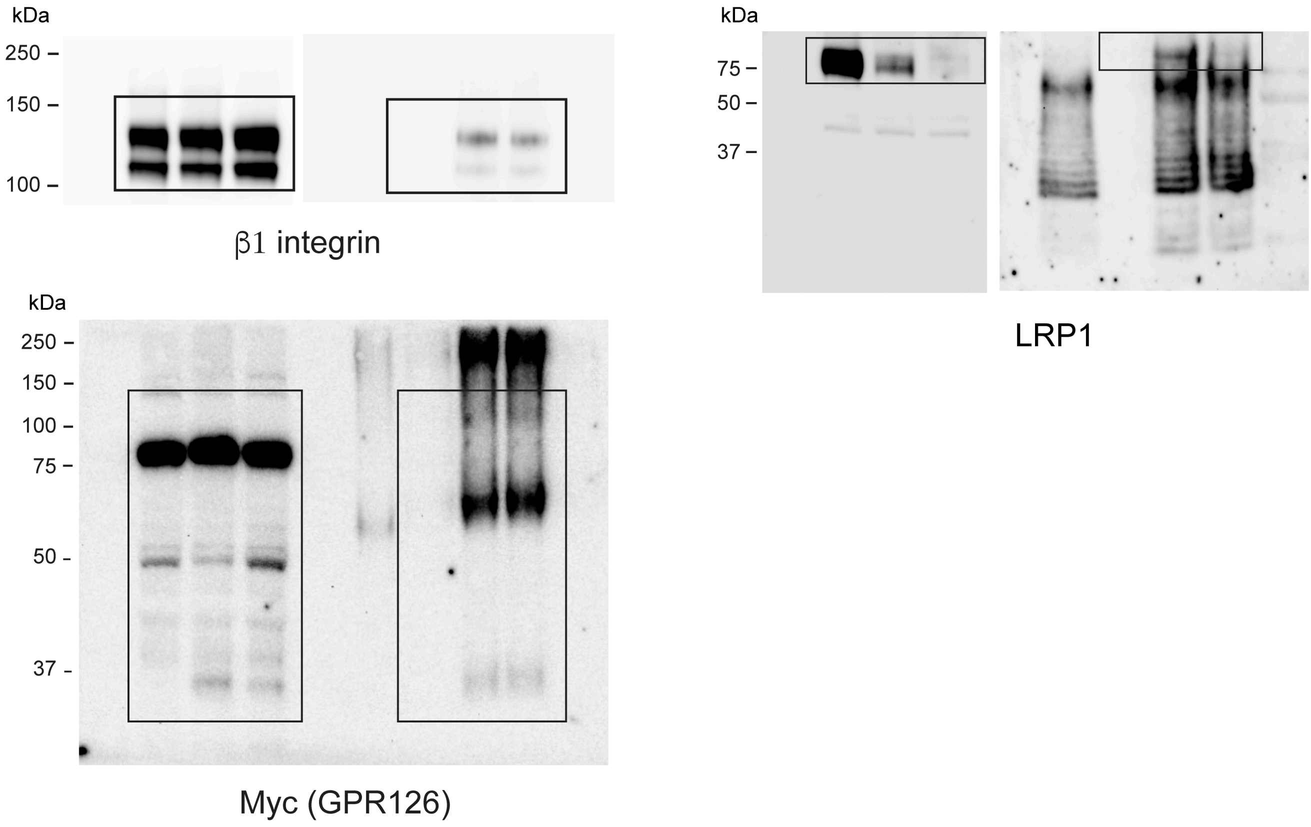

Full unedited gel for Supplementary Figure 9C

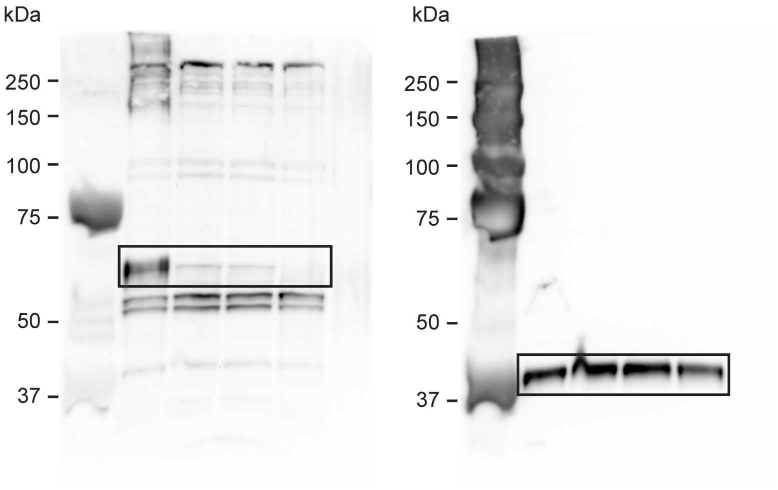

GPR126 Tubulin

Full unedited gel for Supplementary Figure 10B

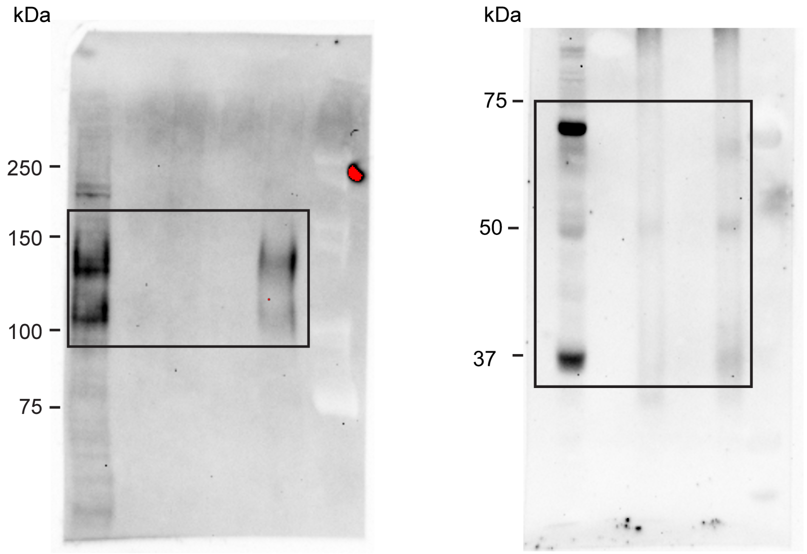

β1 integrin Myc (GPR126)

Full unedited gel for Supplementary Figure 10D

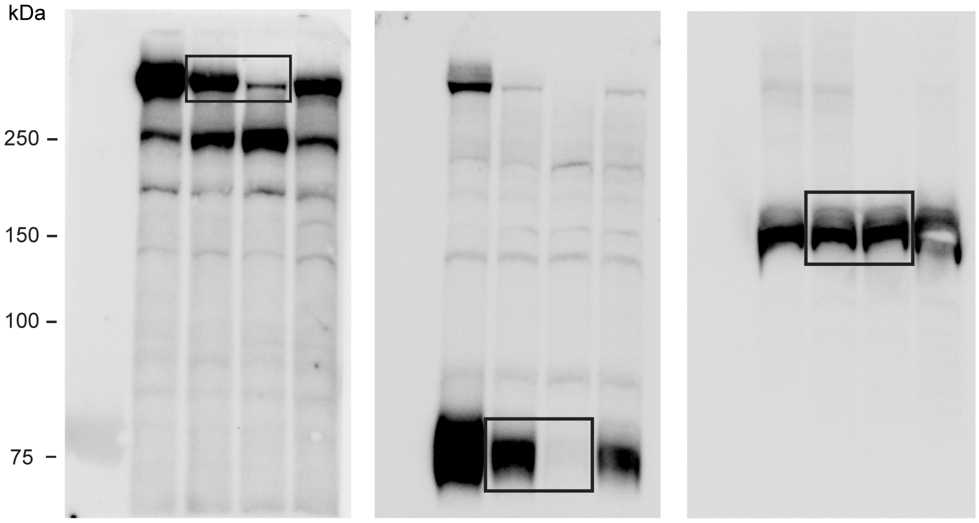

LRP1 (α-chain) LRP1 (β-chain) Vinculin

Full unedited gel for Supplementary Figure 10K

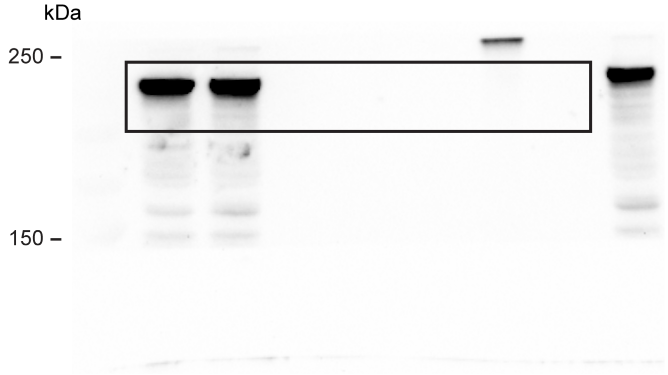

α1 integrin

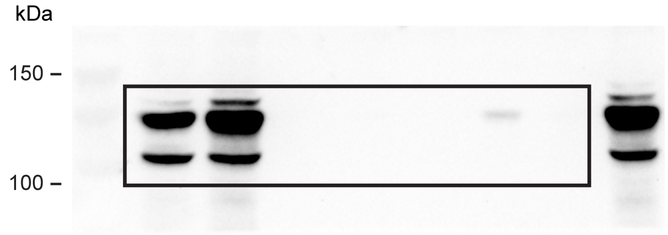

α3 integrin

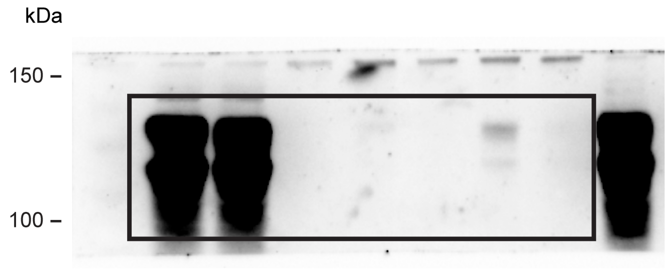

β1 integrin

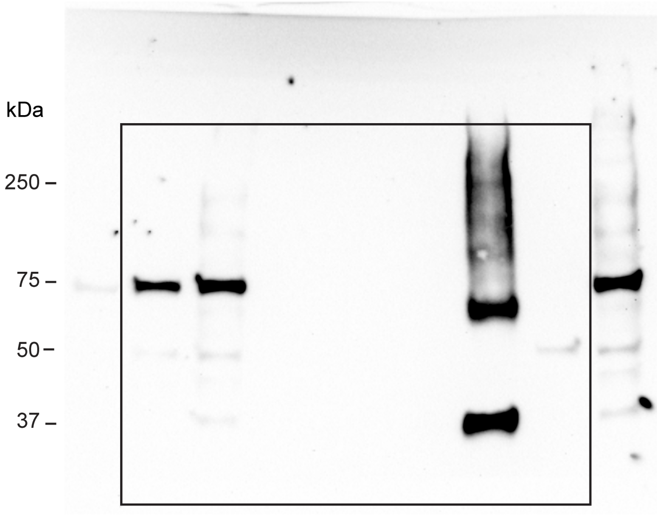

Myc (GPR126)
